# Dual RNA-seq transcriptional analysis of wheat roots colonized by *Azospirillum brasilense* reveals up-regulation of nutrient acquisition and cell cycle genes

**DOI:** 10.1186/1471-2164-15-378

**Published:** 2014-05-16

**Authors:** Doumit Camilios-Neto, Paloma Bonato, Roseli Wassem, Michelle Z Tadra-Sfeir, Liziane CC Brusamarello-Santos, Glaucio Valdameri, Lucélia Donatti, Helisson Faoro, Vinicius A Weiss, Leda S Chubatsu, Fábio O Pedrosa, Emanuel M Souza

**Affiliations:** Department of Biochemistry and Molecular Biology, Universidade Federal do Paraná, Curitiba, PR 81531-990 Brazil; Department of Genetics, Universidade Federal do Paraná, Curitiba, PR Brazil; Department of Cellular Biology, Universidade Federal do Paraná, Curitiba, PR Brazil; Departament of Biochemistry and Biotechnology, Universidade Estadual de Londrina, Londrina, PR Brazil

**Keywords:** RNA-seq, Transcriptional analysis, Wheat, *Triticum aestivum*, *Azospirilum brasilense*, PGPB, Cell cycle and nitrogen fixation

## Abstract

**Background:**

The rapid growth of the world’s population demands an increase in food production that no longer can be reached by increasing amounts of nitrogenous fertilizers. Plant growth promoting bacteria (PGPB) might be an alternative to increase nitrogenous use efficiency (NUE) in important crops such wheat. *Azospirillum brasilense* is one of the most promising PGPB and wheat roots colonized by *A. brasilense* is a good model to investigate the molecular basis of plant-PGPB interaction including improvement in plant-NUE promoted by PGPB.

**Results:**

We performed a dual RNA-Seq transcriptional profiling of wheat roots colonized by *A. brasilense* strain FP2. cDNA libraries from biological replicates of colonized and non-inoculated wheat roots were sequenced and mapped to wheat and *A. brasilense* reference sequences. The unmapped reads were assembled *de novo*. Overall, we identified 23,215 wheat expressed ESTs and 702 *A. brasilense* expressed transcripts. Bacterial colonization caused changes in the expression of 776 wheat ESTs belonging to various functional categories, ranging from transport activity to biological regulation as well as defense mechanism, production of phytohormones and phytochemicals. In addition, genes encoding proteins related to bacterial chemotaxi, biofilm formation and nitrogen fixation were highly expressed in the sub-set of *A. brasilense* expressed genes.

**Conclusions:**

PGPB colonization enhanced the expression of plant genes related to nutrient up-take, nitrogen assimilation, DNA replication and regulation of cell division, which is consistent with a higher proportion of colonized root cells in the S-phase. Our data support the use of PGPB as an alternative to improve nutrient acquisition in important crops such as wheat, enhancing plant productivity and sustainability.

**Electronic supplementary material:**

The online version of this article (doi:10.1186/1471-2164-15-378) contains supplementary material, which is available to authorized users.

## Background

The global human population is projected to be 9 billion by 2050. To meet this rapid growth of the world’s population the predicted demand for food production must increase 1.7 fold by 2050 [1–3]. On the other hand, such increase in food production must be obtained with reduced agricultural inputs, in particular those related to nitrogenous (N) fertilizer, for long term sustainability of food production. Reduction in N fertilizer use will bring at least two benefits: i) reduced emissions of CO_2_ and gaseous N oxides from agricultural processes and ii) decrease of the food production costs, since up to 50% of the operational cost for crop productions arises from N fertilizers [1, 3]. In addition, for the last 40 years crop production increased by 2.4 fold mostly promoted by a 7.4 fold increase in mineral N fertilizers application, which means that the N use efficiency (NUE) has declined 3.1 fold in that time [3, 4].

An alternative to improve NUE is to use plant growth-promoting bacteria (PGPB), since these bacteria are able to increase root-system development and improve acquisition of nutrient including N [2, 3]. Azospirilla is one of the most promising PGPB genera. In Latin America, hundreds of thousands of hectares have been inoculated with *Azospirillum*-based commercial inoculants, increasing grain yields of economically important crops such maize and wheat [5, 6].

Wheat (*Triticum aestivum*) is one of the oldest and widespread crop species. With a production of approximately 630 million tons per year, this crop feeds more than 35% of the world’s population [7, 8]. Significant worldwide efforts of wheat-breeding programs, supported by modern biotechnology, have been applied to increase grain yield, nutritional content, as well as salinity-, drought- and biotic-tolerance [1].

Studies of plant-bacterial interactions (mostly phytopathogens) have been taking advantage from high-throughput techniques and also from the constant improvement of genome sequencing and annotation of both bacteria and plants [9, 10]. However, there is a lack in the application of these recently established techniques in the area of plant-PGPB interaction [6]. Furthermore, although it is well documented that PGPB, in particular *A. brasilense,* can increase plant productivity in several important crops, the mechanism of the plant-bacterial interaction is not entirely understood [6, 11, 12].

Here we performed a dual (plant and bacterium) RNA-seq transcriptional profiling of colonized wheat roots, motivated by the idea that a better understanding of both wheat and *A. brasilense* gene expression might bring insights into: i) the molecular mechanisms of host response; ii) the bacterial colonization strategies; and iii) how to improve plant productivity.

## Results

### Improved growth of wheat seedlings colonized by Azospirillum brasilense

A model of plant-bacterial interaction was set up under axenic conditions. Surface sterilized wheat seeds were germinated on plates of agar/water and transferred to glass tubes containing 25 mL of salt solution (Hoagland’s medium without carbon or nitrogen sources). Wheat seedlings were incubated at 26°C for 24 hours with a light cycle of 14 hours and then inoculated with 0.25 mL of bacterial suspension containing 1.5 × 10^7^ CFU per milliliter. Controls were carried out with non-inoculated wheat seedlings.

**Figure 1:**
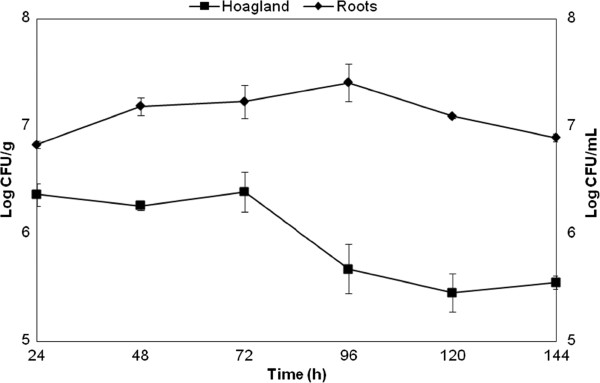
**Kinetics of wheat root colonization by** ***Azospirillum brasilense*** **strain FP2.**

**Figure 2:**
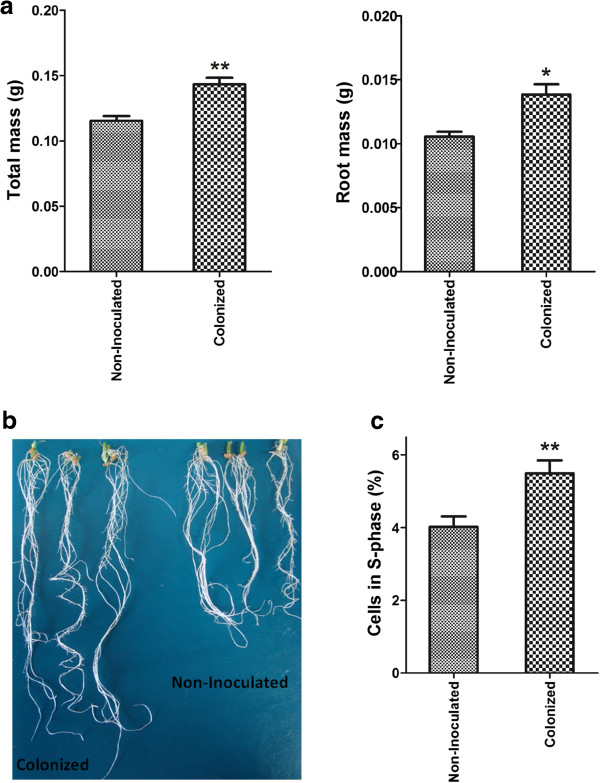
**Improvement of wheat seedlings growth inoculated with** ***Azospirillum brasilense*** **strain FP2. (a)** Total plant mass and root mass eight days after colonization; **(b)** improvement of wheat root size in colonized plants after twenty days of colonization; **(c)** measurement of the number of nuclei synthesizing DNA (S-phase) in root cells stained with propidium iodide three days after colonization. *statistically significant t-test (*p-value* < 0.05%) and **statistically significant t-test (*p-value* < 0.01%).

Bacterial counts showed that three days after inoculation root colonization reached 3.2 × 10^7^ CFU per gram of fresh tissue (Figure 1), but no bacteria were recovered from surface sterilized roots indicating that the *A. brasilense* strain FP2 is an epiphytic colonizer. Root mass of colonized plants increased up to 30% when compared with non-inoculated plants and the total mass of colonized plants increased by 25% compared with non-inoculated plants (Figure 2a). Wheat root size was also enhanced in colonized plants (Figure 2b). In addition, we have analyzed colonized and non-inoculated wheat roots by cell flow cytometry: the number of cells with higher content of DNA increase up to 40% in inoculated roots, indicating a higher proportion of cells in the S-phase (Figure 2c).

**Figure 3:**
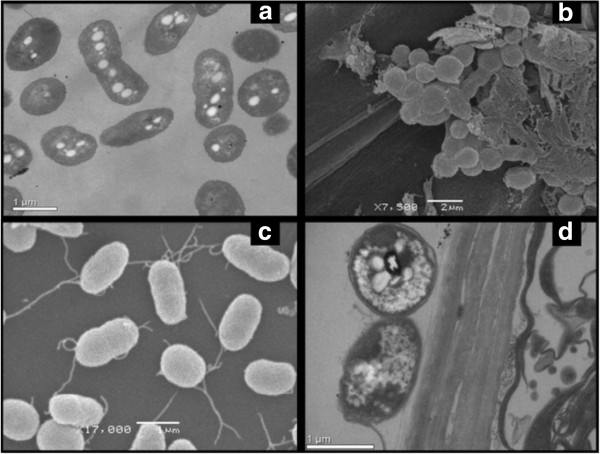
**Electron micrographs of** ***Azospirillum brasilense*** **strain FP2 on wheat roots and electron micrographs of FP2 in liquid culture. (a)** transmission electron micrographs of FP2 grown in liquid culture; **(b)** scanning electron micrographs of biofilm structure on wheat roots; **(c)** scanning electron micrographs of FP2 growing in liquid culture; **(d)** transmission electron micrograph of FP2 adhered to wheat roots.

Bacterial adhesion and biofilm formation on wheat roots were visualized by scanning electron microscopy (Figure 3). Electron micrographs showed abundant adhering material, which might be composed of a matrix of extracellular polysaccharides (Figure 3b). *A. brasilense* anchored on wheat roots by extracellular-polysaccharide rich material was reported previously [13]. In addition, *A. brasilense* adhered to wheat roots visualized by transmission electron microscopy contained a high amount of granules of poly-hydroxy-alkanoate (PHA) (Figure 3d). PHAs are important during periods of carbon and energy starvation, and in *A. brasilense* the accumulation of these reserve materials was reported to support chemotaxis, motility and cell multiplication [14].

### RNA-seq transcriptional profiling

**Figure 4:**
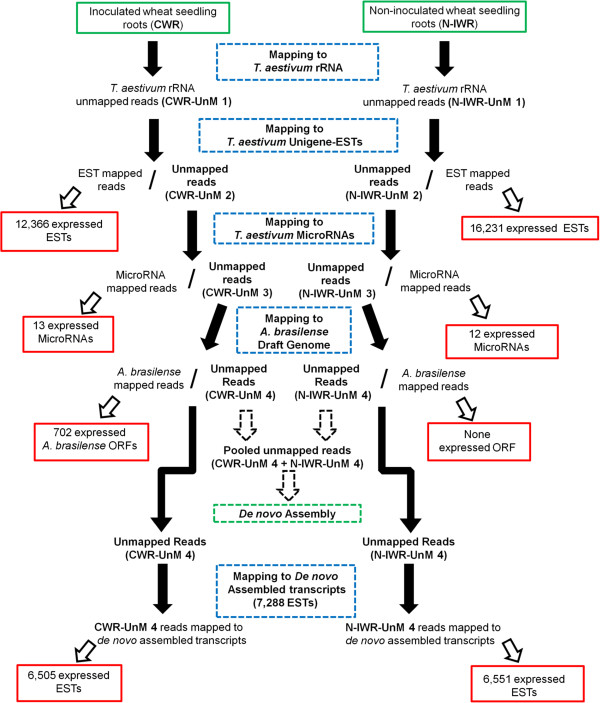
**Mapping strategy.**

The RNA-seq transcriptional analysis was carried out in two independent samples (biological replicates) of each treatment (colonized or non-inoculated), yielding a total of 4 sequencing libraries of seedling roots, which were designated CWR1 and CWR2 libraries (colonized roots) and N-IWR1 and N-IWR2 (non-inoculated roots) (Additional file 1: Figure S1). Each sample was made up of roots of 10 seedlings (~100 mg). Two sequencing runs of each CWR and N-IWR cDNA libraries generated 306 million 50 bp reads yielding more than 15 gigabase of sequenced data. The total of sequenced reads was first mapped to the *T. aestivum* ribosomal RNA to clean-up the sequence data from wheat rRNA (Figure 4). Approximately 9 gigabase of rRNA-free sequence data was then mapped to three reference datasets: 1) UniGene-EST of *T. aestivum*; 2) MicroRNAs-databank of *T. aestivum*; and 3) *A. brasilense* FP2 draft genome. A total of 23,215 expressed sequences from wheat roots and 228 expressed sequences from *A. brasilense,* both with 3-fold or higher coverage, were then quantitatively analyzed.

### RNA-seq transcriptional profiling: mapping strategy

After the ribosomal RNA removal, the analyses of the wheat roots transcriptome profiling involved 3 main-steps (Figure 4): 1) sequential mapping of reads to different reference datasets (UniGene-EST of *T. aestivum*, MicroRNAs-databank of *T. aestivum* and *A. brasilense* genome sequence); 2) *de novo* assembly of unmapped reads; and 3) mapping of unmapped reads to *de novo* assembled ESTs. Using this strategy, we were able to improve the number of mapped reads by 2.5-fold (data not shown). The removal of reads mapping to *T. aestivum* ribosomal RNA sequences from the CWR and N-IWR libraries (Figure 4) was necessary to avoid expression bias, since in a first mapping trial using as reference UniGene-EST a high number of ESTs/rRNA chimeras was found (data not shown). The rRNA-free sequences of CWR and N-IWR (CWR-UnM 1 and N-IWR-UnM 1, respectively) were then mapped to *T. aestivum* UniGene-ESTs data sequence. A total of 16,645 ESTs of wheat was expressed, 12,366 in the CWR libraries and 16,231 in N-IWR libraries (Additional file 2: Table S1A, S1B and Additional file 3: Table S2).

The UniGene-unmapped sequences (CWR-UnM 2 and N-IWR-UnM 2) were then mapped to the microRNAs-dataset of *T. aestivum*. Among the 85 described wheat microRNAs [15] fifteen were expressed, 13 in CWR- and 12 in N-IWR-libraries (Additional file 4: Table S3). The microRNAs-unmapped sequences (CWR-UnM 3 and N-IWR-UnM 3) were subsequently mapped to the *A. brasilense* genome sequence strain Sp245 [16] and to a draft genome of the FP2 strain (unpublished data). Two hundred and twenty eight genes of the *A. brasilense* FP2 strain were identified in the CWR libraries with 3× or higher coverage and none in the N-IWR libraries. Furthermore, when a less stringent coverage cut-off (1×) was applied, 702 *A. brasilense* transcripts were identified in the CWR libraries and no expressed ORF was found in the N-IWR libraries (Figure 4).

Finally, the unmapped reads, named CWR-UnM 4 and N-IWR-UnM 4, which represent reads that did not align to any of the reference sequence datasets, were used to assemble the transcripts *de novo*. A total of 7,288 contigs were assembled and used as reference to map the reads of the CWR-UnM 4 and N-IWR-UnM 4 libraries (Figure 4). Of the 7,288 assembled contigs, 6,570 had enough mapped reads (3× or higher coverage) to be considered expressed; 6,505 in the CWR libraries and 6,551 in the N-IWR libraries (Figure 4).

### RNA-seq biological variability

Biological variability was checked by Pearson correlation coefficients comparing DEseq-normalized expression values within the biological replicates. Correlation coefficients for CWR libraries were 0.93, 0.99, 0.99 and 0.96 (UniGene, microRNAs, *A. brasilense*, *de novo* assembled transcripts, respectively), whereas biological replicates of N-IWR libraries had correlation coefficients of 0.92, 0.97 and 0.81 (UniGene, microRNAs and *de novo* assembled transcripts, respectively). Correlation coefficient for *A. brasilense*-mapped reads was not calculated for N-IWR libraries since no expressed ORF was found in these libraries. Overall, data reproducibility in this study was high and, except for N-IWR mapped to *de novo* assembled transcripts, all pair correlation coefficients were ≥ 0.92.

### Comparison of *Triticum aestivum* RNA-seq profiles: CWR vs N-IWR

Gene expression levels in the CWR (inoculated with *A. brasilense*) libraries are expressed as fold-differences in relation to the expression levels in the N-IWR (non-inoculated) libraries. In the following sections, when we attribute effects on gene expression to *A. brasilense* colonization, we are comparing CWR libraries with N-IWR libraries.

Within the 23,215 expressed-ESTs (16,645 UniGene-mapped and 6,570 from the assembled transcripts) *A. brasilense* colonization caused changes in the expression of 776 ESTs (fold-change ≥2 and *p-value* < 0.05), hereafter named as regulated-ESTs. Of these, 313 ESTs were up-regulated and 463 ESTs were down-regulated (Additional file 5: Table S4).

**Figure 5:**
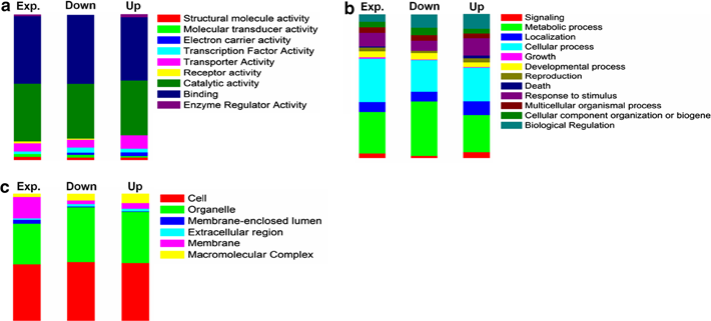
**Gene ontology analysis.** Expressed and differentially expressed wheat genes were classified in the three main gene ontology categories: **(a)** Molecular Functions; **(b)** Biological Processes; and **(c)** Cellular Components. Exp: expressed; Down: down-regulated in colonized roots; Up: up-regulated in colonized roots.

To gain insight into the putative processes involved in the plant-bacterial cross-talk a gene ontology (GO) analysis of the three GO categories - biological processes (BP), molecular functions (MF) and cellular components (CC) - was carried out (Figure 5). To determine whether any of the categories were overrepresented in the group of regulated-ESTs we compared the percentage of each GO category in the sub-set of regulated-ESTs to the percentage of same GO category in the sub-set of expressed-ESTs, and values higher than 1.5-fold were considered overrepresented in the subset of regulated-ESTs. The overrepresented categories in the subset of up-regulated ESTs included: 1) transport activity and enzyme regulator activity (MF); 2) biological regulation and death (BP); and 3) macromolecular complex and extracellular region (CC). While in the down-regulated sub-set these categories comprised: 1) transcription factor activity (MF); 2) biological regulation (BP); 3) macromolecular complex and extracellular region (CC) (Figure 5 and Additional file 6: Table S5A, S5B).

### mRNA levels by quantitative reverse transcription PCR (RT-qPCR)

Quantitative reverse transcription PCR was used to confirm the differential expression of ten selected genes from *T. aestivum* (Additional file 7: Figure S2) and four from *A. brasilense* (Table 1). In all these genes, there were a very good correlation between RNA-seq and RT-qPCR (Pearson coefficient correlation between RNA-seq and RT-qPCR to *T. aestivum* > 0.97, *p-value* < 0.0001). *T. aestivum* gene expression was normalized according to transcript levels of *pp2ac5*, *ubccE2* and a beta-3-tubulin transcript and *A. brasilense* genes according to *rpoC* transcript levels. The selected genes comprised both sub-sets of regulated ESTs (i.e., up- and down-regulated ESTs).

**Table 1:** **RT-qPCR validation:** ***A. brasilense*** **on wheat roots vs. free living** ***A. brasilense*** ^**a**^

Genes	RT-qPCR log2Fold-change
*nifH*	4.6 ± 0.16^*^
*sbpA*	7.5 ± 0.23^***^
*narL-like*	6.4 ± 0.01^***^
*trpB*	−2.3 ± 0.16 ^**^

^a^ *A. brasilense* grown microaerophilic on NFbHP-lactate containing 20 mM NH_4_Cl.

Significant differences in the RT-qPCR (*p < 0.05, **p < 0.01, and ***p < 0.001) were determined using one-tailed t-test.

### Identification of differentially expressed microRNA of *Triticum aestivum* inoculated with *Azospirillum brasilense*

Fifteen wheat microRNAs were found in the RNASeq data, and one of them, tae-MIR444, was up-regulated in the colonized roots (8.6-fold, *p-value* 0.019) (Additional file 4: Table S3). MIR444 and its predicted target, MADS box transcriptional factors, are conserved in monocots. MADS box transcriptional factors play key roles in controlling floral organ development in cereals during vernalization (extended exposure to low temperature that induces plants from temperate regions to flower) [17, 18]. One other possible target for MIR444 is an homolog of *A. thaliana* regulatory factor ANR1, which is a MADS box transcriptional factor that promotes lateral roots elongation in response to nitrate supply [19, 20]. Seventeen ESTs encoding MADS box transcriptional factors were expressed in the wheat roots, but no significant change in expression was noted when we compared CWR to N-IWR libraries (Additional file 8: Table S6). Further investigation is required to determine the possible role of MIR444 up-regulation in colonized roots.

### *Azospirillum brasilense* expressed transcripts in wheat roots

Of the 702 expressed genes of *A. brasilense* those related to bacterial adhesion, adaptation processes and nitrogen fixation were the most recurrent (Additional file 9: Table S7). We identified several genes encoding proteins related to the initial steps of plant-bacterial interaction (chemotaxis, adhesion and biofilm formation). The *sbpA* gene*,* which encodes an acidic *A. brasilense* protein induced by root exudates [21], was highly expressed in the CWR-libraries. RT-qPCR analyses confirmed expression of *sbpA* in *A. brasilense* colonizing wheat roots (Additional file 9: Table S7 and Table 1). The protein SbpA is required for chemotaxis towards sugars (e.g., D-galactose, L-arabinose and D-fucose) and is involved in the uptake of D-galactose [22]. The transcriptional regulator GbpR and the ABC sugar transporters GguA and GguB were also highly expressed in the CWR-libraries. Additionally, genes encoding: monosaccharides transporters, and proteins related to polysaccharides, exo-polysaccharides and lipopolysaccharides biosynthesis and transport were also highly expressed in the CWR-libraries (Additional file 9: Table S7). Moreover, three genes of calcium-binding proteins (two of them being hemolysin-type), which have been associated with *Rhizobium* adhesion on host roots [23], were expressed in CWR-libraries. Taken together these results suggest that the time point of the colonization picked to perform the RNA-seq experiments (i.e., three days of colonization) corresponds to the initial steps of plant-bacterial interaction, when chemotaxis, adhesion and biofilm formation are prominent processes.

Interestingly, a *narL*-like gene was highly expressed in CWR-libraries, a result confirmed by RT-qPCR analyses (Additional file 9: Table S7 and Table 1). NarX/NarL is a classical two-component system of membrane sensor protein (NarX) and DNA-binding regulator (NarL) that regulates the respiratory membrane-bound nitrate reductases in *E.coli* and *P. aeruginosa*[24, 25]. In addition, among the expressed genes we found seven ORFs involved in production of poly-β-hydroxy-alkanoates (PHAs) (PHB polymerases, poly(3-hydroxyalkanoate) synthetase and phasins). This result is consistent with the finding of high amounts of intracellular PHAs in *A. brasilense* cells colonizing wheat root surface (Figure 3d). Finally, expression of *A. brasilense* superoxide dismutase (SodB) (Additional file 9: Table S7) indicates activation of oxidative stress protection systems of *A. brasilense* against plant-defense oxidative burst.

*A. brasilense* is able to convert atmospheric nitrogen into ammonium through the action of the nitrogenase complex under appropriate conditions (e.g., microaerobically and low nitrogen levels) [26] and it may transfer fixed nitrogen to the associated plant. The relative high expression of nitrogenase complex genes (*nifHDK* operon), which was also confirmed by RT-qPCR analyses (Table 1), suggest that *A. brasilense* adhered to wheat roots is fixing nitrogen. Expression of nitrogenase complex on wheat roots has been shown using *nifH:lacZ* fusion [27, 28].

### Differentially expressed wheat genes associated to plant-microbe interaction

First, we focused our analysis on the screening of the host response usually found at some stage of plant-microbe interaction. The wheat ESTs encoding host response proteins were grouped into three sets: 1) defense mechanism; 2) hormone imbalances and 3) secretion of phytochemicals.

#### Defense mechanism

We looked at the wide variety of inducible defense mechanisms (e.g., oxidative burst, production of antimicrobial compounds and expression of defense-related genes [29, 30]) usually triggered upon microbe recognition. Hundreds of defense-related ESTs (R-genes-encode resistance-proteins e.g., kinases with leucine rich repeat receptors (LRR-kinases), endoglucanases, disease resistance proteins, etc.) were found among the expressed ESTs (data not shown); twenty-eight of those had changes in their expression in response to *A. brasilense* colonization (Additional file 10: Table S8). Changes in expression of R-genes were found in both sub-sets of down-regulated (12 ESTs) and up-regulated ESTs (16 ESTs). In addition, ten heat shock proteins (Hsp), which play roles in plant response defense and are also required to fold LRR-receptors in a signal competent state [31, 32] were found in sub-sets of up-regulated ESTs (Additional file 10: Table S8).

#### Hormone imbalances in colonized plants

The phytohormones auxins and ethylene have been intensely studied in plant growth and development. Three ESTs encoding auxin-induced proteins changed their expression in response to *A. brasilense* colonization (Additional file 10: Table S8). The first was a transcriptional factor ETTIN/ARF3, which mediates auxin dependent flower and fruit development by binding on auxin receptor (AuxRec) (3.6-fold, down-regulated). An aldo-keto-reductase auxin-induced was also down-regulated (2-fold) while a calmodulin-dependent auxin-induced protein SAUR (small auxin up RNA), whose response is mediated by calcium, was up-regulated (4.3-fold). In addition, three calmodulin-like proteins were down-regulated by *A. brasilense* colonization (Additional file 10: Table S8). Additional investigations are required, including gene expression of aerial parts of wheat, to bring insights into the pattern of auxin-related gene expression in response to *A. brasilense* colonization.

*A. brasilense* colonization promoted an interesting decrease of expression of *ACO*, which encodes for ACC oxidase (3.1-fold) (Additional file 10: Table S8). ACC oxidase catalyzes the conversion of 1-aminocyclopropane-1-carboxylate (ACC) to ethylene. Although ethylene production regulation is most related to ACC synthase expression, ACC oxidase transcription also contributes to regulation of ethylene production [33]. This result suggests a decreased amount of ethylene production in inoculated wheat roots. Thus, *A. brasilense* colonization might suppress the inhibition of root cell elongation promoted by ethylene, reflected in the improvement of root systems of colonized plants.

#### Secretion of phytochemicals

Three ESTs encoding enzymes related to flavonoids biosynthesis showed expression decreased in response to *A. brasilense* colonization: flavonol 3-sulfotransferase and two anthocyanidin -o-glucosyltransferases (Additional file 10: Table S8). Flavonol 3-sulfotransferase catalyzes flavonoid sulfation, and flavonoid sulfates are supposed to be involved in detoxification of active hydroxyl groups and in sequestering of sulfate groups of plants growing under saline conditions. Interestingly, anthocyanin biosynthesis is activated by nitrogen deficiency, while nitrogen compounds (e.g., fertilizers) repress the flavonoid biosynthesis [34, 35], raising the possibility of improvement of nitrogen nutrition of the colonized wheat roots.

### Wheat response to *Azospirillum brasilense* colonization

*A. brasilense* colonization caused changes in the expression of 776 ESTs ranging from transporters, heat shock proteins, helicases, resistance-proteins (R-genes), to cell cycle control.

Close to five hundred ESTs encoding transporters were expressed by wheat roots; among these, 9 were up-regulated and 3 down-regulated (Additional file 11: Table S9). In addition, 559 more EST encoding proteins, which were grouped in transport activity by GO analysis, were expressed in wheat roots (10 up- and 11 down-regulated) (Additional file 12: Table S10). Interestingly, genes encoding a nitrate efflux transmembrane transporter (homolog of *A. thaliana NAXT)* and an oligopeptide transporter (*PTR2*) were 4.2- and 2.6-fold, respectively, up-regulated, whilst another twenty three nitrate transporters showed no significant change in expression (Additional file 11: Table S9). However, we did not find any significant change in nitrogen content (including nitrate) in the hydroponic medium 3 days after wheat roots inoculation (data not shown). On the other hand, two ESTs encoding a cytosolic form of glutamine synthetase (GS1) were up-regulated (3.1- and 2.3-fold) (Additional file 11: Table S9). GS activity composes the major route for inorganic N incorporation into organic molecules [3]. GS1 expression was also shown to be up-regulated in sugar cane seedlings colonized by the PGPB *Gluconacetobacter diazotrophicus* and *Herbaspirillum rubrisubalbicans*[36].

Twenty-two ESTs encoding proteins related to cell cycle regulation and improvement of root growth showed changes in their expression responding to *A. brasilense* colonization, 16 in the sub-set of up-regulated and 8 in the down-regulated ESTs (Additional file 13: Table S11). The higher number of up-regulated ESTs of this class agreed with the flow cytometry results (Figure 2c) that showed an increased proportion of cells in the S-phase, which indicate higher rates of DNA replication in colonized wheat roots.

## Discussion

### Simultaneous RNA-Seq analysis of wheat roots and Azospirillum brasilense

To our knowledge, this is the first report of a dual RNA-Seq transcriptional analysis of plant-PGPB interaction (simultaneous analysis of changes in expression of both host and bacteria). There are only few studies with the application of high-throughput techniques (e.g., RNA-Seq) in plant-PGPB interactions [6], including those focused either on the host or the PGPB. On the other hand, investigation of host-pathogen interactions by dual RNA-Seq (HeLa cell and vaccinia virus [37]; bone marrow-derived dendritic cells from *Mus musculus* and the pathogen fungus *Candida albicans*[38] and *Oryza sativa* and *Magnaporthe oryzae* (blast fungus) [10]) has become feasible and expected to become the gold standard in the studies of host-microbe interactions [9]. Further dual RNA-Seq quantitative studies of plant-PGPB interaction should be undertaken in order to improve knowledge of the molecular basis of plant benefits by PGPB.

Although the common wheat (*Triticum aestivum*) is one of the most important food crops in the world, the complete assembly of its genome sequence is not available. Large genome (16 Gb), high amount of repetitive sequences (≈90%) and hexaploid nature make the complete assembling of wheat genome a very hard task to be accomplished. Luckily, RNA-Seq transcriptional profiling is a quite practical alternative to assess the functional genome of non-model organisms with no defined genome reference.

Several wheat transcriptional studies have successfully applied RNA-Seq analyses to investigate wheat transcriptomes applying two main strategies: 1) assembling of sequenced reads into contigs (e.g., homoeolog-specific assembly of wheat transcriptome [39], optimization of *de novo* assembly of wheat transcriptome [40], assembly of sequence reads from three bread wheat varieties to identify SNPs candidates [41], assembly of sequence reads into contigs to investigate the transcription factor GPC (grain protein content) during monocarpic senescence [42], and assembly of transcripts to investigate polyploidization events in common wheat [43]); and 2) mapping the sequenced reads to a reference of wheat ESTs or to a genome of a correlated organism such as rice (*Oryza sativa*) (e.g., transcriptome of starchy endosperm of the developing wheat seeds, at different times of aleurone layer development [44], transcriptome analysis of the developing starchy endosperm [45], mapping of sequence reads into the unigene sequences from two lines of wheat to identify SNPs [46] and mRNA tag analysis to investigate cellular and metabolic responses of wheat seedlings triggered by H_2_O_2_[47]).

Here, we used a mixed strategy, by mapping the reads to wheat-ESTs and assembling of unmapped sequence reads. This strategy allowed for an improvement in the number of quantitatively analyzed genes.

### A possible role of NarX/NarL-like operon on *Azospirillum*-wheat root adhesion

The NarX/NarL operon regulates the respiratory membrane-bound nitrate reductases in *E.coli* and *P. aeruginosa*[24, 25]. Nitrate binds to the P box element (a highly conserved nitrate recognition region of NarX) altering NarX conformation, allowing its auto-phosphorylation and subsequent phosphorylation of NarL, which in turn binds to specific DNA target sites resulting in activation or repression of target operon transcription [24, 25]. In *E. coli* expression of the *narXnarL* operon is activated by Fnr proteins under limitation of oxygen and by NarL in the presence of nitrate [24]. Interestingly, in *P. aeuruginosa* NarX/NarL plays an essential role in the biofilm through the activation of the motility regulon that controls the biofilm dispersion [25]. The NarL-like protein of *A. brasilense* expressed during colonization may play a similar role in adhesion to the root surface.

### *Triticum aestivum* expression profile modulated by *Azospirillum brasilense* colonization

#### Nitrate transporters

Plants respond to nitrate, which is the major source of mineral nitrogen for higher plants, improving its up-take and metabolism. The most prominent effect of nitrate supply is the stimulation of lateral root formation [20]. Genes encoding a nitrate efflux transmembrane transporter (*NAXT*) and an oligopeptide transporter of the NRT1(PTR) family (*PTR2*) were up-regulated in colonized roots. Nitrate efflux was reported in response to stress-generated pH acidification in *A. thaliana* growing in hydroponic medium [48]. Nitrate efflux was also reported as an early signaling mechanism in tobacco plants as a response defense against crytogein, a proteinaceous elicitor from a virulent tobacco pathogen (*Phytophthora cryptogea*) [49]. Additional investigations are required to determine a possible role of nitrate efflux transmembrane transporter up-regulation in colonized roots.

#### Phytochemicals and phytohormones

It is well known that secretion of phytochemicals is a critical step in plant-microbe interaction [50–52]. For example, plant root exudates containing flavonoids are recognized by *Rhizobium* and play a critical role in the legume-rhizobium symbiosis [51, 52]. In addition, plant hormones (phytohormones) play important roles in response to biotic stress, e.g., bacterial colonization. Besides their significant role in response to biotic stress, phytohormones also play essential roles in the regulation of plant growth and development [30, 53, 54]. Abiotic and biotic stresses challenge plant tissues triggering ethylene production, which promotes, among other effects, inhibition of plant root cell elongation [33]. The ACC oxidase (ACO) expression was down-regulated in colonized roots. Since *ACO* transcription contributes to regulation of ethylene production, decreasing *ACO* expression reflects in lower amount of ethylene in wheat roots, avoiding ethylene inhibition effects in root elongation of colonized wheat seedlings [33]. ACC oxidase expression also can be used as an indicator of osmotic stress (drought or saline stresses). Pepper plants (*Capsicum annuum* L.) inoculated with PGPB (*Bacillus* sp. and *Arthrobacter* sp.) presented a significant decrease in *ACO* expression when compared with the non-inoculated plants under osmotic stress promoted by PEG (polyethylene glycol) treatment. The decrease of *ACO* expression might contribute to the relief of osmotic stress by these PGPB [55]. A decrease in endogenous ethylene levels also plays a role in bacterial-induced salinity tolerance [56].

**Figure 6:**
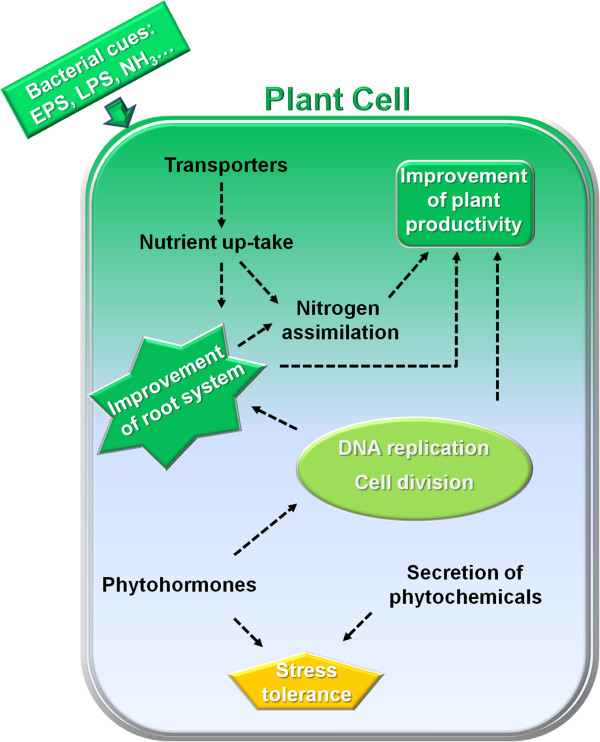
**Proposed effects of** ***Azospirillum brasilense*** **inoculation.**

Plants growing under saline conditions produce flavonoid sulfates, which might be implicated in detoxification of active hydroxyl groups and in the sequestering of sulfates groups [57]. Even though wheat seedlings were not under a typical saline stress condition, the gene that encodes a flavonol 3-sulfotransferase, which catalyzes flavonoid sulfation, was down-regulated in colonized roots, suggesting that colonized plants are more resistant to saline stress. Taken together, these results suggest that *A. brasilense* colonization besides promoting enhancement of the root system may play a role in plant stress tolerance, an effect previously observed in other studies (Figure 6) [56, 58–64].

### Improvement of nutrient up-take in inoculated wheat roots

In addition to several reports of increases in root biomass promoted by *Azospirillum* inoculation [6, 11, 12], here we present evidence that *A. brasilense* colonization improve nutrient acquisition by not only increasing the root surface area, but also by regulating the expression of nutrient transporters (Figure 6). Supporting this hypothesis, our results suggest: 1) enhancement of root surface area followed by increased DNA synthesis in root cells and up-regulation of ESTs encoding cell cycle regulators; 2) repression of *ACO* expression that might reflect in lower ethylene production; and 3) up-regulation of transporters ranging from metal- to oligopeptides-transporters.

Additionally, EST encoding proteins related to anthocyanin biosynthesis, which is usually repressed by increased amount of nitrogen content [34, 35], were down-regulated in CWR-libraries suggesting higher amount of nitrogen content in the colonized plants. Finally, the up-regulation of wheat glutamine synthetase and of *A. brasilense* nitrogenase strongly suggests that the PGPB colonization improves nitrogen nutrition of wheat plants.

## Conclusions

Simultaneous RNA-Seq of plant roots colonized by *A. brasilense* showed a remarkable change in expression of plant genes involved in transport activity, reflected in a direct effect on the up-take of nutrients, such as nitrogen. Additionally, genes of categories related to DNA replication and cell division were also responsive to the presence of the bacteria. These changes in gene expression are likely correlated with improvement in growth of wheat seedling colonized by *A. brasilense* under axenic conditions. The results reinforce the use of PGPB as an alternative to improve nutrient acquisition in important crops such as wheat. Finally, genetic manipulation of the differentially expressed wheat genes may lead to the development of new cultivars with improved productivity traits.

## Methods

### Plant growth and bacterial colonization

*T. aestivum* (CD-104 cv.) seeds were surface-sterilized by washing with ethanol 70% for 0.5 min, followed by shaking (120 rpm) for 5 min with acid hypochlorite, washing 3-times with ultrapure sterilized water and 4 h incubation with ultrapure sterilized water at room temperature. The seeds were then incubated in a rotary shaker (120 rpm) for 5 min with acid hypochlorite [0.5% (v/v) NaOCl, 0.18% (v/v) concentrated HCl, 0.01% (v/v) Tween 80 and 7 mM KH_2_PO_4_] at room temperature, washed 3-times with ultrapure sterilized water, followed by a 5 min incubation with H_2_O_2_ 35% (v/v). Finally, the seeds were washed 4-times with ultrapure sterilized water and then germinated in water agar plates at 30°C for 12 h under darkness. Germinated seedlings were transferred to sterile glass tubes containing 25 mL Hoagland’s nutrient solution [65] (two seedlings per tube) and cultivated at 26°C under 14 h light/10 h darkness for 24 h. Each tube was then inoculated with 0.25 mL of *A. brasilense* FP2 suspension. Bacterial suspension was prepared by growing *A. brasilense* FP2 in a 60-mL flask containing 10 mL NFbHP-lactate medium [66] supplemented with 20 mM of NH_4_Cl at 30°C and 120 rpm in an orbital shaker to an optical density (600 nm) of 1.0. The culture was then centrifuged and the pellet was re-suspended in 100 mL Hoagland’s nutrient solution (without nitrogen or carbon) to approximately 1.5 × 10^7^ CFU/mL. The inoculated seedlings were incubated for 3 days at 26°C under 14 h light/10 h darkness. Non-inoculated control seedlings were cultivated exactly as described except that 0.25 mL of sterile Hoagland’s nutrient solution was added.

### *Azospirillum brasilense* cells count

Colonized *T. aestivum* seedling roots were weighed, macerated in sterile saline solution, diluted, and plated on NFbHP-lactate agar containing 20 mM NH_4_Cl. Bacterial populations were expressed as CFU/g of fresh roots. Bacterial counts were also performed with surface-sterilized roots (1 min in 70% ethanol, followed by 1 min in 1% chloramine T (Sigma) and rinsed 3 times with sterile water) in order to check whether the *A .brasilense* FP2 strain was able of endophytic colonization. Bacterial cells were also counted in the Hoaglands solution bathing the roots and expressed as CFU/mL.

### Nuclei isolation and flow cytometry

The relative DNA content was analysed essentially as described by [67]. Shortly, *T. aestivum* roots were collected three-days after inoculation and sliced with a single-edge razor blade in a glass petri dish containing nuclei isolation buffer (45 mM MgCl_2_, 20 mM MOPS, 30 mM sodium citrate, 0.1% Triton X-100, pH 7.0) [67]. Nuclei (in 1.5 mL buffer) were passed through a filter of pore size 40 μm, stained with 50 μg/mL propidium iodide (PI) and analyzed on a FACS Calibur flow cytometer (Becton, Dickinson and Company, USA) with CellQuest software. Side scatter versus forward scatter dot plots were used to locate and gate nuclear populations by particle size. The FL2-A channel was used for detection of PI fluorescence (DNA content). The DNA content of at least 5000 nuclei were analyzed in each sample.

### Transmission electron microscopy and scanning electron microscopy

*T. aestivum* roots were fixed with Karnovsky’s fixative [68], post-fixed with 2% OsO_4_ in 0.1 M cacodylic acid buffer (pH 7.2) for 1 h and embedded in Epon 812 [69]. After contrasting with 2% uranyl acetate [70] and lead citrate [71], samples were examined with a JEOL-JEM 1200 EX II transmission electron microscope. For scanning electron microscopy, *T. aestivum* roots were fixed with Karnovsky’s fixative [68], washed in 0.1 M cacodylic acid buffer (pH 7.2) and dehydrated in ethanol. Critical-point dryness was obtained with a Bal-Tec CPD – 030, the samples were coated with gold using a Balzers SCD – 030 sputter coater and examined with a JEOL-JSM 6360 LV scanning electron microscope.

### RNA-seq profiling experiment

The RNA-seq transcriptional analysis was carried out with two biological replicates of each treatment (colonized and non-inoculated *T. aestivum* roots). Each replicate consisted of roots of ten *T. aestivum* seedlings (five tubes with two seedlings per tube) (Additional file 1: Figure S1). *T. aestivum* roots were washed twice with sterilized water, cut, mixed with RNAlater® (Applied Biosystems, Carlsbad, CA) and stored at −80°C. Total RNA extraction, rRNA depletion, library construction and cDNA sequencing on a Life Technologies’ SOLiD 4® platform were performed according to the manufacturer’s instructions. The libraries were mapped to *T. aestivum* and *A. brasilense* sequence references using CLC Genomics Workbench 4.8, with a tolerance of 2 mismatches, and minimum length fraction of 0.9 to *A. brasilense* and 0.8 to *T. aestivum* and minimum similarity fraction of 0.8 for both. Only uniquely mapped reads were considered in our analysis. The unmapped reads were *de novo* assembled using CLC Genomics Workbench 4.8 [accession number, EMBL: E-MTAB-2301]. DEseq package was used to estimate differential gene expression, performing a negative binomial distribution and a shrinkage estimator for the distribution’s variance and size-factor normalization [72]. The differentially expressed genes were considered to be significant at *p-value* < 0.05 and absolute fold-change ≥ 2-fold. Gene ontology analysis was performed by Blast2go software [73].

### Analysis of gene expression by RT-qPCR

RT-qPCR was carried out with two independent biological replicates (other than those used for RNA-seq experiments) of colonized and non-inoculated *T. aestivum* roots (Additional file 1: Figure S1). Two micrograms of extracted RNA was used to synthesize cDNA with a high-capacity cDNA reverse transcription kit (Applied Biosystems, Carlsbad, CA). Relative expression levels were estimated as described [74]. *T. aestivum* target gene expression was normalized to that of *pp2ac5*, *ubccE2* and a beta-3-tubulin using geNorm 3.4 software [75] and *A. brasilense* target genes to that of *rpoC*. All samples were run in triplicate and significant differences were determined using one-tailed t-test.

## Electronic supplementary material

Additional file 1: Figure S1: RNA-Seq and RT-qPCR experiments design. CWR: colonized wheat roots; N-IWR: non-inoculated wheat roots; each biological replicate was compound by five tubes with two seedlings per tube. (PDF 510 KB)

Additional file 2: Table S1A: RNA-seq: mapping results. **Table S1B.** RNA-seq: expressed and differentially expressed genes. ^a^3× or higher coverage; ^b^1× or higher coverage; ^c^Not determined. (PDF 88 KB)

Additional file 3: Table S2: *Triticum aestivum* Expressed ESTs. ^a^Fold-change in red indicates down-regulation in colonized wheat roots (CWR); (+) ND not expressed in the N-IWR libraries; (−) ND not expressed in the CWR libraries. (XLSX 1 MB)

Additional file 4: Table S3: *Triticum aestivum* expressed micro-RNAs. ^a^Fold-change in red indicates lower level of expression in colonized wheat roots (CWR); ^b^Not considered as expressed micro-RNA. (PDF 15 KB)

Additional file 5: Table S4: Differentially expressed *Triticum aestivum*-ESTs. ^a^Fold-change in red indicates down-regulation in colonized wheat roots (CWR); (+) ND not expressed in the N-IWR libraries; (−) ND not expressed in the CWR libraries. (PDF 386 KB)

Additional file 6: Table S5A: Overrepresented GO categories of up-regulated *Triticum aestivum*-ESTs in colonized roots. **Table S5B.** Overrepresented GO categories of down-regulated *Triticum aestivum*-ESTs in colonized roots. ^a^Fold-change in red indicates down-regulation in colonized wheat roots (CWR). (PDF 116 KB)

Additional file 7: Figure S2: Scatter plot: RNA-seq vs. RT-qPCR. RNA-seq and RT-qPCR relative level of expression are presented as log2 (fold-change). *Triticum aestivum* genes presented in the scatter plot: TaS_37759224**, TaS_52545880**, contig_4027***, TaS_52541981*, TaS_22370925*, TaS_37846208*, TaS_17890143*, TaS_26026646*, TaS552541449** and contig_2766**. RNA-seq data represent biological duplicates of colonized and non-inoculated wheat roots. RT-qPCR data represent biological duplicates of colonized and non-inoculated wheat roots, obtained independently from the RNA-seq samples, and each sample was run in triplicate. Significant differences in the RT-qPCR (*p < 0.05, **p < 0.01, and ***p < 0.001) were determined using one-tailed t-test. Pearson coefficient correlation between RNA-seq and RT-qPCR was > 0.97, p-value < 0.0001. (PDF 8 KB)

Additional file 8: Table S6: *Triticum aestivum* MADs-Box expressed-ESTs. ^a^Fold-change in red indicates lower level of expression in colonized wheat roots (CWR). (PDF 15 KB)

Additional file 9: Table S7: Expressed ORFs of *Azospirillum brasilense* strain FP2 colonizing *Triticum aetivum* seedling roots. (PDF 36 KB)

Additional file 10: Table S8: Host response found during plant-microbe interaction: Defense mechanism, hormone imbalances and secretion of phytochemicals. ^a^Fold-change in red indicates down-regulation in colonized wheat roots (CWR); (+) ND not expressed in the N-IWR libraries; (−) ND not expressed in the CWR libraries. (PDF 30 KB)

Additional file 11: Table S9: ESTs of *Triticum aestivum* encoding transporters. ^a^Fold-change in red indicates lower level of expression in colonized wheat roots (CWR); (+) ND not expressed in the N-IWR libraries; Up-regulated, Down-regulated and Expressed ESTs are shading in red, blue and yellow respectively. (PDF 333 KB)

Additional file 12: Table S10: ESTs of *Triticum aestivum* encoding proteins that were grouped in transport activity by GO analysis. ^a^Fold-change in red indicates lower level of expression in colonized wheat roots (CWR); (+) ND not expressed in the N-IWR libraries; Up-regulated, Down-regulated and Expressed ESTs are shading in red, blue and yellow respectively. (PDF 752 KB)

Additional file 13: Table S11: ESTs of *Triticum aestivum* encoding proteins related to cell cycle regulation*.* ^a^Fold-change in red indicates down-regulation in colonized wheat roots (CWR). (PDF 17 KB)
