# Supplementary material for: Dual RNA-seq transcriptional analysis of wheat roots colonized by Azospirillum brasilense reveals up-regulation of nutrient acquisition and cell cycle genes
Source: BMC Genomics. 2014 May 16;15(1):378. doi: 10.1186/1471-2164-15-378 (PMC4042000; doi:10.1186/1471-2164-15-378)
Supplement: Supplementary file 1 — Additional file 1: Figure S1: RNA-Seq and RT-qPCR experiments design. CWR: colonized wheat roots; N-IWR: non-inoculated wheat roots; each biological replicate was compound by five tubes with two seedlings per tube. (PDF 510 KB) [file 12864_2013_6083_MOESM1_ESM.pdf]

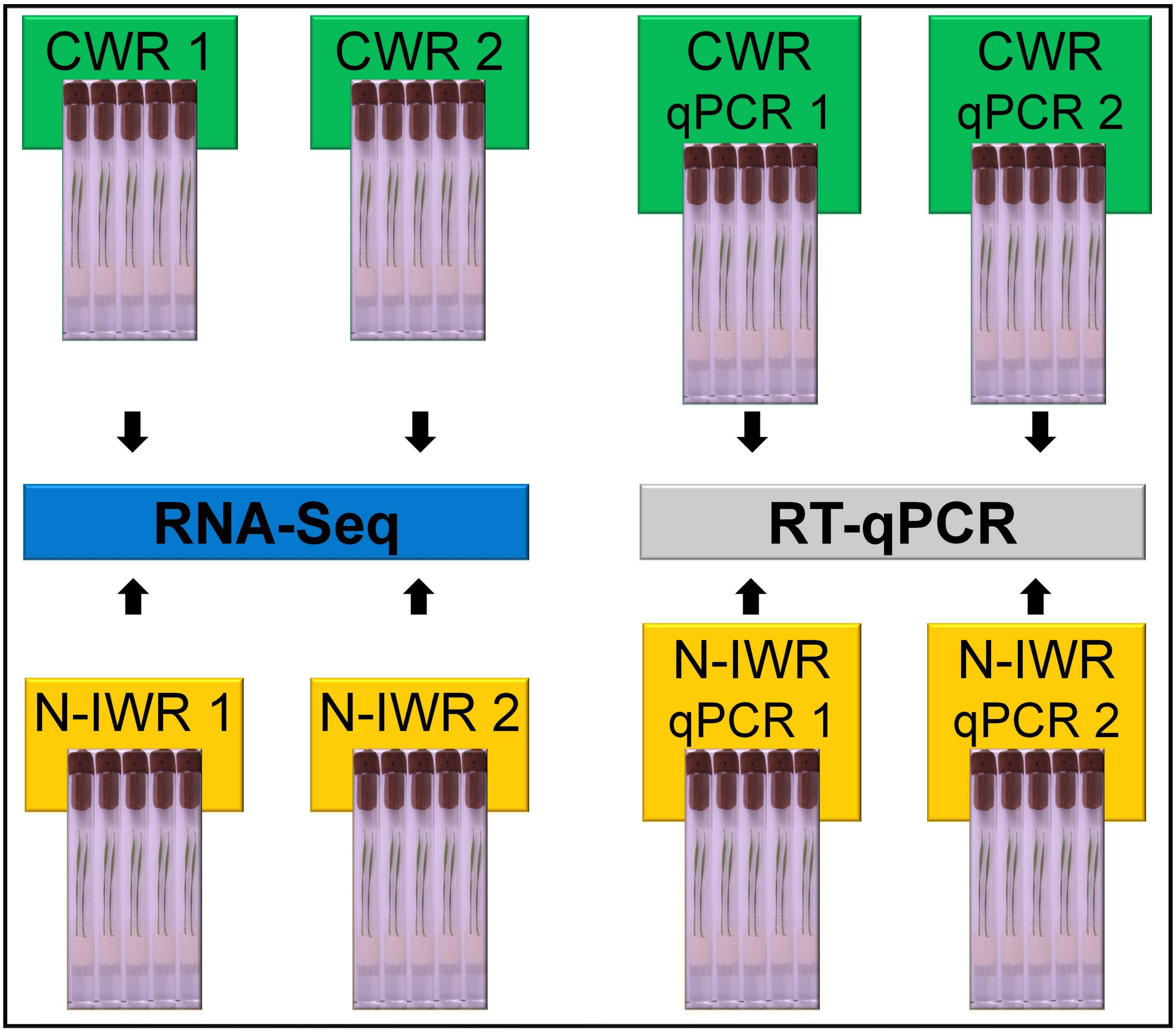

Figure 1S RNA-Seq and RT-qPCR experiments design

CWR: colonized wheat roots; N-IWR: non-inoculated wheat roots; each biological replicate was compound by five tubes with two seedlings per tube.
