# Supplementary material for: Dual RNA-seq transcriptional analysis of wheat roots colonized by Azospirillum brasilense reveals up-regulation of nutrient acquisition and cell cycle genes
Source: BMC Genomics. 2014 May 16;15(1):378. doi: 10.1186/1471-2164-15-378 (PMC4042000; doi:10.1186/1471-2164-15-378)
Supplement: Supplementary file 2 — Additional file 2: Table S1A: RNA-seq: mapping results. Table S1B. RNA-seq: expressed and differentially expressed genes. a3× or higher coverage; b1× or higher coverage; cNot determined. (PDF 88 KB) [file 12864_2013_6083_MOESM2_ESM.pdf]

Table S1A RNA-seq: mapping results

Libraries	Sequenced reads	rRNA-free reads	UniGenes mapped reads	*T. aestivum*-microRNA mapped reads	*A. brasilense* mapped reads	Assembled-EST mapped reads
**CWR**	134,846,641	75,789,954	9,441,999	7,292	106,285	8,528,773
**N-IWR**	171,187,552	103,071,790	14,279,346	11,253	6,214	11,124,992
**Total**	306,034193	178,861,744	23,721,345	18,545	112,499	19,653,765

Table S1B RNA-seq: expressed and differentially expressed genes

Libraries	UniGene-EST	*T. aestivum* microRNA	Assembled-EST	Total of *T. aestivum* EST	*A. brasilense*
**Expressed**	16,645	15	6,570	23,215	228[a] / 702[b]
**Down-regulated**	304	0	159	463	ND[c]
**Up-regulated**	186	1	127	313	ND[c]

[a] 3X or higher coverage; [b] 1X or higher coverage; [c] Not determined.
