# Supplementary material for: Dual RNA-seq transcriptional analysis of wheat roots colonized by Azospirillum brasilense reveals up-regulation of nutrient acquisition and cell cycle genes
Source: BMC Genomics. 2014 May 16;15(1):378. doi: 10.1186/1471-2164-15-378 (PMC4042000; doi:10.1186/1471-2164-15-378)
Supplement: Supplementary file 4 — Additional file 4: Table S3: Triticum aestivum expressed micro-RNAs. aFold-change in red indicates lower level of expression in colonized wheat roots (CWR); bNot considered as expressed micro-RNA. (PDF 15 KB) [file 12864_2013_6083_MOESM4_ESM.pdf]

Table S3 *Triticum aestivum* expressed micro-RNAs

Micro-RNA ID	Fold Change [a]	*p-value*	N-IWR coverage	CWR coverage
tae-MIR444	8.6	0.0195	1.3[b]	7.0
tae-MIR1121	5.7	0.3286	1.5 [b]	4.1
tae-MIR1137	2.5	0.2219	1.7 [b]	3.7
tae-MIR1122	1.8	0.2356	12.5	17.1
tae-MIR2030	1.5	0.5086	10.8	11.5
tae-MIR2009c	1.3	0.9223	14.4	11.1
tae-MIR160	1.2	0.7034	1028.0	837.2
tae-MIR2014	1.1	0.9222	473.5	353.1
tae-MIR1120	2.4	0.7040	6.3	2.5[b]
tae-MIR1139	2.2	0.2681	6.9	2.3[b]
tae-MIR1119	2.2	0.2865	56.2	21.5
tae-MIR1126	1.8	0.6350	93.0	36.9
tae-MIR1138	1.5	0.2464	9.8	3.4
tae-MIR2028	1.3	0.1127	1931.8	1065.7
tae-MIR1134	1.3	0.8766	10.8	6.9

[a] Fold-change in red indicates lower level of expression in the colonized wheat roots (CWR); [b] Not considered as expressed micro-RNA.
