# Supplementary material for: Dual RNA-seq transcriptional analysis of wheat roots colonized by Azospirillum brasilense reveals up-regulation of nutrient acquisition and cell cycle genes
Source: BMC Genomics. 2014 May 16;15(1):378. doi: 10.1186/1471-2164-15-378 (PMC4042000; doi:10.1186/1471-2164-15-378)
Supplement: Supplementary file 5 — Additional file 5: Table S4: Differentially expressed Triticum aestivum-ESTs. aFold-change in red indicates down-regulation in colonized wheat roots (CWR); (+) ND not expressed in the N-IWR libraries; (−) ND not expressed in the CWR libraries. (PDF 386 KB) [file 12864_2013_6083_MOESM5_ESM.pdf]

Table S4 Differentially expressed *Triticum aestivum*-ESTs

Sequence ID	Fold Change[a]	*p-value*	Sequence description
Ta_S58847638	(+)ND	0.003693	Hypothetical protein osi_06374 [Oryza sativa Indica Group]
Ta_S58840665	(+)ND	0.044869	Hypothetical protein osj_33218 [Oryza sativa Japonica Group]
Ta_S58861971	(+)ND	0.030767	Protein
contig_119	(+)ND	2.56E-07	Not found
Ta_S58868582	(+)ND	0.000289	Not found
Ta_S37855427	(+)ND	0.002587	Not found
Ta_S32661173	(+)ND	0.037164	Not found
Ta_S58882496	951.2	9.05E-23	Not found
contig_160	765.2	6.84E-21	Not found
contig_3469	645.0	2.00E-32	Transcriptional regulator
Ta_S58859374	571.2	0.004452	Not found
Ta_S37940839	505.1	0.005203	Pre-mrna processing protein expressed
contig_4627	389.5	2.60E-16	Not found
Ta_S58863092	340.5	0.003014	Alpha-l-fucosidase 2 precursor
contig_2198	123.6	0.015971	Not found
Ta_S52544833	80.0	0.037528	Small heat shock protein
Ta_S16236735	76.4	0.019596	Heat-shock protein
Ta_S58854607	59.8	0.001188	Replication factor c subunit
Ta_S16229254	48.9	0.04891	Kda class iv heat shock protein
Ta_S52547097	46.7	0.028523	Probable serine threonine-protein kinase at1g54610-like
Ta_S58853475	39.9	4.51E-05	Not found
contig_5353	34.0	5.87E-10	Not found
Ta_S52541981	29.4	0.019756	Kda class iii heat shock
Ta_S58886208	23.3	0.010146	Not found
contig_2820	22.2	0.038959	Not found
Ta_S58858619	19.9	0.029221	Low molecular weight heat shock protein
Ta_S12923094	19.5	0.025603	Not found
Ta_S58850802	18.8	9.18E-05	Not found
Ta_S52541158	18.5	0.020768	PREDICTED: uncharacterized protein LOC100846009 [Brachypodium distachyon]

contig_2853	16.8	0.0424	Not found
Ta_S58884106	14.9	0.000358	Af114171_11disease resistance protein rpm1 homolog
Ta_S58859323	14.8	0.041305	Not found
Ta_S22373503	14.5	0.035288	Hsf-type dna-binding domain containing expressed
Ta_S58884158	14.0	5.29E-09	Cytochrome p450 liketbp
contig_4027	13.8	0.012235	Hsf-type dna-binding domain containing expressed
Ta_S58887920	12.5	0.018728	Pentatricopeptide repeat-containing protein mitochondrial-like
Ta_S37815739	12.4	5.25E-05	Caffeic acid 3-o-
Ta_S58844263	12.1	0.015089	Not found
Ta_S17982440	12.1	3.85E-11	Serine acetyltransferase
Ta_S58846190	11.9	0.006763	La domain family
Ta_S37845209	10.5	0.01791	Not found
Ta_S32681735	10.3	0.000194	Not found
Ta_S32654208	9.9	0.005766	Not found
contig_565	9.7	0.013784	Not found
Ta_S58857445	9.4	0.009264	Serine threonine-protein kinase
Ta_S58895571	8.9	0.002269	Not found
Ta_S16248940	8.5	0.017956	Not found
contig_312	8.5	1.58E-05	Not found
Ta_S58889424	8.0	0.001986	Not found
Ta_S58891564	7.9	6.03E-05	Cytochrome p450 liketbp
Ta_S58856591	7.6	0.033515	Not found
Ta_S59490000	6.9	0.046498	Myb transcription factor
Ta_S13230809	6.6	0.015398	Not found
Ta_S32507710	6.2	4.26E-06	Heat shock cognate 70 kda expressed
Ta_S58842184	5.7	0.028196	Serine threonine-protein kinase rio1-like
Ta_S12902351	5.7	0.040947	Endo-beta- -glucanase
contig_4604	5.6	0.044513	Aberrant pollen transmission
Ta_S58859081	5.5	0.002069	Protein
Ta_S13177111	5.4	0.023428	Not found
Ta_S58839733	5.3	0.009311	Rna pseudourine synthase 7
Ta_S13203445	5.3	0.007348	Not found
Ta_S13130963	5.3	0.003439	Not found

Ta_S13128615	5.0	0.005439	Not found
contig_5105	5.0	0.002405	Calcium-transporting atpase plasma membrane-type-like
contig_5384	4.9	6.71E-05	Not found
contig_3896	4.9	0.023133	Atp synthase f0 subunit 6
contig_2359	4.8	0.0422	Not found
Ta_S58906848	4.8	0.035364	Not found
contig_3292	4.7	0.001588	Ribonucleotide reductase
Ta_S58850217	4.7	0.035126	Pentatricopeptide repeat-containing protein
Ta_S58851704	4.6	0.019086	Dna helicase
Ta_S52543987	4.5	0.021261	F-box protein at5g07610-like
contig_2239	4.5	0.000884	Abc transporter b family member 20-like
Ta_S12999712	4.5	0.004115	Retrotransposon unclassified
Ta_S16246863	4.4	0.048563	F-box protein at5g52880-like
Ta_S50388141	4.4	0.000164	Not found
contig_5480	4.3	0.001514	Dna repair protein rad50
Ta_S13165985	4.3	0.027464	Auxin-induced protein 15a
Ta_S58862793	4.3	0.049535	Not found
contig_3750	4.3	0.006817	Transformation transcription domain-associated protein
Ta_S13177057	4.2	0.009059	Protein (low-affinity nitrate transporter)
contig_2223	4.2	0.012425	Not found
contig_4749	4.2	0.012448	Not found
Ta_S13243932	4.2	0.010607	Protein
Ta_S13167109	4.2	0.016915	Not found
Ta_S13227407	4.1	0.0135	Not found
Ta_S58857760	4.1	0.015583	Not found
Ta_S22371585	4.1	0.020454	Not found
Ta_S58842370	4.0	0.015452	Gdp-mannose transporter
contig_6225	3.9	0.001586	Terminal uridylyltransferase 7-like
contig_4425	3.9	0.003578	Not found
Ta_S37759224	3.9	0.006323	Bowman-birk trypsin inhibitor-like protein
Ta_S19223311	3.8	0.000329	Snf2 super family
contig_4264	3.8	0.000279	Not found
Ta_S58862912	3.8	0.023386	Not found

Ta_S16192587	3.8	0.01741	Not found
contig_4719	3.7	0.028446	Hypothetical protein RAZWK3B_00585 [Roseobacter sp. Azwk-3b]
contig_2770	3.7	0.0001	Beta-amyrin synthase
Ta_S13166085	3.7	0.022504	Not found
Ta_S17888799	3.7	0.004178	PREDICTED: uncharacterized protein LOC100839923 [Brachypodium distachyon]
contig_3741	3.7	0.00033	Beta-expansin 2 precursor
Ta_S58853302	3.7	0.017175	Not found
contig_1173	3.7	0.008315	Not found
Ta_S58853221	3.7	0.019972	PREDICTED: uncharacterized protein LOC100835027 [Brachypodium distachyon]
Ta_S12888461	3.7	0.043034	Not found
Ta_S21611732	3.6	0.038114	Not found
contig_6844	3.6	0.047101	Not found
contig_5883	3.6	0.007309	Amino acid permease family expressed
contig_5199	3.6	0.002266	Disease resistance protein rpm1-like
Ta_S58868812	3.6	0.034298	Epstein-barr virus ebna-1-like protein
Ta_S13144009	3.6	0.045792	60s ribosomal protein l34
Ta_S32530535	3.5	0.002146	Not found
contig_896	3.5	0.000232	Not found
Ta_S32510701	3.5	0.023986	Mitogen-activated protein kinase kinase 6-like (MAPK)
contig_1850	3.5	0.002	Casein kinase i isoform delta-like
Ta_S58861829	3.4	0.011656	Beta-glucosidase 5-like isoform 2
Ta_S22370985	3.4	0.022688	Predicted protein [Hordeum vulgare subsp. Vulgare]
contig_4443	3.4	0.045306	Not found
contig_6114	3.4	0.031475	Mac perforin domain containing protein
contig_2182	3.4	0.007848	Protein
Ta_S32501583	3.4	0.016771	Protein
Ta_S58848661	3.3	0.025071	Not found
Ta_S58846759	3.3	0.031009	Not found
contig_3927	3.3	0.042921	Not found
contig_1285	3.3	0.048853	Microtubule organization protein
Ta_S13146836	3.3	0.015823	60s ribosomal protein l7-1

Ta_S58884395	3.2	9.90E-05	Pg1 protein
Ta_S17981880	3.2	0.006262	Not found
contig_3422	3.2	0.043532	Hth dna-binding expressed
Ta_S58846451	3.2	0.022381	Not found
Ta_S58894107	3.2	0.024519	Amino acid permease
Ta_S13172731	3.2	0.046943	Cytochrome c oxidase polypeptide vib
Ta_S32667864	3.2	0.016896	Protein
contig_6563	3.2	0.035511	Cytochrome p450 71e1
Ta_S22381097	3.2	0.005502	Otu-like cysteine protease family protein
Ta_S58899343	3.1	0.003699	Metal transporter nramp6-like
contig_6095	3.1	0.00717	Not found
contig_1160	3.1	0.013055	Brefeldin a-inhibited guanine nucleotide-exchange protein 1-like
contig_2296	3.1	0.006482	Hypothetical protein osi_11910 [Oryza sativa Indica Group]
Ta_S17976596	3.1	0.008875	Sulphate transporter
Ta_S58857404	3.1	0.036759	PREDICTED: uncharacterized protein LOC100842944 [Brachypodium distachyon]
Ta_S58841493	3.1	0.022736	Retrotransposon unclassified
Ta_S17890893	3.1	0.039251	Glutamine synthetase
contig_4775	3.1	0.000578	2-aminoethanethiol dioxygenase-like
contig_3720	3.0	0.014501	Not found
Ta_S58851753	3.0	0.026695	Tesmin tso1-like cxc domain containing expressed
Ta_S13048497	3.0	0.039859	Reticulon
contig_4741	3.0	0.007305	Embryogenesis-associated protein emb8-like
contig_1001	3.0	0.00441	Not found
contig_2667	3.0	0.045329	Not found
Ta_S17897607	2.9	0.018235	Major facilitator protein
Ta_S43485972	2.9	0.010123	Protein phosphatase 2c
contig_2113	2.9	0.013425	Speckle-type poz protein
contig_6873	2.9	0.018253	Predicted protein [Hordeum vulgare subsp. Vulgare]
contig_4732	2.9	0.024764	Mrna-capping enzyme
contig_5266	2.9	0.012367	Erect panicle 2 partial
contig_3994	2.9	0.026436	Nodulin-like protein
Ta_S37772528	2.9	0.049742	Minor histocompatibility antigen h13

Ta_S58851000	2.9	0.017112	Probable lrr receptor-like serine threonine-protein kinase at3g47570-like
Ta_S14911635	2.9	0.047019	Not found
Ta_S13138899	2.9	0.002761	Not found
contig_237	2.9	0.008549	32 kda protein
Ta_S13165765	2.9	0.009439	Not found
contig_4540	2.9	0.028159	Os02g0159200 [Oryza sativa Japonica Group]
contig_4292	2.9	0.006344	Nb-arc domain containing expressed
contig_3393	2.9	0.034167	Not found
Ta_S58860687	2.9	0.006996	Ulp1 protease
contig_3183	2.9	0.04096	Phosphatidylinositol -kinase-like
contig_2394	2.9	0.024308	Nbs-lrr partial
contig_3573	2.9	0.0236	Transposon expressed
Ta_S16199319	2.9	0.023912	Not found
contig_1438	2.9	0.037843	Not found
contig_1600	2.9	0.04453	Rna recognition motif family expressed
Ta_S32526552	2.8	0.04364	Not found
Ta_S32659341	2.8	0.030422	Ribosomal protein s12
contig_614	2.8	0.010706	Not found
contig_3699	2.8	0.02532	Histidine kinase- dna gyrase b- and hsp90-like atpase family protein
Ta_S26021394	2.8	0.040444	Not found
contig_4303	2.8	0.029942	Not found
contig_814	2.8	0.009313	Not found
Ta_S58840365	2.7	0.036137	Not found
Ta_S13046163	2.7	0.018747	Pppde peptidase domain-containing protein 2-like
Ta_S58894413	2.7	0.035843	6-phosphofructokinase 4
contig_3852	2.7	0.020831	Calpain-like protein
contig_2943	2.7	0.017313	Nadh-ubiquinone oxidoreductase subunit
Ta_S58851084	2.7	0.026077	Not found
contig_2700	2.7	0.015973	Kinase-interacting protein 1
Ta_S58864379	2.7	0.02852	Poly polymerase-like
Ta_S26022473	2.7	0.017016	Zinc-finger protein

Ta_S13140457	2.7	0.026444	Not found
Ta_S13124877	2.7	0.001877	Initiation factor
contig_3450	2.7	0.004636	Predicted protein [Hordeum vulgare subsp. Vulgare]
contig_6410	2.7	0.006995	Aldose reductase
contig_31	2.6	0.036198	Vacuolar protein sorting 13c
Ta_S58885340	2.6	0.006231	Dynamin-related protein 1e
Ta_S58858194	2.6	0.028588	Leukocyte receptor cluster
contig_1142	2.6	0.04773	Blight resistance protein
Ta_S32626973	2.6	0.029205	Peptide transporter ptr2-like
contig_3362	2.6	0.002923	Predicted protein [Hordeum vulgare subsp. Vulgare]
Ta_S58890953	2.6	0.04389	Not found
Ta_S58847478	2.6	0.028481	Nuclear factor related to kappa-b-binding protein
contig_708	2.6	0.041728	Secondary cell wall-related glycosyltransferase family 47
contig_6208	2.6	0.034429	Not found
Ta_S58839330	2.6	0.023975	Hypothetical protein osi_07633 [Oryza sativa Indica Group]
contig_5714	2.6	0.039284	Actin depolymerizing factor 4
contig_1361	2.6	0.040104	Not found
contig_7159	2.6	0.023099	Serine carboxypeptidase
Ta_S32526666	2.6	0.017006	Dehydration stress-induced protein
Ta_S17979451	2.6	0.023471	Magnesium transporter nipa2
Ta_S58861380	2.5	0.045323	G-type lectin s-receptor-like serine threonine-protein kinase rks1-like
Ta_S16212763	2.5	0.019474	Galactose-1-phosphate uridylyltransferase
Ta_S32667278	2.5	0.019801	Not found
Ta_S17889461	2.5	0.027488	Sphingoid long-chain bases kinase 1
contig_2612	2.5	0.031927	Cytochrome c oxidase assembly protein
contig_3696	2.5	0.005704	U5 small nuclear ribonucleoprotein 200 kda helicase
Ta_S13141197	2.5	0.035223	O-methyltransferase zrp4
Ta_S12998046	2.5	0.023305	Nadh-plastoquinone oxidoreductase subunit k
Ta_S13146753	2.5	0.025228	60s ribosomal protein l34
Ta_S22378208	2.5	0.031434	Beta-amyrin synthase
Ta_S58850705	2.5	0.041082	Dna-directed rna polymerase e subunit 1-like
Ta_S58882670	2.5	0.039607	Serine protease-like protein

Ta_S12906375	2.5	0.034624	Mitochondrial substrate carrier family protein
contig_2456	2.5	0.021233	Leaf senescence related
Ta_S12977114	2.4	0.000897	Rrna intron-encoded homing endonuclease
Ta_S17893420	2.4	0.008429	Beta-expansin 1a expressed
Ta_S26028788	2.4	0.03883	Somatic embryogenesis receptor kinase
Ta_S16267847	2.4	0.040243	Hsf-type dna-binding domain containing expressed
contig_2743	2.4	0.037249	Tbc domain containing protein
contig_2382	2.4	0.045883	Transcription factor bhlh128
Ta_S24512908	2.4	0.039708	Not found
contig_2923	2.4	0.032901	Callose synthase
contig_2142	2.4	0.037606	Hypothetical protein osi_15330 [Oryza sativa Indica Group]
Ta_S58895262	2.4	0.041213	Protein
contig_3502	2.4	0.030821	Vacuolar protein sorting 13c
Ta_S58846204	2.4	0.047479	Not found
Ta_S37811862	2.4	0.021538	Dna binding protein
Ta_S58853267	2.4	0.012625	Lipid-binding serum glyco protein
contig_2635	2.4	0.020216	Abc transporter c family member 9-like
Ta_S50381169	2.4	0.049165	Btb poz domain-containing protein at1g04390-like
Ta_S17977835	2.4	0.03057	Cytochrome b561
contig_3132	2.4	0.027554	Sister chromatid cohesion protein pds5
contig_56	2.4	0.014155	Atp-dependent clp protease atp-binding subunit
Ta_S20999571	2.3	0.030328	Probable xyloglucan endotransglucosylase hydrolase protein 8-like
Ta_S32516159	2.3	0.032981	Not found
contig_7109	2.3	0.008957	Glutamine expressed
contig_2242	2.3	0.035709	Premnaspirodiene oxygenase-like
Ta_S17880905	2.3	0.048284	50s ribosomal protein l33
Ta_S13148233	2.3	0.015525	60s ribosomal protein l34
Ta_S26028472	2.3	0.043489	Nbs-lrr disease resistance protein homologue
contig_2730	2.3	0.026903	Chd3-type chromatin-remodeling factor pickle-like
contig_6511	2.3	0.049041	Wd repeat-containing protein
Ta_S12983024	2.3	0.04741	Golgin candidate 5-like
contig_619	2.3	0.032178	Protein
contig_5981	2.3	0.019981	Serine threonine-protein kinase sapk3-like

Ta_S32590426	2.3	0.003332	Nadh-ubiquinone oxidoreductase chain
Ta_S37852405	2.3	0.00411	Protein kinase xa21
Ta_S32655890	2.3	0.02744	Protein
Ta_S32683311	2.2	0.031013	Ternary complex factor mip1-like
Ta_S58886404	2.2	0.035635	Tbc domain containing protein
contig_5002	2.2	0.013931	Not found
Ta_S17512385	2.2	0.047833	Not found
Ta_S37937220	2.2	0.027311	Hypothetical protein SORBIDRAFT_04g028380 [Sorghum bicolor]
Ta_S58860293	2.2	0.03301	Ubx domain-containing protein
contig_791	2.2	0.00475	Regulator of nonsense transcripts 1 homolog
contig_6109	2.2	0.039413	Probable importin subunit beta-4-like
contig_6922	2.2	0.030893	Poly polymerase catalytic domain containing expressed
Ta_S58895865	2.2	0.04609	Not found
Ta_S52542495	2.2	0.023677	Cs 5
contig_2968	2.2	0.036214	Eukaryotic translation initiation factor 3 subunit c-like
Ta_S17890867	2.2	0.015013	Os08g0110800 [Oryza sativa Japonica Group]
contig_4114	2.2	0.048739	PREDICTED: uncharacterized protein LOC100827929 [Brachypodium distachyon]
Ta_S13012043	2.2	0.030101	Diphosphonucleotide phosphatase expressed
contig_2240	2.2	0.048342	Fkbp12-rapamycin complex-associated protein
Ta_S58855127	2.2	0.023961	Protein far1-related sequence 12-like
Ta_S47005448	2.2	0.004909	Sterol c-14 reductase
contig_2914	2.2	0.035397	Protease do-like 7-like
contig_7023	2.2	0.034854	Acc oxidase
contig_5777	2.2	0.034466	Beach domain-containing protein lvsa-like
contig_2895	2.2	0.027153	Serine threonine-protein kinase sapk2
Ta_S13043514	2.1	0.024076	PREDICTED: uncharacterized protein LOC100841045 [Brachypodium distachyon]
contig_2509	2.1	0.024492	Not found
contig_6340	2.1	0.016588	Protein
Ta_S22387577	2.1	0.010013	Thioredoxin-like 4a
contig_4225	2.1	0.035959	Calcium-transporting atpase endoplasmic reticulum-type-like
contig_4592	2.1	0.029819	Not found

contig_354	2.1	0.040756	Cytochrome b5 reductase 4-like
contig_691	2.1	0.027289	U3 small nucleolar rna-associated protein 10 and nuc211 domain-containing protein
Ta_S32635933	2.1	0.039078	Nodulin-related partial
contig_4052	2.1	0.045423	Arp2 3 complex 34 kda subunit
Ta_S22378449	2.1	0.018241	Protein
Ta_S32565776	2.1	0.010922	Duf246 domain-containing protein at1g04910-like
contig_3678	2.1	0.03295	Not found
Ta_S58881778	2.1	0.045461	Not found
Ta_S58900180	2.1	0.042857	Solute carrier family 35 member f1-like
Ta_S58887074	2.1	0.027853	Protein
Ta_S12965080	2.0	0.031017	Atp synthase subunit beta
Ta_S58865342	2.0	0.046041	Protein far1-related sequence 5-like
contig_1241	2.0	0.041959	Predicted protein [Hordeum vulgare subsp. Vulgare]
Ta_S58902295	2.0	0.03139	Seven transmembrane domain protein
contig_454	2.0	0.034425	Cop1-interacting protein
Ta_S13135140	2.0	0.044097	Not found
contig_2564	2.0	0.009796	Not found
contig_1395	2.0	0.033116	Kinesin heavy chain
Ta_S12971663	2.0	0.036675	Carrier protein
contig_857	2.0	0.035726	Gdp-mannose pyrophosphorylase
contig_395	2.0	0.043037	Disease resistance protein rga4-like
contig_2601	2.0	0.021933	Protein kinase
contig_2033	2.0	0.039417	Not found
contig_3412	2.0	0.014033	Not found
Ta_S37823504	2.0	0.040924	Not found
Ta_S12924295	2.0	0.043982	Formiminotransferase- expressed
contig_1621	2.0	0.034748	Nadh-ubiquinone oxidoreductase
Ta_S58864643	(-)ND	0.002036	Retrotransposon unclassified
Ta_S58849115	(-)ND	0.017092	Retrotransposon unclassified
Ta_S13223809	(-)ND	0.046522	Ring-box protein
Ta_S13149798	(-)ND	0.014972	Transmembrane bax inhibitor motif-containing protein 4
Ta_S58912535	(-)ND	0.033338	Wak1 - receptor-like cytoplasmic kinase (-rlck) precursor defense

			response to fungus
Ta_S32656390	(-)ND	0.00326	Zinc finger protein id1
contig_6889	(-)ND	0.005749	Not found
contig_6774	(-)ND	0.044708	Not found
Ta_S32605842	(-)ND	0.001484	Not found
Ta_S58841817	(-)ND	0.001981	Not found
Ta_S13128736	(-)ND	0.00209	Not found
Ta_S37864864	(-)ND	0.0035	Not found
Ta_S58845207	(-)ND	0.003902	Not found
Ta_S58854505	(-)ND	0.006751	Not found
Ta_S13181029	(-)ND	0.009241	Not found
Ta_S58845845	(-)ND	0.009801	Not found
Ta_S58898595	(-)ND	0.011382	Not found
Ta_S58852808	(-)ND	0.011659	Not found
Ta_S13143288	(-)ND	0.014402	Not found
Ta_S32611510	(-)ND	0.014905	Not found
Ta_S32629074	(-)ND	0.016429	Not found
Ta_S13031432	(-)ND	0.018661	Not found
Ta_S32519859	(-)ND	0.018718	Not found
Ta_S37936079	(-)ND	0.0191	Not found
Ta_S32515094	(-)ND	0.028336	Not found
Ta_S58848140	(-)ND	0.031894	Not found
Ta_S58912831	(-)ND	0.033668	Not found
Ta_S13173968	(-)ND	0.033907	Not found
Ta_S13129446	(-)ND	0.039654	Not found
contig_1563	296.8	0.005116	Hypothetical protein EB105725_31_00165 [Escherichia blattae NBRC 105725]
Ta_S17989215	224.8	0.012153	Not found
Ta_S18007616	86.1	0.035934	F-box protein interaction domain containing protein
contig_122	74.4	0.020042	Kgg repeat-containing stress-induced protein
contig_7138	47.7	0.000812	PREDICTED: uncharacterized protein LOC100828580 [Brachypodium distachyon]
Ta_S58889718	34.9	0.000155	Not found

Ta_S22379260	33.3	2.83E-05	Predicted protein [Hordeum vulgare subsp. Vulgare]
Ta_S58851470	29.3	0.008616	Not found
Ta_S37803822	28.8	0.049691	Upf0481 protein at3g47200-like
Ta_S58855143	23.4	0.005618	Pentatricopeptide repeat-containing protein
Ta_S32562131	22.0	0.006854	Not found
Ta_S13041446	20.0	0.036236	Not found
Ta_S58888744	19.4	0.011021	Not found
Ta_S13119248	18.9	0.03781	Not found
Ta_S58893909	18.8	0.01346	Not found
Ta_S58842447	17.9	0.016019	PREDICTED: uncharacterized protein LOC100837593 [Brachypodium distachyon]
Ta_S13132385	17.4	0.005321	Like protein
Ta_S58862139	16.2	0.017777	Not found
Ta_S58869437	15.7	0.001733	Not found
Ta_S58865123	15.2	0.002555	Not found
Ta_S58894064	15.1	0.022629	Tvp38 tmem64 family membrane protein slr0305-like
Ta_S58863212	14.9	0.042984	Not found
Ta_S50387228	14.3	0.020233	Not found
Ta_S58846533	14.3	0.041675	Nodulin-like protein
Ta_S13180058	13.8	0.038596	Not found
Ta_S58854706	13.2	0.007929	Bwf1-like protein
contig_2145	12.9	0.000596	Elongation factor ts
Ta_S58866498	11.9	0.011321	Not found
contig_6805	11.4	0.00084	Not found
Ta_S37854435	11.4	0.000559	Anthocyanidin -o-glucosyltransferase-like
contig_6985	10.1	0.000141	Hypothetical protein SORBIDRAFT_03g008680 [Sorghum bicolor]
Ta_S58845691	9.9	0.033479	Mutant low phytic acid protein 1
Ta_S58849116	9.8	0.008202	Not found
Ta_S16231538	9.5	0.000544	PREDICTED: uncharacterized protein LOC100841809 [Brachypodium distachyon]
Ta_S58865733	9.1	0.010126	Hypothetical protein osj_31200 [Oryza sativa Japonica Group]
Ta_S58896701	9.0	0.007022	Not found
contig_6324	8.9	0.032258	Protein yeez

Ta_S12913717	8.8	0.00634	Dna-directed rna polymerases i and iii subunit rpac1-like
Ta_S12882577	8.6	0.009406	Ring-h2 finger protein atl1n precursor
contig_1201	8.5	0.01555	Not found
Ta_S58848010	8.1	0.02126	Not found
contig_1642	8.0	0.041335	PREDICTED: uncharacterized protein LOC100826907 [Brachypodium distachyon]
Ta_S32622948	7.9	0.000312	Late embryogenesis abundant protein
Ta_S13014352	7.8	0.017039	Protein suppressor of gene silencing-like protein
contig_6887	7.7	0.000537	Not found
Ta_S58858052	7.7	0.019978	PREDICTED: uncharacterized protein LOC100829225 [Brachypodium distachyon]
Ta_S13119076	7.7	0.027102	Predicted protein [Hordeum vulgare subsp. Vulgare]
Ta_S58909995	7.7	0.002892	Bzip transcription factor family expressed
contig_2040	7.7	0.002061	Transferring glycosyl
Ta_S17979419	7.5	0.019694	Dna ligase-like
Ta_S16246857	7.3	0.006151	Disease resistance protein rpm1-like
Ta_S13183409	7.2	0.00982	Not found
Ta_S32568379	7.2	0.002987	Not found
Ta_S58847149	7.0	0.009853	Not found
Ta_S13012191	6.9	0.02941	Predicted protein [Hordeum vulgare subsp. Vulgare]
Ta_S13262842	6.9	0.014523	PREDICTED: uncharacterized protein LOC100827899 [Brachypodium distachyon]
contig_5628	6.8	0.023393	Cytochrome c heme attachment protein
Ta_S58839265	6.7	0.003925	Not found
contig_2680	6.7	0.015497	PREDICTED: uncharacterized protein LOC100827734 [Brachypodium distachyon]
Ta_S58882413	6.6	0.000605	Protein
Ta_S12957883	6.5	0.025181	Not found
contig_4166	6.4	0.009352	Not found
Ta_S58888286	6.4	0.002662	Protein chloroplast import apparatus 2-like
contig_1287	6.4	0.036626	Protein ddb_g0268328-like
Ta_S58852095	6.4	0.012195	Not found
contig_3460	6.3	0.04975	Not found

Ta_S58909884	6.3	0.007486	PREDICTED: uncharacterized protein LOC100826789 isoform 2 [Brachypodium distachyon]
Ta_S58889807	6.2	0.039403	Global transcription factor group
contig_2233	6.2	7.39E-05	Not found
Ta_S58847793	6.2	0.008982	Not found
Ta_S13179757	6.1	0.002234	Not found
contig_6194	6.0	0.000574	Protein furry homolog-like
Ta_S12894932	5.9	0.003389	Vip3 protein
Ta_S58894572	5.9	0.028102	Not found
contig_506	5.8	4.72E-05	Rna polymerase beta subunit
Ta_S58841053	5.8	0.039817	Not found
Ta_S58890145	5.8	0.010066	L-asparaginase
contig_6486	5.8	0.011558	Not found
Ta_S58855771	5.7	0.037754	Not found
Ta_S13111509	5.7	0.024983	Calmodulin
Ta_S16248415	5.7	0.010506	Not found
Ta_S52543421	5.6	0.002692	Protein
contig_6115	5.5	0.011847	Cytochrome c biogenesis protein precursor
contig_1932	5.5	0.032824	Zinc finger-like
Ta_S32564414	5.5	0.016276	Predicted protein [Hordeum vulgare subsp. Vulgare]
Ta_S19116911	5.5	0.017142	Fca protein
Ta_S26025255	5.4	0.012003	Photosystem ii reaction center w protein
Ta_S13179255	5.3	0.017007	Not found
Ta_S32565615	5.2	0.00308	Not found
contig_6267	5.1	8.63E-05	Not found
contig_1068	5.1	0.00032	Not found
Ta_S58884786	5.0	0.034851	Flavonol 3-sulfotransferase
Ta_S13043420	5.0	0.01845	Protein
Ta_S52545275	4.9	0.008435	Ulp1+protease+family [Oryza glaberrima]
Ta_S13137206	4.9	0.021794	Not found
Ta_S59490003	4.9	0.040244	Myb transcription factor
Ta_S58907457	4.9	0.000209	Predicted protein [Hordeum vulgare subsp. Vulgare]
Ta_S26023560	4.8	0.014513	Not found

Ta_S16213897	4.8	0.014501	Insulin-degrading enzyme-like
Ta_S17978397	4.7	0.049031	Casp-like protein stg-like
Ta_S58890169	4.7	0.017786	Fk506 binding protein
contig_3086	4.7	9.64E-05	Coiled-coil domain-containing protein 132-like
Ta_S58886646	4.7	0.016325	Phytosulfokines 2-like
Ta_S53281796	4.6	0.004466	Partial
Ta_S13194862	4.6	0.034102	Not found
Ta_S32671427	4.6	0.00369	Not found
Ta_S37850818	4.6	0.047891	Blue copper binding protein
contig_387	4.5	0.015073	Peroxidase 1 precursor
contig_1359	4.5	0.007121	Not found
Ta_S58869803	4.5	0.022403	Something about silencing protein
Ta_S58885579	4.5	0.013609	Retrotransposon ty3-gypsy expressed
Ta_S58886383	4.5	0.038333	Not found
Ta_S58895529	4.4	0.013951	Probable fructokinase-1-like
Ta_S58853931	4.4	0.004754	Not found
contig_3430	4.4	0.035664	PREDICTED: uncharacterized protein LOC100835632 [Brachypodium distachyon]
Ta_S58854350	4.4	0.003766	Not found
Ta_S16199865	4.4	0.005065	Predicted protein [Hordeum vulgare subsp. Vulgare]
Ta_S58839592	4.4	0.022398	Not found
Ta_S52543823	4.4	0.014655	Glycolate oxidase
Ta_S37845220	4.3	0.027409	Predicted protein [Hordeum vulgare subsp. Vulgare]
Ta_S16203218	4.3	0.031188	G-patch domain containing protein
Ta_S58845015	4.3	0.035904	Not found
Ta_S13194190	4.3	0.008729	Not found
Ta_S32602541	4.3	0.035606	Wrky45-like transcription factor
Ta_S52541979	4.3	0.005915	Mazg nucleotide pyrophosphohydrolase domain protein
contig_6543	4.2	0.024677	Abc transporter g family member 16-like
contig_4210	4.2	0.003053	Not found
contig_2469	4.1	0.0154	Not found
contig_6647	4.1	0.022231	Not found
Ta_S58856084	4.0	0.006992	Protein raspberry 3

contig_2463	4.0	0.020513	Nac domain-containing protein 21 22-like
Ta_S52546230	4.0	0.02679	Predicted protein [Hordeum vulgare subsp. Vulgare]
Ta_S55352938	4.0	0.042183	Not found
Ta_S58894026	4.0	0.006073	Protein mei2-like 2-like
Ta_S32564964	4.0	0.015267	Lob domain-containing protein 12-like
Ta_S18008276	4.0	0.02317	Metallothionein
contig_4435	3.9	0.001184	Not found
Ta_S52542038	3.9	0.011996	Not found
contig_3797	3.9	0.047636	Not found
Ta_S58857043	3.9	0.046382	Not found
Ta_S13011770	3.9	0.016041	Response regulator receiver domain containing expressed
Ta_S32529059	3.9	0.044464	P-glycoprotein 1
Ta_S58862129	3.9	0.015591	Ubiquitin carboxyl-terminal hydrolase 5-like
contig_6139	3.9	0.017725	PREDICTED: uncharacterized protein LOC100830293 [Brachypodium distachyon]
Ta_S37773901	3.9	0.020961	Cytochrome p450
contig_4099	3.9	0.011792	Not found
Ta_S58884172	3.8	0.019041	Sugar transporter family expressed
Ta_S26022483	3.8	0.004652	Mrna turnover protein 4 homolog
contig_2117	3.8	0.036091	Protein far1-related sequence 6-like
Ta_S58900106	3.8	0.036143	Light-harvesting complex i chlorophyll a b binding protein 3
contig_177	3.8	0.021501	Not found
Ta_S22370925	3.7	0.028464	Cyclin-dependent protein
contig_4121	3.7	0.003503	Nadh-plastoquinone oxidoreductase subunit 4
contig_189	3.6	0.002803	Hydroxypyruvate reductase
contig_3206	3.6	0.01727	Not found
contig_1921	3.6	0.031429	Not found
Ta_S17057489	3.6	0.012101	Auxin response factor 3-like
Ta_S37847348	3.6	0.046873	Not found
contig_6073	3.6	0.000429	Not found
Ta_S17984780	3.6	0.038704	Rna recognition motif-containing protein
Ta_S58847292	3.6	0.044602	Not found
Ta_S17990036	3.5	0.012441	Os01g67210-like protein

contig_4485	3.5	0.011469	Protein
contig_34	3.5	0.007414	Ac079179_7 dna2-nam7 helicase family protein
Ta_S13192404	3.5	0.002451	Not found
contig_5565	3.5	0.013811	Not found
Ta_S16265918	3.5	0.034256	Not found
contig_6371	3.5	0.003753	Cleavage and polyadenylation specificity factor subunit 1-like
contig_5623	3.5	0.015428	Not found
Ta_S32623645	3.5	0.005422	Not found
contig_4508	3.5	0.026426	Not found
contig_3771	3.5	0.045802	Not found
Ta_S13051479	3.4	0.02613	Serine threonine-protein kinase afc1
contig_667	3.4	0.007296	Glucan endo- -beta-glucosidase 3 precursor
Ta_S13158111	3.4	0.038946	3 -n-debenzoyl-2 -deoxytaxol n-benzoyltransferase-like
contig_6266	3.4	0.026402	PREDICTED: uncharacterized protein LOC100846531 [Brachypodium distachyon]
Ta_S22368361	3.4	0.030739	Not found
Ta_S17985298	3.4	0.030577	Os03g0806600 [Oryza sativa Japonica Group]
contig_6429	3.4	0.001115	Atp-dependent clp protease atp-binding subunit -like
contig_5972	3.4	0.036929	Kinesin-like protein
contig_6711	3.4	0.001579	Ring finger protein 10-like
contig_1657	3.4	0.001289	PREDICTED: uncharacterized protein LOC100833724 [Brachypodium distachyon]
contig_4494	3.4	0.004922	Adomet-dependent rrna methyltransferase spb1-like
Ta_S35718003	3.3	0.034433	Cbf protein 4
contig_2829	3.3	0.016477	Disease resistance protein rpp13-like
Ta_S52544017	3.3	0.000181	Os11g0205500 [Oryza sativa Japonica Group]
Ta_S52541619	3.3	0.003758	Predicted protein [Hordeum vulgare subsp. Vulgare]
contig_976	3.3	0.017469	Not found
Ta_S32680735	3.3	0.046318	Atp-dependent helicase
contig_893	3.3	0.026324	Hypothetical protein SORBIDRAFT_02g042020 [Sorghum bicolor]
contig_7150	3.3	0.043671	Pectinacetylesterase precursor
contig_5790	3.3	0.00218	Atp gtp binding protein
Ta_S12937455	3.3	0.02511	Caffeic acid 3-o-methyltransferase

contig_6028	3.3	0.047501	Zinc finger-like
contig_6790	3.2	0.002154	Not found
Ta_S58885047	3.2	0.043247	Required to maintain repression 1
contig_1204	3.2	0.020006	Not found
Ta_S58890225	3.2	0.032208	Pinus taeda anonymous locus 0_13978_01 genomic sequence
Ta_S58892307	3.2	0.036361	Protein
Ta_S12978479	3.2	0.025629	Upf0503 protein chloroplastic-like
Ta_S52544593	3.2	0.008103	Iaa amidohydrolase
Ta_S58891200	3.2	0.049555	Probable calcium-binding protein cml45-like
Ta_S58843579	3.2	0.012423	Zinc finger c-x8-c-x5-c-x3-h type family protein
contig_5459	3.2	0.0118	Not found
contig_6622	3.2	0.033575	Predicted protein [Hordeum vulgare subsp. Vulgare]
contig_1887	3.2	0.029589	Nadh-plastoquinone oxidoreductase subunit i
contig_1450	3.1	0.024256	Not found
contig_6247	3.1	0.031666	Ubiquitin-protein pub49
contig_5892	3.1	0.03285	Tgf-beta type i ii extracellular region
Ta_S32611416	3.1	0.002956	Not found
contig_1268	3.1	0.004966	Pre-mrna-processing factor 6-like
Ta_S58848668	3.1	0.022083	Protein
Ta_S58839681	3.1	0.005448	Molybdopterin cofactor
Ta_S52543429	3.1	0.048621	Far-red impaired response
contig_510	3.1	0.016319	Not found
contig_3567	3.1	0.046643	Histidine kinase 1
Ta_S12898462	3.1	0.048414	Not found
contig_595	3.1	0.016907	Not found
Ta_S16213725	3.1	0.024768	Not found
contig_206	3.1	0.040605	Wall-associated receptor kinase 3-like
Ta_S52541449	3.1	0.015366	Acc oxidase
Ta_S58855004	3.0	0.022203	Retrotransposon unclassified
Ta_S52541238	3.0	0.036684	Protein
Ta_S58908965	3.0	0.04018	Not found
contig_4372	3.0	0.010219	PREDICTED: uncharacterized protein LOC100830560 [Brachypodium distachyon]

contig_6211	3.0	0.023654	Not found
contig_1319	3.0	0.001602	Protein
Ta_S58849836	3.0	0.019897	Transposon en spm sub-class
Ta_S12971695	3.0	0.038384	Not found
Ta_S52545353	3.0	0.026062	Gene product
contig_2007	3.0	0.002687	S-locus protein 4
contig_4736	3.0	0.04582	Transparent testa 12 protein
contig_4368	3.0	0.025215	Pleckstrin homology domain-containing protein
Ta_S58863463	3.0	0.044705	Not found
contig_1928	3.0	0.003062	Protein kinase
Ta_S58850183	3.0	0.043876	Protein rrp5 homolog
Ta_S32571600	3.0	0.019429	Aaa atpase containing von willebrand factor type a precursor
Ta_S26018764	3.0	0.028686	Not found
Ta_S16207731	2.9	0.024887	Aspartic proteinase nepenthesin-1-like
Ta_S37827906	2.9	0.039072	Not found
contig_6944	2.9	0.011773	Not found
contig_5588	2.9	0.040374	Auxilin-related protein 2-like
contig_1953	2.9	0.006278	Not found
contig_3846	2.9	0.002502	Not found
Ta_S22381029	2.9	0.03341	Cytochrome p450
Ta_S16262691	2.9	0.024578	Predicted protein [Hordeum vulgare subsp. Vulgare]
contig_4488	2.8	0.042037	Not found
Ta_S17897290	2.8	0.003737	Nascent polypeptide associated complex alpha chain
Ta_S26023404	2.8	0.041541	Plastid protein
contig_1298	2.8	0.035525	Low quality protein: 1-phosphatidylinositol-3-phosphate 5-kinase-like
Ta_S26026252	2.8	0.027495	50s ribosomal protein l9
Ta_S17987500	2.8	0.043801	Cytochrome p450 probable 6-deoxoteasterone to 3-dehydro 6-deoxoteasterone or teasterone to 3-dehydro teasterone
contig_85	2.8	0.018126	Not found
Ta_S26022199	2.8	0.038098	Not found
contig_6724	2.8	0.03668	Os05g0207900 [Oryza sativa Japonica Group]
contig_2320	2.8	0.00944	Not found

Ta_S58849094	2.8	0.036562	Not found
contig_1882	2.8	0.026119	Not found
contig_2188	2.8	0.028778	Dynamin-like protein
Ta_S58907153	2.8	0.045212	Post-illumination chlorophyll fluorescence increase protein
Ta_S32591420	2.8	0.026478	C-terminal zinc-finger
Ta_S17988916	2.8	0.00575	Probable atp-dependent rna helicase dhx36-like
contig_1923	2.8	0.012786	Not found
contig_1955	2.8	0.049442	Not found
Ta_S18012554	2.8	0.043176	At5g16250 t21h19_170
Ta_S12880675	2.7	0.019579	Af385697_1 at5g10580 f12b17_70
contig_4145	2.7	0.046526	Not found
Ta_S58886363	2.7	0.013311	Stearoyl-acyl-carrier protein desaturase
Ta_S32628058	2.7	0.014778	Protein mitochondrial-like
Ta_S37846208	2.7	0.015742	Anthocyanidin -o-glucosyltransferase-like
Ta_S58902249	2.7	0.032019	Not found
Ta_S16058277	2.7	0.003438	Cinnamyl alcohol dehydrogenase
contig_4117	2.7	0.028115	Not found
Ta_S58888109	2.7	0.015363	Tpa: nac transcription factor
Ta_S52545220	2.7	0.024114	Chloroplast omega-6 fatty acid desaturase
Ta_S58900624	2.7	0.028533	Serine hydroxymethyltransferase
contig_4744	2.7	0.043444	Probable fructokinase-2
Ta_S58885157	2.7	0.007421	Retrotransposon expressed
Ta_S37889954	2.7	0.038063	Not found
contig_5322	2.7	0.005918	Not found
Ta_S32537167	2.7	0.033359	Not found
Ta_S58861966	2.7	0.044648	Diacylglycerol acyltransferase
Ta_S26027242	2.7	0.026064	Chaperonin 20
contig_5307	2.7	0.01233	PREDICTED: uncharacterized protein LOC100838094 [Brachypodium distachyon]
contig_132	2.7	0.01751	Not found
contig_7151	2.7	0.015553	Not found
contig_347	2.7	0.005414	Not found
Ta_S58863091	2.7	0.022328	Not found

contig_2278	2.7	0.037931	Predicted protein [Hordeum vulgare subsp. Vulgare]
Ta_S17987196	2.7	0.03366	Alpha 3 subunit of 20s proteasome
Ta_S13179831	2.7	0.0085	Not found
contig_179	2.7	0.027104	Choline ethanolamine kinase
Ta_S52543551	2.7	0.010257	Basic region leucine zipper protein
contig_4596	2.7	0.027564	Protein kinase and pp2c-like domain-containing isoform 2
contig_2341	2.6	0.004378	Not found
Ta_S32661131	2.6	0.011714	Wir1b_wheat ame: full=protein wir1b
Ta_S58865460	2.6	0.031304	Aaa family atpase cdc48 subfamily
Ta_S58904389	2.6	0.017919	Clp-like energy-dependent protease
contig_3754	2.6	0.047926	Not found
contig_5733	2.6	0.041474	Not found
Ta_S58855639	2.6	0.037345	Not found
Ta_S58850763	2.6	0.048262	PREDICTED: uncharacterized protein At2g39910-like [Brachypodium distachyon]
Ta_S26022932	2.6	0.035619	Grassy tillers 1
Ta_S52546220	2.6	0.038764	Tonoplast intrinsic
contig_3687	2.6	0.020669	Not found
Ta_S32660720	2.6	0.038125	Not found
Ta_S58902262	2.6	0.00681	Tonoplast intrinsic protein
Ta_S58853104	2.6	0.044161	Probable lrr receptor-like serine threonine-protein kinase at3g47570-like
contig_1468	2.6	0.007515	Not found
contig_3564	2.6	0.028809	Not found
Ta_S58850272	2.5	0.044373	Not found
Ta_S58867334	2.5	0.044755	1 domain containing expressed
contig_2492	2.5	0.048355	Rna polymerase beta partial
contig_1864	2.5	0.029312	Probable atp-dependent rna helicase ythdc2-like
contig_5660	2.5	0.041875	Not found
Ta_S13567817	2.5	0.02858	Rubisco subunit binding-protein alpha subunit
contig_5786	2.5	0.00571	Not found
Ta_S13137675	2.5	0.024318	Not found
Ta_S26018735	2.5	0.033949	Ribosome biogenesis protein bop1 homolog

Ta_S52546131	2.5	0.049751	Not found
contig_1896	2.5	0.003622	Not found
Ta_S12922871	2.5	0.012976	Nucleic acid-binding protein
Ta_S22391033	2.5	0.040161	Ser thr-rich protein t10 in dgcr region
Ta_S18012285	2.5	0.009965	Not found
Ta_S52545881	2.5	0.022684	Anthocyanin 5-aromatic acyltransferase-like
Ta_S24513426	2.5	0.046717	G1-like3 protein
contig_7038	2.5	0.04571	Argininosuccinate lyase
Ta_S58853020	2.5	0.045993	PREDICTED: uncharacterized protein LOC100833596 [Brachypodium distachyon]
Ta_S32541082	2.5	0.039925	Not found
Ta_S13205638	2.4	0.017913	Not found
contig_1250	2.4	0.00939	Not found
contig_4290	2.4	0.006712	Not found
Ta_S13049499	2.4	0.030413	Non-specific lipid-transfer protein at2g13820-like
contig_1177	2.4	0.04838	Not found
contig_5852	2.4	0.00586	Importin subunit beta-1-like
contig_956	2.4	0.028589	Not found
Ta_S12923244	2.4	0.009896	Ribulose -bisphosphate carboxylase activase
contig_6317	2.4	0.035749	Not found
Ta_S13163819	2.4	0.036475	Predicted protein [Hordeum vulgare subsp. Vulgare]
Ta_S37811723	2.4	0.028953	Not found
Ta_S58886850	2.4	0.015067	Apurinic endonuclease-redox protein
Ta_S26020605	2.3	0.003917	Not found
Ta_S58855596	2.3	0.025724	Protein nlrc3-like
Ta_S17987082	2.3	0.020748	Aquaporin tip4-1
Ta_S17890271	2.3	0.039185	H+ ca2+ exchanger
Ta_S26025478	2.3	0.02849	Dehydrin 5
Ta_S52547012	2.3	0.016971	1-aminocyclopropane-1-carboxylate oxidase homolog 1-like
Ta_S17987611	2.3	0.030724	Protein kinase chloroplast
contig_1672	2.3	0.006677	Predicted protein [Hordeum vulgare subsp. Vulgare]
Ta_S58896564	2.3	0.037143	Not found
Ta_S17987213	2.3	0.046563	Rna polymerase beta subunit

contig_3666	2.3	0.025692	Calcium-dependent protein kinase
contig_2386	2.3	0.043744	Type i inositol- -trisphosphate 5-phosphatase cvp2-like
contig_4893	2.3	0.025021	Not found
contig_329	2.3	0.01921	Not found
contig_4	2.3	0.041515	Nadh dehydrogenase subunit 2
contig_6090	2.3	0.041248	Abscisic acid receptor pyl8-like
Ta_S22381224	2.3	0.044987	Potassium transporter 25-like
Ta_S17889976	2.3	0.036853	Chlorophyll a b binding protein
Ta_S50379630	2.3	0.036224	Casp-like protein at3g53850-like
Ta_S13232890	2.3	0.016033	Adp-ribosylation factor
Ta_S22368999	2.3	0.038426	Not found
contig_984	2.2	0.038863	Not found
contig_4802	2.2	0.025676	Ap-2 complex subunit sigma-like
Ta_S17888862	2.2	0.014477	Cysteine-rich repeat secretory protein 55-like
Ta_S58842296	2.2	0.045837	Copine (calcium-dependent phospholipid-binding protein) family
Ta_S35138743	2.2	0.014374	Fasciclin-like arabinogalactan protein 11-like
Ta_S52546553	2.2	0.001782	Subtilisin-like protease
contig_3386	2.2	0.012737	Heavy metal p-type atpase
contig_1812	2.2	0.026186	Not found
contig_264	2.2	0.044341	O-methyltransferase family expressed
Ta_S26019852	2.2	0.033632	Not found
Ta_S58897752	2.2	0.04849	Not found
Ta_S13127316	2.2	0.028718	Predicted protein [Hordeum vulgare subsp. Vulgare]
contig_1333	2.2	0.047689	Nadh-plastoquinone oxidoreductase subunit 4
Ta_S22390999	2.2	0.032546	Not found
Ta_S52542749	2.2	0.015284	Hexokinase 2
contig_300	2.2	0.026097	Kelch motif family protein
Ta_S17988496	2.2	0.002513	Sex determination protein tasselseed 2
Ta_S17989302	2.2	0.042233	Adenylate kinase
Ta_S16203131	2.2	0.046073	Ribosomal protein l29 family protein
Ta_S58884885	2.2	0.041543	Predicted protein [Hordeum vulgare subsp. Vulgare]
Ta_S17890391	2.2	0.031877	Vacuolar sorting receptor 1 expressed
Ta_S58863494	2.1	0.044972	Retrotransposon protein

contig_4909	2.1	0.006443	Not found
Ta_S37822414	2.1	0.034591	Pentatricopeptide repeat-containing protein
Ta_S52544010	2.1	0.045153	3 -n-debenzoyl-2 -deoxytaxol n-benzoyltransferase-like
contig_2559	2.1	0.042846	At1g03060-like partial
Ta_S13110644	2.1	0.019238	Not found
Ta_S17888161	2.1	0.031418	Chlorophyll a-b binding protein of lhcii type iii
Ta_S37752627	2.1	0.006329	Not found
Ta_S26023930	2.1	0.027836	High mobility group b protein 7-like
Ta_S26027971	2.1	0.037103	Calmodulin-like protein 6-like
Ta_S32686907	2.1	0.008878	Not found
contig_6052	2.1	0.042806	Uncharacterized membrane protein at1g16860-like
contig_1648	2.1	0.024846	Isoleucyl-trna cytoplasmic-like
Ta_S53111501	2.1	0.037225	Uncharacterized cdp-alcohol phosphatidyltransferase class-i family protein 3-like
Ta_S12926531	2.1	0.03622	Deoxyuridine 5 -triphosphate nucleotidohydrolase
Ta_S58893991	2.1	0.020919	Receptor-like protein kinase feronia-like
contig_2368	2.1	0.035919	Not found
contig_1504	2.1	0.03768	E3 ubiquitin-protein ligase rnf14-like
contig_1845	2.1	0.008551	Not found
contig_1554	2.1	0.046294	Actin-related protein 2 3 complex subunit 2-like
contig_2934	2.1	0.043022	Transmembrane protein 87b
Ta_S16058462	2.1	0.015165	Tryptophansynthase subunit beta
Ta_S13000024	2.1	0.049558	Homeobox protein hox-c4
contig_1033	2.1	0.009665	Not found
Ta_S18008953	2.1	0.040356	Not found
Ta_S52542397	2.1	0.037953	Rhodanese homology domain-containing protein
Ta_S12977017	2.1	0.034287	Sialyltransferase-like protein
Ta_S26028954	2.1	0.019628	Cysteine-rich receptor-like protein kinase 10-like
Ta_S32566041	2.1	0.035432	Not found
contig_4518	2.1	0.0373	Tbcc domain-containing protein 1-like isoform 2
contig_2912	2.1	0.047699	Not found
Ta_S58885203	2.0	0.013504	Auxin-induced protein pcnt115-like isoform 1
Ta_S52542430	2.0	0.001997	Nitrile-specifier protein 5-like

contig_2364	2.0	0.035622	Not found
Ta_S52541388	2.0	0.017841	Not found
contig_5468	2.0	0.034603	Not found
contig_5536	2.0	0.023919	Not found
Ta_S13109609	2.0	0.009045	Glutathione s-transferase 4-like
Ta_S58849569	2.0	0.041587	Eukaryotic aspartyl protease family expressed
Ta_S58867749	2.0	0.043975	Glutamyl-trna synthetase
contig_4742	2.0	0.049874	Not found
Ta_S13146959	2.0	0.045941	Hypothetical protein [Zea mays]
Ta_S58865230	2.0	0.030608	Protein binding
Ta_S52542697	2.0	0.033319	Hypothetical protein SORBIDRAFT_09g022220 [Sorghum bicolor]
Ta_S13057957	2.0	0.02364	Hydroquinone glucosyltransferase
Ta_S58900435	2.0	0.040822	Cobw domain-containing protein 2-like isoform 2
Ta_S58857859	2.0	0.035993	Tsn1
Ta_S58896684	2.0	0.004458	Thiamine biosynthetic enzyme
contig_2355	2.0	0.040908	Not found
Ta_S52544123	2.0	0.038415	Short chain alcohol
Ta_S17889067	2.0	0.01788	Phosphoenolpyruvate carboxykinase

[a] Fold-change in red indicates down-regulation in colonized wheat roots (CWR); (+)ND not expressed in the N-IWR libraries; (-) ND not expressed in the CWR libraries.
