# Supplementary material for: Dual RNA-seq transcriptional analysis of wheat roots colonized by Azospirillum brasilense reveals up-regulation of nutrient acquisition and cell cycle genes
Source: BMC Genomics. 2014 May 16;15(1):378. doi: 10.1186/1471-2164-15-378 (PMC4042000; doi:10.1186/1471-2164-15-378)
Supplement: Supplementary file 6 — Additional file 6: Table S5A: Overrepresented GO categories of up-regulated Triticum aestivum-ESTs in colonized roots. Table S5B. Overrepresented GO categories of down-regulated Triticum aestivum-ESTs in colonized roots. aFold-change in red indicates down-regulation in colonized wheat roots (CWR). (PDF 116 KB) [file 12864_2013_6083_MOESM6_ESM.pdf]

Table S5A Overrepresented GO categories of up-regulated *Triticum aestivum*-ESTs in colonized roots

Sequence ID	Fold-Change	*p*-value	Sequence Description
		Transport Activity (Molecular Function)	
Ta_S58899343	3.1	0.0037	Metal transporter nramp6-like
Ta_S58895262	2.4	0.0412	Predicted protein [hordeum vulgare subsp. Vulgare]
Ta_S58894107	3.2	0.0245	Amino acid permease
Ta_S58842370	4.0	0.0155	GDP-mannose transporter
Ta_S32626973	2.6	0.0292	Peptide transporter ptr2-like
Ta_S32501583	3.4	0.0168	Conserved hypothetical protein
Ta_S17976596	3.1	0.0089	Sulphate transporter
Ta_S17897607	2.9	0.0182	Major facilitator protein
Ta_S13177057	4.2	0.0091	Nitrate efflux transporter
Ta_S13172731	3.2	0.0469	Cytochrome c oxidase polypeptide vib
Ta_S12971663	2.0	0.0367	Carrier protein
contig_6340	2.1	0.0166	Predicted protein [hordeum vulgare subsp. Vulgare]
contig_6114	3.4	0.0315	Mac perforin domain containing protein
contig_6109	2.2	0.0394	Probable importin subunit beta-4-like
contig_5883	3.6	0.0073	Amino acid permease family expressed
contig_5105	5.0	0.0024	Calcium-transporting ATPase plasma membrane-type-like
contig_4225	2.1	0.0360	Calcium-transporting ATPase endoplasmic reticulum-type-like
contig_2635	2.4	0.0202	ABC transporter c family member 9-like
contig_2239	4.5	0.0009	ABC transporter b family member 20-like
		Enzyme Regulator Activity (Molecular Function)	
Ta_S58886404	2.2	0.0356	Conserved hypothetical protein
Ta_S37759224	3.9	0.0063	Nb-arc domain containing expressed
contig_2743	2.4	0.0372	Disease resistance protein rga4-like
contig_1160	3.1	0.0131	Disease resistance protein rpm1-like

Death – (Biological Process)			
Ta_S32501583	3.4	0.0168	Conserved hypothetical protein
contig_5199	3.6	0.0023	Disease resistance protein rpm1-like
contig_4292	2.9	0.0063	Nb-arc domain containing expressed
contig_395	2.0	0.0430	Disease resistance protein rga4-like
contig_2394	2.9	0.0243	NBS-LRR
Biological Regulation – (Biological Process)			
Ta_S59490000	6.9	0.0465	Myb transcription factor
Ta_S58899343	3.1	0.0037	Metal transporter nramp6-like
Ta_S58891564	7.9	0.0001	Cytochrome p450 liketbp
Ta_S58886404	2.2	0.0356	Tbc domain containing protein
Ta_S58884158	14.0	0.0000	Cytochrome p450 liketbp
Ta_S58855127	2.2	0.0240	Protein far1-related sequence 12-like
Ta_S37811862	2.4	0.0215	DNA binding protein
Ta_S17889461	2.5	0.0275	Sphingoid long-chain bases kinase 1
Ta_S16267847	2.4	0.0402	Hsf-type dna-binding domain containing expressed
Ta_S16212763	2.5	0.0195	Galactose-1-phosphate uridylyltransferase
Ta_S13165985	4.3	0.0275	Auxin-induced protein 15a
Ta_S13124877	2.7	0.0019	Initiation factor
contig_791	2.2	0.0047	Regulator of nonsense transcripts 1 homolog
contig_5777	2.2	0.0345	Beach domain-containing protein lvsa-like
contig_5714	2.6	0.0393	Actin depolymerizing factor 4
contig_4052	2.1	0.0454	Arp2 3 complex 34 kda subunit
contig_4027	13.8	0.0122	Hsf-type dna-binding domain containing expressed
contig_3699	2.8	0.0253	Histidine kinase- dna gyrase b- and hsp90-like atpase family protein
contig_3502	2.4	0.0308	Vacuolar protein sorting 13c
contig_31	2.6	0.0362	Vacuolar protein sorting 13c
contig_2914	2.2	0.0354	Protease do-like 7-like
contig_2743	2.4	0.0372	Tbc domain containing protein

contig_2730	2.3	0.0269	Chd3-type chromatin-remodeling factor pickle-like
contig_2382	2.4	0.0459	Transcription factor bhlh128
contig_1160	3.1	0.0131	Brefeldin a-inhibited guanine nucleotide-exchange protein 1-like
		Extracellular Region – (Cellular Component)	
Ta_S37759224	3.9	0.0063	Bowman-birk trypsin inhibitor-like protein
Ta_S20999571	2.3	0.0303	Probable xyloglucan endotransglucosylase hydrolase protein 8-like
Ta_S17893420	2.4	0.0084	Beta-expansin 1a expressed
Ta_S12902351	5.7	0.0409	Endo-beta-glucanase
contig_3741	3.7	0.0003	Beta-expansin 2 precursor
		Macromolecular Complex – (Cellular Component)	
Ta_S58894413	2.7	0.0358	6-phosphofructokinase 4
Ta_S58861971	-	0.0308	Uncharacterized protein [Brachypodium distachyon]
Ta_S58854607	59.8	0.0012	Replication factor c subunit
Ta_S43485972	2.9	0.0101	Protein phosphatase 2c
Ta_S32659341	2.8	0.0304	Ribosomal protein s12
Ta_S17880905	2.3	0.0483	50s ribosomal protein l33
Ta_S13172731	3.2	0.0469	Cytochrome c oxidase polypeptide vib
Ta_S13148233	2.3	0.0155	60s ribosomal protein l34
Ta_S13146836	3.3	0.0158	60s ribosomal protein l7-1
Ta_S13144009	3.6	0.0458	60s ribosomal protein l34
contig_6109	2.2	0.0394	Probable importin subunit beta-4-like
contig_5480	4.3	0.0015	DNA repair protein rad50
contig_3896	4.9	0.0231	Atp synthase f0 subunit 6
contig_3292	4.7	0.0016	Ribonucleotide reductase
contig_2968	2.2	0.0362	Eukaryotic translation initiation factor 3 subunit c-like
contig_2943	2.7	0.0173	NADH-ubiquinone oxidoreductase subunit
contig_2923	2.4	0.0329	Callose synthase
contig_1395	2.0	0.0331	Kinesin heavy chain

contig_1285	3.3	0.0489	Microtubule organization protein

Table S5B Overrepresented GO categories of down-regulated *Triticum aestivum*-ESTs

**Sequence ID**	**Fold-Change** [a]	***p*-value**	**Sequence Description**
Nucleic Acid Biding Transcriptional Regulator Activity – (Molecular Function)			
Ta_S58889807	6.2	0.0394	Global transcription factor group
Ta_S58855596	2.3	0.0257	Protein nlrc3-like
Ta_S52543551	2.7	0.0103	Basic region leucine zipper protein
Ta_S35718003	3.3	0.0344	Cbf protein 4
Ta_S26023930	2.1	0.0278	High mobility group b protein 7-like
Ta_S26022932	2.6	0.0356	Grassy tillers 1
Ta_S13000024	2.1	0.0496	Homeobox protein hox-c4
Ta_S12923244	2.4	0.0099	Ribulose-bisphosphate carboxylase activase
contig_179	2.7	0.0271	Choline ethanolamine kinase
Biological Regulation – (Biological Process)			
Ta_S59490003	4.9	0.0402	R2R3-Myb transcription factor
Ta_S58889807	6.2	0.0394	Global transcription factor group
Ta_S58888286	6.4	0.0027	Protein chloroplast import apparatus 2-like
Ta_S58886850	2.4	0.0151	Apurinic endonuclease-redox protein
Ta_S58869803	4.5	0.0224	Something about silencing protein
Ta_S58863091	1.4	0.0223	Negative regulation of translation N-glycosylase activity
Ta_S58855596	2.3	0.0257	Protein nlrc3-like
Ta_S52545220	2.7	0.0241	Chloroplast omega-6 fatty acid desaturase
Ta_S52544123	2.0	0.0384	Short chain alcohol
Ta_S52543551	2.7	0.0103	Basic region leucine zipper protein
Ta_S35718003	3.3	0.0344	Cbf protein 4
Ta_S26022932	2.6	0.0356	Grassy tillers 1
Ta_S17057489	3.6	0.0121	Auxin response factor 3-like

Ta_S13232890	2.3	0.0160	Adp-ribosylation factor
Ta_S13051479	3.4	0.0261	Serine threonine-protein kinase afc1
Ta_S13014352	7.8	0.0170	Protein suppressor of gene silencing-like protein
Ta_S13011770	3.9	0.0160	Response regulator receiver domain
Ta_S13000024	2.1	0.0496	Homeobox protein hox-c4
contig_6115	5.5	0.0118	Cytochrome c biogenesis protein precursor
contig_3567	3.1	0.0466	Histidine kinase 1
contig_2559	2.1	0.0428	At1g03060-like partial
contig_179	2.7	0.0271	Choline ethanolamine kinase
contig_1554	2.1	0.0463	Actin-related protein 2 3 complex subunit 2-like
Extracellular Region – (Cellular Component)			
Ta_S53281796	4.6	0.0045	Alpha expansin [Triticum aestivum]
Ta_S52546553	2.2	0.0018	Subtilisin-like protease
Ta_S52543823	4.4	0.0147	Glycolate oxidase
Ta_S26027242	2.7	0.0261	Chaperonin 20
Ta_S13567817	2.5	0.0286	Rubisco subunit binding-protein alpha subunit
contig_189	3.6	0.0028	Hydroxypyruvate reductase
Macromolecular Complex – (Cellular Component)			
Ta_S58904389	2.6	0.0179	Clp-like energy-dependent protease
Ta_S58900106	3.8	0.0361	Light-harvesting complex I chlorophyll a b binding protein 3
Ta_S58889807	6.2	0.0394	Global transcription factor group
Ta_S52541238	3.0	0.0367	Unknown protein (partial protein)
Ta_S26026252	2.8	0.0275	50s ribosomal protein l9
Ta_S26025255	5.4	0.0120	Photosystem II reaction center w protein
Ta_S26022483	3.8	0.0047	Mrna turnover protein 4 homolog
Ta_S17987196	2.7	0.0337	Alpha 3 subunit of 20s proteasome
Ta_S17889976	2.3	0.0369	Chlorophyll a b binding protein
Ta_S17888161	2.1	0.0314	Chlorophyll a-b binding protein of LHCII type III
Ta_S16203131	2.2	0.0461	Ribosomal protein l29 family protein
Ta_S13567817	2.5	0.0286	Rubisco subunit binding-protein alpha subunit

Ta_S12894932	5.9	0.0034	Vip3 protein (predicted protein [Hordeum vulgare subsp. Vulgare])
contig_6247	3.1	0.0317	Ubiquitin-protein pub49
contig_5972	3.4	0.0369	Kinesin-like protein
contig_1554	2.1	0.0463	Actin-related protein 2 3 complex subunit 2-like

[a] Fold-change in red indicates down-regulation in colonized wheat roots (CWR).
