# Supplementary material for: Dual RNA-seq transcriptional analysis of wheat roots colonized by Azospirillum brasilense reveals up-regulation of nutrient acquisition and cell cycle genes
Source: BMC Genomics. 2014 May 16;15(1):378. doi: 10.1186/1471-2164-15-378 (PMC4042000; doi:10.1186/1471-2164-15-378)
Supplement: Supplementary file 7 — Additional file 7: Figure S2: Scatter plot: RNA-seq vs. RT-qPCR. RNA-seq and RT-qPCR relative level of expression are presented as log2 (fold-change). Triticum aestivum genes presented in the scatter plot: TaS_37759224**, TaS_52545880**, contig_4027***, TaS_52541981*, TaS_22370925*, TaS_37846208*, TaS_17890143*, TaS_26026646*, TaS552541449** and contig_2766**. RNA-seq data represent biological duplicates of colonized and non-inoculated wheat roots. RT-qPCR data represent biological duplicates of colonized and non-inoculated wheat roots, obtained independently from the RNA-seq samples, and each sample was run in triplicate. Significant differences in the RT-qPCR (*p < 0.05, **p < 0.01, and ***p < 0.001) were determined using one-tailed t-test. Pearson coefficient correlation between RNA-seq and RT-qPCR was > 0.97, p-value < 0.0001. (PDF 8 KB) [file 12864_2013_6083_MOESM7_ESM.pdf]

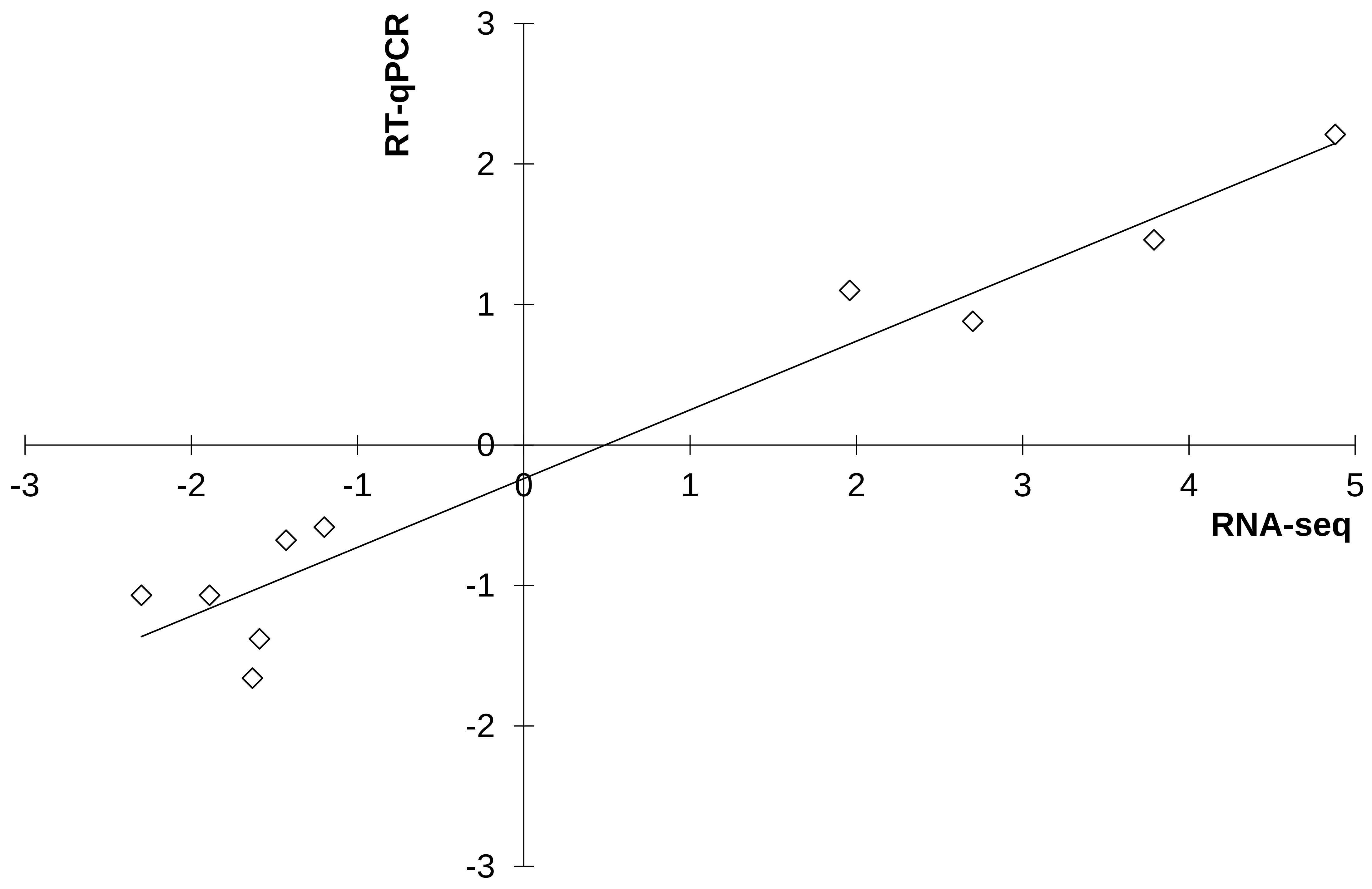

Figure S2 Scatter plot: RNA-seq vs. RT-qPCR

RNA-seq and RT-qPCR relative level of expression are presented as log2fold-change

Triticum aestivum genes presented in the scatter plot: TaS_37759224**, TaS_52545880**, contig_4027***, TaS_52541981*, TaS_22370925*, TaS_37846208*, TaS_17890143*, TaS_26026646*, TaS552541449** and contig_2766**. RNA-seq data represent biological duplicates of colonized and non-inoculated wheat roots. RT-qPCR data represent biological duplicates of colonized and non-inoculated wheat roots, obtained independently from RNA-seq samples, and each sample was run in triplicate. Significant differences in the RT-qPCR (*p < 0.05, ** p < 0.01, and *** p < 0.001) were determined using one-tailed t-test. Pearson coefficient correlation between RNA-seq and qRT-PCR was >0.97, p-value<0.0001.
