# Supplementary material for: Dual RNA-seq transcriptional analysis of wheat roots colonized by Azospirillum brasilense reveals up-regulation of nutrient acquisition and cell cycle genes
Source: BMC Genomics. 2014 May 16;15(1):378. doi: 10.1186/1471-2164-15-378 (PMC4042000; doi:10.1186/1471-2164-15-378)
Supplement: Supplementary file 8 — Additional file 8: Table S6: Triticum aestivum MADs-Box expressed-ESTs. aFold-change in red indicates lower level of expression in colonized wheat roots (CWR). (PDF 15 KB) [file 12864_2013_6083_MOESM8_ESM.pdf]

Table S6 *Triticum aestivum* MADs-Box expressed-ESTs

Sequence ID	Fold-Change	*p*-value	Sequence Description
Ta_S32520254	2.7	0.1381	MADs-box transcription factor (TaAGL33-like)
Ta_S13147416	1.4	0.4905	MADs box-like protein
Ta_S41658047	1.3	0.8285	MIKC-type mads-box transcription factor (WM30B-like)
Ta_S26028956	1.2	0.7430	MIKC-type mads-box transcription factor (WM12B-like)
Ta_S33259699	1.2	0.4477	MIKC-type mads-box transcription factor (WM14B-like)
Ta_S26028946	1.1	0.7267	MADs-box transcription factor (TaAGL33-like)
contig_3667	1.0	0.5672	MADs box interactor-like
Ta_S58889628	1.0	0.7981	MADs box interactor-like
Ta_S52542334	1.1	0.8974	MADs box-like protein
Ta_S39875454	1.2	0.7417	MADs-box transcription factor (TaAGL4-like)
Ta_S32896805	1.3	0.7167	MADs-box transcription factor (TaAGL32-like)
Ta_S33259701	1.4	0.6196	MADs-box transcription factor (TaAGL33-like)
Ta_S37823145	1.4	0.3463	MADs-box transcription factor 26
Ta_S41658057	1.4	0.1931	MIKC-type mads-box transcription factor (WM22B-like)
Ta_S41658042	1.6	0.1974	MIKC-type mads-box transcription factor (WM32B-like)
Ta_S41658082	1.7	0.2603	MIKC-type mads-box transcription factor (WM3B-like)
Ta_S26028737	1.7	0.5153	MADs box-like protein

[a] Fold-change in red indicates lower level of expression in colonized wheat roots (CWR).
