# Supplementary material for: Dual RNA-seq transcriptional analysis of wheat roots colonized by Azospirillum brasilense reveals up-regulation of nutrient acquisition and cell cycle genes
Source: BMC Genomics. 2014 May 16;15(1):378. doi: 10.1186/1471-2164-15-378 (PMC4042000; doi:10.1186/1471-2164-15-378)
Supplement: Supplementary file 9 — Additional file 9: Table S7: Expressed ORFs of Azospirillum brasilense strain FP2 colonizing Triticum aetivum seedling roots. (PDF 36 KB) [file 12864_2013_6083_MOESM9_ESM.pdf]

Table S7 Expressed ORFs of *Azospirillum brasilense* strain FP2 colonizing *Triticum aetivum* seedling roots

ORF	Gene name	CWR coverage	Protein descriptions
Genes of Azospirillum brasilense involved in nitrogen fixation			
0003.0824	*amtB*	2.2	Ammonium transporter
0001.1395	*glnA*	9.6	Glutamine synthetase
0001.0450	*glnB*	7.8	Nitrogen regulatory protein pII
0001.1150	*nifH*	1.5	Nitrogenase reductase
0001.1149	*nifD*	1.4	Nitrogenase molybdenum-iron protein alpha chain
0001.1148	*nifK*	1.6	Nitrogenase molybdenum-iron protein beta chain
Genes of Azospirillum brasilense involved in adaptation processes			
0007.0064		6.7	Hypothetical calcium-binding protein
0004.0099		1.8	Hypothetical hemolysin-type calcium-binding region
0003.0744		1.6	Putative hemolysin-type calcium-binding region
0001.2030	*narL-like*	30.2	Two-component response regulator
0005.0680	*narL-like*	6.9	Two-component response regulator
0001.2448	*narL-like*	3.7	Two-component response regulator
0002.0515		39.5	Putative transcriptional regulator, Crp/Fnr family
0001.1830		1.8	Crp/Fnr family transcriptional regulator
0001.1948		1.1	Crp/Fnr family transcriptional regulator
0002.0162		12.8	Superoxide dismutase
0003.0751	*phaC*	5.7	Poly(3-hydroxyalkanoate) synthetase
0004.0441	*phbC*	1.5	Poly-beta-hydroxybutyrate polymerase
0005.0595	*phbC*	1.3	Poly-beta-hydroxybutyrate polymerase
0001.1620		12.8	Phasin protein
0001.1052		9.6	Phasin protein
0001.1053		1.0	Phasin protein
0002.0156		1.0	Phasin protein
Genes of Azospirillum brasilense involved in chemotaxis, adhesion and biofilm formation			
0002.1124	*xseB*	3.8	Exodeoxyribonuclease VII small subunit

0003.0134	*exoP*	9.1	Succinoglycan biosynthesis transport protein
0002.0842	*expY*	6.1	Undecaprenyl-phosphate galactose phosphotransferase activator
0007.0020	*exoB*	1.0	UDP-glucose 4-epimerase
0003.0288		5.7	UDP-glucose 4-epimerase
0002.0519	*galE*	1.3	UDP-glucose 4-epimerase
0001.1918	*glmS*	2.1	Glucosamine-fructose-6-phosphate aminotransferase
0005.0494		2.6	Glucose-methanol-choline oxidoreductase
0003.0290		4.8	Putative glycosyl transferase
0001.1795		1.1	Putative glycosyl transferase
0002.0840		1.6	Putative glycosyl transferase
0002.0849		4.6	Putative glycosyl transferase
0001.1208		1.6	Putative glycosyl transferase
0003.0140		6.2	Glycosyl transferase
0002.0838		2.9	Putative glycosyl transferase
0002.0839		1.6	Putative glycosyl transferase
0001.1950		2.2	Putative glycosyl transferase
0001.2661		2.1	Putative glycosyl transferase
0007.0094		2.7	Putative glycosyl transferase
0002.1398	*cheY-like*	29.4	Two-component response regulator
0005.0410	*sbpA*	67.8	Sugar biding protein
0005.0410	*gbpR*	67.8	HTH-type transcriptional regulator
0005.0411	*gguA*	16.5	Monosaccharide ABC transporter
0005.0412	*gguB*	18.5	Monosaccharide ABC transporter
0005.0408	*ytfQ-like*	31.0	Sugar ABC transporter
0005.0377	*galU*	3.6	UDP-glucose-1-phosphate uridylyltransferase (biosynthesis and degradation of surface polysaccharides and lipopolysaccharides)

0003.0953		5.9	Putative sugar ABC transporter; substrate-binding periplasmic component
0001.2285		6.2	Putative polysaccharide export protein
0003.0301		1.9	Putative lipopolysaccharide biosynthesis protein
0003.0287		8.0	UDP-glucose 6-dehydrogenase
0003.0135		14.7	UDP-glucose GDP-mannose dehydrogenase
0001.1919	*glmU*	2.6	UDP-n-acetylglucosamine pyrophosphorylase
0005.0407	*rbsA-like*	4.8	Sugar ABC transporter, ATP-binding component
0005.0405	*rbsC-like*	3.8	Sugar ABC transporter, permease component
0005.0406	*rbsC-like*	3.1	Sugar ABC transporter, permease component
0003.0292		5.8	Putative bacterial sugar transferase family protein
