# Supplementary material for: Dual RNA-seq transcriptional analysis of wheat roots colonized by Azospirillum brasilense reveals up-regulation of nutrient acquisition and cell cycle genes
Source: BMC Genomics. 2014 May 16;15(1):378. doi: 10.1186/1471-2164-15-378 (PMC4042000; doi:10.1186/1471-2164-15-378)
Supplement: Supplementary file 10 — Additional file 10: Table S8: Host response found during plant-microbe interaction: Defense mechanism, hormone imbalances and secretion of phytochemicals. aFold-change in red indicates down-regulation in colonized wheat roots (CWR); (+) ND not expressed in the N-IWR libraries; (−) ND not expressed in the CWR libraries. (PDF 30 KB) [file 12864_2013_6083_MOESM10_ESM.pdf]

Table S8 Host response found during plant-microbe interaction: Defense mechanism, hormone imbalances and secretion of phytochemicals

Sequence ID	Fold Change[a]	*p-value*	Sequence description
			Defense mechanism
Ta_S58840665	(+) ND	0.0449	Hypothetical protein
Ta_S58884106	14.9	0.0004	Disease resistance protein (rpm1-like)
Ta_S12902351	5.7	0.0409	Endo-beta-glucanase
Ta_S37759224	3.9	0.0063	Bowman-birk trypsin inhibitor-like protein
contig_2770	3.7	0.0001	Beta-amyrin synthase
contig_5199	3.6	0.0023	Disease resistance protein (rpm1-like)
Ta_S58861829	3.4	0.0117	Beta-glucosidase 5-like isoform 2
Ta_S58851000	2.9	0.0171	LRR receptor serine threonine-protein kinase at3g47570-like
contig_4292	2.9	0.0063	NB-arc domain containing expressed
contig_2394	2.9	0.0243	NBS-LRR partial
contig_1142	2.6	0.0477	Blight resistance protein
Ta_S58861380	2.5	0.0453	Serine threonine-protein kinase (rks1-like)
Ta_S22378208	2.5	0.0314	Beta-amyrin synthase
Ta_S26028472	2.3	0.0435	NBS-LRR disease resistance protein homologue
contig_395	2.0	0.0430	Disease resistance protein rga4-like
Ta_S32501583	3.4	0.0168	Hypothetical protein
Ta_S58857859	2.0	0.0360	*tsn1*
Ta_S52546553	2.2	0.0018	Subtilisin-like protease
contig_6090	2.3	0.0412	Abscisic acid receptor (pyl8-like)
Ta_S58853104	2.6	0.0442	LRR receptor-like serine threonine-protein kinase (at3g47570-like)
contig_4596	2.7	0.0276	Protein kinase (pp2c-like)
contig_2829	3.3	0.0165	Disease resistance protein rpp13-like
contig_667	3.4	0.0073	Beta-glucosidase 3 precursor
Ta_S37773901	3.9	0.0210	Cytochrome p450

Ta_S32602541	4.3	0.0356	WRKY45-like transcription factor
contig_387	4.5	0.0151	Peroxidase 1 precursor
Ta_S16246857	7.3	0.0062	Disease resistance protein rpm1-like
Ta_S58912535	(-)ND	0.0333	WAK1 - receptor-like cytoplasmic kinase (-rlck)
Defense mechanism: Heat shock proteins			
Ta_S52544833	80.0	0.0375	Small heat shock protein
Ta_S16236735	76.4	0.0196	Heat-shock protein
Ta_S16229254	48.9	0.0489	Heat shock protein
Ta_S52541981	29.4	0.0198	Heat shock
Ta_S58858619	19.9	0.0292	Low molecular weight heat shock protein
Ta_S22373503	14.5	0.0353	HSF-type DNA-binding domain
contig_4027	13.8	0.0122	HSF-type DNA-binding domain
Ta_S32507710	6.2	0.0000	Heat shock cognate 70 kda
Ta_S16267847	2.4	0.0402	HSF-type DNA-binding domain containing expressed
contig_3699	2.8	0.0253	HSP90-like ATPase family protein
Hormone imbalances			
Ta_S13165985	4.3	0.0275	Auxin induced protein SAUR (small auxin RNA)
Ta_S17057489	3.6	0.0121	ETTIN/ARF3
Ta_S58885203	2.0	0.0135	Aldo-keto-reductase auxin-induced
Ta_S52541449	3.1	0.0154	
Secretion of phytochemicals			
Ta_S58884786	5.0	0.0349	Flavonol 3-sulfotransferase
Ta_S37854435	11.4	0.0006	AnthoCyanidin -o-glucosyltransferase-like
Ta_S37846208	2.7	0.0157	AnthoCyanidin -o-glucosyltransferase-like

[a] Fold-change in red indicates down-regulation in colonized wheat roots (CWR); (+)ND not expressed in the N-IWR libraries; (-) ND not expressed in the CWR libraries.
