# Supplementary material for: Dual RNA-seq transcriptional analysis of wheat roots colonized by Azospirillum brasilense reveals up-regulation of nutrient acquisition and cell cycle genes
Source: BMC Genomics. 2014 May 16;15(1):378. doi: 10.1186/1471-2164-15-378 (PMC4042000; doi:10.1186/1471-2164-15-378)
Supplement: Supplementary file 11 — Additional file 11: Table S9: ESTs of Triticum aestivum encoding transporters. aFold-change in red indicates lower level of expression in colonized wheat roots (CWR); (+) ND not expressed in the N-IWR libraries; Up-regulated, Down-regulated and Expressed ESTs are shading in red, blue and yellow respectively. (PDF 333 KB) [file 12864_2013_6083_MOESM11_ESM.pdf]

Table S9 ESTs of *Triticum aestivum* encoding transporters

Sequence ID	Fold-change	*p-value*	Sequence description
			Up-regulated ESTs (fold change>2, p-value< 0.05)
contig_2239	4.5	0.0009	ABC transporter b family member 20-like
Ta_S13177057	4.2	0.0091	Nitrate transmembrane transporter naxt1-like
Ta_S58842370	4.0	0.0155	GDP-mannose transporter
contig_5883	3.6	0.0073	Amino acid permease family expressed
Ta_S58899343	3.1	0.0037	Metal transporter nramp6-like
Ta_S17976596	3.1	0.0089	Sulphate transporter
Ta_S32626973	2.6	0.0292	Peptide transporter (ptr2-like)
Ta_S17979451	2.6	0.0235	Magnesium transporter nipa2
contig_2635	2.4	0.0202	ABC transporter c family member 9-like
			Down-regulated ESTs (fold change>2, p-value< 0.05)
contig_6543	4.2	0.0247	ABC transporter g family member 16-like
Ta_S58884172	3.8	0.0190	Sugar transporter family expressed
Ta_S22381224	2.3	0.0450	Potassium transporter 25-like
			Expressed ESTs
Ta_S58863349			ABC transporter
Ta_S16058169			Ammonium transporter amt2
contig_5490			ABC transporter c family member 9-like
contig_3974			Peptide transporter
Ta_S26020880			Sugar transporter family expressed
Ta_S13110775			Amino acid transporter a1
Ta_S22371202			Cation efflux family protein
contig_4243			ABC transporter d family member
Ta_S17979060			Metal transporter nramp5-like
contig_7128			Peptide transporter expressed
contig_3978			Peptide transporter ptr2-like
Ta_S17985880			Peptide transporter ptr2-like
contig_6592			ABC transporter b family member 20-like
Ta_S52543465			Udp-galactose transporter 3
contig_6112			GDP-mannose transporter gonst3-like
contig_1162			Magnesium transporter nipa2-like

Ta_S58867287	Multidrug resistance protein ABC transporter family
contig_3999	ABC transporter c family member 10-like isoform 2
Ta_S13022660	Iron inhibited ABC transporter 2
Ta_S58840467	ABC transporter f family member 4-like
Ta_S32635605	Carbohydrate transporter sugar porter transporter
contig_809	Na^+ H^+ antiporter
contig_2773	Pdr-type ABC transporter-like
Ta_S52543393	Udp-galactose transporter 6
Ta_S17978275	Amino acid transporter
Ta_S58896279	Mate efflux
contig_3156	Mate efflux family protein alf5
Ta_S13050794	Iron-phytosiderophore transporter protein yellow stripe 1
Ta_S58897641	Plastidic glucose transporter 4
contig_3428	Solute carrier family facilitated glucose transporter member 8
Ta_S58899329	Pdr-like ABC transporter
Ta_S13136892	Amino acid transporter
Ta_S58864202	Pdr-like ABC transporter
Ta_S18943257	Ammonium transporter amt1.1
Ta_S19223739	Plasma-membrane choline transporter family protein
Ta_S37809725	Phosphate transporter
contig_2754	Xanthine uracil vitamin c partial
Ta_S58853003	Nucleotide sugar transporter-like protein
contig_5977	Uncharacterized transporter ybr287w-like
Ta_S58889324	Na^+ H^+ antiporter
Ta_S37946965	Proton-dependent oligopeptide or low-affinity nitrate partial
Ta_S17981933	At1est8-like partial
contig_4846	Plastidic glucose transporter 4
Ta_S52545340	Bidirectional sugar transporter n3-like
contig_1260	At3g28860-like partial
Ta_S37854107	Potassium transporter
Ta_S58852201	Nramp transporter
Ta_S58895683	Nitrate transporter -like
contig_4945	ABC transporter d family member 1-like

Ta_S13126051	Pdr-like ABC transporter
Ta_S52545695	Udp-galactose transporter 2-like
contig_1249	Solute carrier family facilitated glucose transporter member 8
Ta_S17890420	Proline transporter 1
Ta_S22496174	Na^{+} H^{+} antiporter
contig_3993	Pdr-like ABC transporter
contig_555	Equilibrative nucleoside transporter
contig_3830	ABC transporter c family member 10-like isoform 2
contig_2332	Multidrug pheromone mdr ABC transporter family
Ta_S12985419	Pdr-like ABC transporter
Ta_S58842340	Multidrug resistance protein ABC transporter family
Ta_S13010915	Hypothetical Protein
contig_5119	Peptide transporter 1
contig_5580	ABC transporter b family member 20-like
contig_6100	Gdp-mannose transporter gonst3-like
Ta_S12922751	General substrate transporter-like protein
contig_1208	ABC transporter d family member
Ta_S32585634	Mdr-like ABC transporter
contig_1348	Pdr-like ABC transporter
contig_181	Magnesium transporter nipa2
Ta_S58897308	ABC transporter d family member 1
Ta_S13021939	Sulphate transporter
Ta_S58861949	Magnesium transporter nipa2-like
Ta_S50389128	Peptide transporter ptr2-like
Ta_S58869513	Multidrug resistance protein ABC transporter family
Ta_S17987431	Potassium transporter
Ta_S52542003	Peptide transporter ptr2-like
Ta_S58869511	ABC transporter c family member 5-like
Ta_S58866161	K^{+} efflux antiporter 6-like
Ta_S52541510	Metal tolerance protein
Ta_S16057927	ABC transporter b family member 4-like
contig_6441	Pdr-like ABC transporter
Ta_S17988485	Aluminum activated citrate transporter

Ta_S16057986	Mate efflux family protein 7-like
Ta_S13256283	Amino acid transporter
contig_1452	Aluminum-activated citrate transporter
Ta_S16058470	Zinc transporter
contig_2876	D-xylose-proton symporter-like 2
Ta_S58888183	Yellow stripe-like transporter 12
Ta_S37802999	Mate efflux family expressed
contig_4261	ABC transporter family expressed
Ta_S58860395	Cation-chloride cotransporter 1-like
Ta_S17989018	ABC transporter family protein
Ta_S58861424	Vacuolar amino acid transporter 1-like
contig_4409	Pdr-type ABC transporter 9
Ta_S13058142	ABC transporter c family member 10-like isoform 2
Ta_S12971892	Gdp-mannose transporter gonst3-like
Ta_S58858831	ABC transporter a family member 7-like
Ta_S52543473	Oligopeptide transporter 7-like
Ta_S58889388	ABC transporter family expressed
Ta_S26027328	Peptide transporter ptr2-like
Ta_S19225168	Cation-chloride cotransporter
Ta_S58843363	Potassium transporter 11-like
contig_6616	Pdr-type ABC transporter-like
contig_7133	Proton-dependent oligopeptide transport family protein
Ta_S32507418	Ammonium transporter
contig_279	ABC transporter c family member 10-like isoform 2
Ta_S13141198	ABC transporter c family member 3-like
Ta_S32531820	ABC transporter g family member 28-like
contig_5109	ABC transporter a family member 4
Ta_S58863047	Mitochondrial phosphate transporter
contig_5629	ABC transporter d family member
Ta_S50378052	Lysine histidine transporter
Ta_S17889376	Vacuolar amino acid transporter 1-like
contig_4051	ABC transporter c family member 10-like isoform 2
contig_4341	Peptide transporter ptr2-like

Ta_S22382486	Pdr-like ABC transporter
Ta_S17889510	Transparent testa 12 protein
Ta_S58851172	Potassium transporter
Ta_S16058073	ABC transporter c family member 3-like
Ta_S58852047	ABC transporter b family member
Ta_S52544358	ABC transporter like protein
Ta_S52544007	Zip-like zinc transporter
contig_5727	Mdr-like ABC transporter
Ta_S52542099	Mate efflux family protein alf5-like
Ta_S17989006	Nodulin 21 -like transporter protein
Ta_S58857667	Pdr-like ABC transporter
Ta_S22383057	Peptide transporter ptr2-like
contig_3530	Pdr-like ABC transporter
Ta_S26027462	Membrane magnesium transporter 1-like
Ta_S22385862	Vacuolar iron transporter-like protein
Ta_S16058124	Ammonium transporter
contig_3544	At5g19640-like partial
Ta_S22370841	Mn-specific cation diffusion facilitator transporter
Ta_S58843245	Auxin efflux carrier family protein
Ta_S58897736	Mate efflux family protein 5-like
Ta_S58860384	Cation-chloride cotransporter 1-like
Ta_S52542689	Peptide transporter
Ta_S52545860	Solute carrier family facilitated glucose transporter member 8
Ta_S58862919	ABC transporter c family member 10-like isoform 1
Ta_S58893800	Potassium transporter 7-like
Ta_S52543797	Multidrug pheromone mdr ABC transporter family
Ta_S58868699	Magnesium transporter mrs2-4-like
Ta_S58851485	Mrp-like ABC transporter
contig_3705	Gdp-mannose transporter gonst3-like
Ta_S20306454	Peptide transporter ptr2-like
Ta_S16258294	ABC transporter d family member
contig_4293	Major facilitator superfamily expressed
Ta_S58852795	Plasma-membrane choline transporter-like protein

contig_3857	D-xylose-proton symporter-like 2
Ta_S17985842	Auxin efflux carrier
contig_401	ABC transporter c family member 10-like
Ta_S22391325	Zinc transporter ztp29-like
Ta_S58881750	Sulfate transporter
Ta_S52542806	Integral membrane transporter family protein
Ta_S52546225	Nucleobase-ascorbate transporter 6-like
Ta_S52543198	Zinc transporter
Ta_S58884802	Pot family expressed
contig_4887	ABC transporter g family member 28-like
Ta_S58882139	ABC transporter b family member 20-like
Ta_S58889949	Iron-phytosiderophore transporter protein yellow stripe 1
Ta_S58890717	Peptide transporter ptr2-b
contig_4808	ABC transporter c family member 8-like
Ta_S58870065	General substrate transporter-like protein
Ta_S58862614	ABC transporter b family member mitochondrial-like
Ta_S17865479	ABC transporter c family member 3-like
contig_833	ABC transporter b family member 19-like
Ta_S52543740	ABC transporter family protein
contig_3504	Pdr-like ABC transporter
contig_5379	Potasium transporter
contig_6737	Nodulin 21 -like transporter protein
Ta_S12923503	ABC transporter b family member 11-like
Ta_S58899651	Hexose transporter
Ta_S13173768	Mate efflux family protein alf5-like
Ta_S18943256	Ammonium transporter
contig_5770	Carbohydrate transporter sugar porter transporter
contig_2118	Lysine histidine transporter 2-like
Ta_S58863243	ABC transporter i family member chloroplastic-like
Ta_S58884915	Magnesium transporter nipa2-like
Ta_S12923309	Na^+ H^+ antiporter
Ta_S58898758	ABC transporter family protein
Ta_S58856407	Potassium transporter 7-like

Ta_S25791176	Na^+ H^+ antiporter
contig_4378	Probable metal-nicotianamine transporter ysl7-like
Ta_S58883208	Bidirectional sugar transporter sweet13-like
Ta_S50371846	Pdr-type ABC transporter
Ta_S58906099	ABC transporter c family member 8-like
Ta_S52543415	Metal transporter nramp3-like
Ta_S52542983	Magnesium transporter nipa2
Ta_S58863315	Bile acid transporter 5
Ta_S37760734	Mate efflux family protein
contig_3540	Peptide transporter ptr2-like
Ta_S16218736	Adenosine 3 -phospho 5 -phosphosulfate transporter 1-like
contig_5035	Cytochrome c biogenesis c
Ta_S52546797	Probable peptide nitrate transporter at1g59740-like
contig_3765	Sucrose transporter
Ta_S58844672	Metal transporter nramp6-like
contig_794	Aluminum-activated malate transporter 9-like
contig_3703	Equilibrative nucleoside transporter 4-like
Ta_S17977242	ABC transporter i family member 17
Ta_S58896318	Potassium transporter
Ta_S58697407	High-affinity potassium transporter
Ta_S17880402	Integral membrane protein
contig_4657	Plastidic glucose transporter 4-like
Ta_S17898000	Sugar transporter
contig_3468	Equilibrative nucleoside transporter
Ta_S52543186	Peptide transporter ptr2-like
Ta_S52546866	Inositol transporter 1
contig_5750	ABC transporter g family member 28-like
Ta_S52546249	Cationic amino acid transporter
Ta_S17977147	Polyol transporter 5-like
Ta_S52546724	Plastidic glucose transporter 4
Ta_S58893716	Sucrose transporter
Ta_S16058493	Auxin efflux carrier component
Ta_S58902373	ABC transporter c family member 2-like

contig_2710	ABC transporter c family member 10-like isoform 2
Ta_S52543655	Nucleobase-ascorbate transporter 6-like
Ta_S58860060	ABC transporter family expressed
Ta_S52543040	Probable metal-nicotianamine transporter ysl5-like
Ta_S58896152	Pdr-like ABC transporter
Ta_S52546483	K^+ efflux antiporter chloroplastic-like
Ta_S58891022	ABC transporter a family member 7-like
contig_5476	Endoplasmic reticulum vesicle transporter protein
Ta_S58897309	ABC transporter d family member 1
Ta_S58885850	Oligopeptide transporter
Ta_S58909002	ABC transporter c family member 10-like isoform 1
contig_1481	Solute carrier family facilitated glucose transporter member 8
Ta_S37805390	Monosaccharide transporter 1
Ta_S52542911	Organic anion transporter
Ta_S58898904	Uncharacterized transporter sll0355-like
contig_398	ABC transporter b family member 19-like
Ta_S58900755	Amino acid transporter
Ta_S16227814	Auxin efflux carrier
Ta_S58853206	Oligopeptide partial
Ta_S17984911	Mate efflux family protein 5-like
Ta_S58850992	Lysine histidine transporter
Ta_S52543924	Major facilitator protein
Ta_S22388588	Zinc transporter ztp29-like
contig_4848	Cytochrome c biogenesis b
contig_5671	Yellow stripe-like transporter 14a
contig_422	Pdr-like ABC transporter
contig_2801	Mdr-like ABC transporter
Ta_S52544739	ABC transporter family protein
Ta_S58882086	Hexose transporter
contig_4842	Pdr-like ABC transporter
contig_2699	Potassium efflux antiporter
Ta_S58862613	ABC transporter b family member mitochondrial-like
Ta_S58863290	Probable udp-sugar transporter protein slc35a4-like

Ta_S52546529	ABC transporter retinal flippase subfamily
Ta_S26021526	Probable anion transporter chloroplastic-like
Ta_S58865002	Plastidic ATP adp transporter
Ta_S58864112	Peptide transporter ptr2-like
contig_3295	ABC transporter b family member 20-like
Ta_S58896581	Zip-like zinc transporter
Ta_S37750060	Mitochondrial folate transporter carrier-like
Ta_S58892345	Polyol transporter 5-like
Ta_S58897268	Amino acid transport protein
Ta_S16202616	Nitrate transporter
Ta_S16203140	Oligopeptide transporter opt family
Ta_S52544226	Zip transporter
Ta_S22368567	Probable anion transporter chloroplastic-like
Ta_S13168998	Peptide transporter ptr2-like
Ta_S22382874	Hexose transporter
contig_4383	Citrate efflux mate transporter
Ta_S17889512	Mate efflux
Ta_S32629087	Pot family expressed
contig_576	Na^+ dependent neutral amino acid transporter
Ta_S17984695	Major facilitator superfamily expressed
Ta_S17889592	Yellow stripe-like transporter 14a
Ta_S52542816	Zinc transporter zip1
Ta_S19225562	Potasium transporter
Ta_S16233185	Magnesium transporter mrs2-1
Ta_S58889179	Protein translocase protein transporter
Ta_S32585319	Potassium transporter
Ta_S58883080	Mrp-like ABC transporter
Ta_S58848588	ABC transporter d family member 1-like
Ta_S22391437	ABC transporter f family member 1-like
contig_591	Mdr-like ABC transporter
Ta_S58887521	ABC transporter c family member 8-like
Ta_S16190121	Copper transporter
contig_2550	ABC transporter b family member

Ta_S13015346	Pdr-like ABC transporter
Ta_S26020591	Tlc ATP adp transporter
Ta_S52544186	Copper ion transmembrane transporter
Ta_S58882586	Pdr-like ABC transporter
Ta_S52542652	Transporter arsb-like
Ta_S58869496	Nucleobase ascorbate transporter
Ta_S52544369	Nitrate transporter
contig_1156	Nucleotide-sugar transporter sugar porter
contig_2141	Metal-nicotianamine transporter ysl3
Ta_S22383321	Nitrate transporter
Ta_S16058118	Sucrose transporter
Ta_S52543497	Bidirectional sugar transporter sweet4-like
contig_482	Sucrose proton symporter
Ta_S52545939	Polyol transporter 5-like
Ta_S32539927	ABC transporter f family member 4-like
contig_2531	Potassium transporter
contig_5349	High-affinity potassium transporter
Ta_S58849771	Pdr-like ABC transporter
contig_1101	Sugar transporter erd6-like 5-like
Ta_S52544662	Boron transporter
Ta_S58894070	Carbohydrate transporter sugar porter transporter
Ta_S22379520	Probable inositol transporter 2-like
contig_1289	Pot family expressed
Ta_S32509085	ABC transporter family of the mitochondria family
Ta_S16058141	Polyol transporter 5-like
Ta_S16058178	Polyol transporter 5-like
Ta_S46892927	Sucrose transporter 2
Ta_S58896990	Sorbitol transporter
Ta_S52544273	Ca^{2+} antiporter cation exchanger
contig_3747	ABC transporter b family member 11-like
contig_3261	Probable potassium transporter 11-like
contig_2993	ABC transporter a family member 7-like
Ta_S16196497	Vacuolar iron transporter-like protein

contig_768	Probable metal-nicotianamine transporter ysl6-like isoform 2
Ta_S52545162	Nitrite transporter
Ta_S58903343	Bile acid Na^+ symporter family protein
Ta_S16200898	ABC transporter g family member 14-like
contig_2920	ABC transporter family protein
contig_5378	Solute carrier family facilitated glucose transporter member 8
contig_5545	Mdr-like ABC transporter
Ta_S58857435	High-affinity potassium transporter
Ta_S58898829	ABC transporter c family member 10-like isoform 1
Ta_S32668167	Protein translocase protein transporter
Ta_S58893108	GDP-mannose transporter gonst3-like
Ta_S58850908	Proline transporter
Ta_S52546402	Peptide transporter ptr2-like
Ta_S58907002	Lysine histidine transporter 1
Ta_S17888698	Magnesium transporter nipa2-like
Ta_S26025085	Vacuolar iron transporter
Ta_S52545145	Phosphate transporter 4
contig_4459	Magnesium transporter nipa2-like
contig_3982	Multidrug resistance protein ABC transporter family
Ta_S52544230	Nodulin 21 -like transporter family protein
Ta_S52546064	Probable metal-nicotianamine transporter ysl9-like
Ta_S52541759	Ammonium transporter
Ta_S18660952	Almt1
Ta_S58896944	Plastidic ATP adp transporter
Ta_S58890400	ABC transporter d family member 1
Ta_S52545074	Magnesium transporter
Ta_S37853592	Metal-nicotianamine transporter ysl3
contig_6490	ABC transporter b family member 4-like
Ta_S58904749	Peptide transporter ptr2-like
contig_4503	ABC transporter family pleiotropic drug resistance protein
Ta_S13117656	Potassium transporter
contig_6407	Mdr-like ABC transporter
Ta_S22380881	Cation efflux protein zinc

Ta_S52544260	ABC transporter g family member 7
Ta_S58859924	Plastidic glucose transporter 4-like
contig_5944	ABC transporter family expressed
Ta_S52898599	Sulfate transporter
Ta_S58867285	ABC transporter c family member 10-like isoform 2
contig_2869	ABC transporter b family member 11-like
Ta_S52546291	Potassium transporter
Ta_S58868856	ATP-binding cassette transporter
Ta_S17975072	ABC transporter g family member 14-like
Ta_S58903049	Sulfate transporter
Ta_S58883174	Sucrose transporter 2
contig_382	Oligopeptide transporter
Ta_S13015725	Peptide transporter ptr2-like
contig_1770	ABC transporter b family member 19-like
Ta_S37854042	Mate efflux family protein 5-like
Ta_S22372247	Protein ruptured pollen grain
Ta_S58856968	Yellow stripe-like transporter 12
contig_2043	ABC transporter c family member 3-like
Ta_S37910108	Potassium efflux antiporter
Ta_S12964828	Multidrug resistance protein ABC transporter family
Ta_S58859226	Sugar transporter erd6-like 4-like isoform 1
Ta_S52544989	Plastidic general dicarboxylate transporter
contig_251	Multidrug pheromone mdr ABC transporter family
Ta_S52542701	Peptide transporter ptr2-like
Ta_S32562893	Potassium transporter 25-like
contig_6744	UDP-glucuronic acid udp-n-acetylgalactosamine transporter-like
Ta_S16058200	Peptide transporter ptr3-a-like
Ta_S17888837	Probable peptide nitrate transporter at3g43790-like
Ta_S58854345	ABC transporter b family member chloroplastic-like
Ta_S52543889	Iron-phytosiderophore transporter protein yellow stripe 1
contig_1273	Pdr-like ABC transporter
Ta_S52543195	Ca^{2+} antiporter cation exchanger
contig_2645	Dma efflux transporter

Ta_S58907003	Lysine histidine transporter 1-like
Ta_S37913018	Mate efflux
Ta_S58896651	Yellow stripe-like transporter 14a
Ta_S58896102	Aluminum-activated malate transporter 9-like
Ta_S12970099	ABC transporter i family member chloroplastic-like
Ta_S58885343	Endoplasmic reticulum vesicle transporter protein
Ta_S37851284	ABC transporter b family member
Ta_S58869512	ABC transporter c family member 5-like
Ta_S37829864	Polyol transporter 5-like
Ta_S58892190	Zinc transporter at3g08650-like
contig_1968	ABC transporter b family member 20-like
Ta_S26028369	Iron -phytosiderophore uptake mediator (yellow stripe like transporter) partial
contig_3983	Equilibrative nucleoside transporter
Ta_S58888513	Proline transporter
contig_253	D-xylose-proton symporter-like 2
Ta_S41658097	Zinc transporter
Ta_S26022895	Carbohydrate transporter sugar porter transporter
contig_153	ABC transporter b family member 4-like
Ta_S58899261	Zinc transporter
contig_4465	Equilibrative nucleoside transporter 3-like
Ta_S12940628	ABC transporter b family member 11-like
Ta_S58869510	ABC transporter c family member 5-like
Ta_S58883250	ABC transporter b family member mitochondrial-like
contig_1511	ABC transporter c family protein
Ta_S58901391	Lysine histidine transporter 1-like
Ta_S22374020	Peptide transporter ptr2-like
contig_3169	Uncharacterized transporter ybr287w-like
Ta_S17974709	ABC transporter b family member 4-like
Ta_S37769891	Organic cation transporter
Ta_S52543394	Potassium transporter 25-like
Ta_S37761576	Peptide transporter ptr2
Ta_S52542399	Magnesium transporter -like family protein

Ta_S52545672	ABC transporter b family member 19-like
Ta_S18957412	Auxin efflux carrier
contig_5784	ABC transporter c family member 9-like
contig_5043	Peptide transporter ptr2-like
contig_4214	Multidrug resistance protein
Ta_S58890043	Aluminum activated citrate transporter
Ta_S52541389	Peptide transporter ptr2-b
Ta_S58868892	Adenosine 3 -phospho 5 -phosphosulfate transporter 1-like
Ta_S52544227	ABC transporter c family member 4-like
Ta_S58905498	Iron-sulfur cluster assembly protein
Ta_S58897399	Probable metal-nicotianamine transporter ysl6-like isoform 1
Ta_S58895728	ABC transporter family protein
Ta_S12976373	ABC transporter b family member 11-like
Ta_S58897923	ABC transporter f family member 1-like
Ta_S24513076	ABC transporter g family member 14-like
Ta_S52547083	Pdr-like ABC transporter
Ta_S41658095	Cadmium zinc-transporting atpase 3-like
Ta_S58852208	Udp-galactose transporter 2-like
Ta_S17889694	Organic anion transporter
Ta_S12923308	Na+ h+ antiporter
Ta_S52542448	Pdr-like ABC transporter
Ta_S12888527	Plastidic ATP adp transporter
contig_6754	ABC transporter c family member 5-like
Ta_S16058102	Sulfate transporter
Ta_S37826729	Nitrate transporter -like
contig_1656	Peptide transporter 1
Ta_S37807331	Amino acid transporter
Ta_S16058101	Zinc transporter
contig_2095	ABC glycine betaine l-proline atpase subunit
Ta_S52545804	Na^{+} dependent neutral amino acid transporter
Ta_S52542491	Choline transporter-like protein 2-like
contig_5135	Cytochrome c biogenesis c
Ta_S58897618	Auxin efflux carrier-like protein

Ta_S52543425	Potassium transporter 8-like
Ta_S26027017	Udp-galactose transporter 1-like
Ta_S13165195	Uncharacterized transporter lpg1691-like
contig_6614	Multidrug pheromone mdr ABC transporter family
contig_4326	Proline transporter
Ta_S58887505	Cation cation antiporter
Ta_S52546511	Integral membrane protein like
Ta_S26026815	High-affinity nitrate transporter -like
Ta_S52543807	Peptide transporter ptr2-like
Ta_S52542608	Sucrose transporter

[a] Fold-change in red indicates lower level of expression in colonized wheat roots (CWR); (+)ND not expressed in the N-IWR libraries; Up-regulated, Down-regulated and Expressed ESTs are shading in red, blue and yellow respectively.
