# Supplementary material for: Dual RNA-seq transcriptional analysis of wheat roots colonized by Azospirillum brasilense reveals up-regulation of nutrient acquisition and cell cycle genes
Source: BMC Genomics. 2014 May 16;15(1):378. doi: 10.1186/1471-2164-15-378 (PMC4042000; doi:10.1186/1471-2164-15-378)
Supplement: Supplementary file 12 — Additional file 12: Table S10: ESTs of Triticum aestivum encoding proteins that were grouped in transport activity by GO analysis. aFold-change in red indicates lower level of expression in colonized wheat roots (CWR); (+) ND not expressed in the N-IWR libraries; Up-regulated, Down-regulated and Expressed ESTs are shading in red, blue and yellow respectively. (PDF 752 KB) [file 12864_2013_6083_MOESM12_ESM.pdf]

Table S10 ESTs of *Triticum aestivum* encoding proteins that were grouped in transport activity by GO analysis

Sequence ID	Fold change	p-value	Sequences Description
			Up-regulated ESTs (fold change>2, p-value< 0.05)
contig_2239	4.5	0.0009	ABC transporter b family member 20-like
contig_5105	5.0	0.0024	Calcium-transporting ATPase plasma membrane-type-like
Ta_S58899343	3.1	0.0037	Metal transporter nramp6-like
contig_5883	3.6	0.0073	Amino acid permease family expressed
Ta_S17976596	3.1	0.0089	Sulphate transporter
Ta_S13177057	4.2	0.0091	Nitrate transmembrane transporter naxt1-like
Ta_S58842370	4.0	0.0155	GDP-mannose transporter
contig_6340	2.1	0.0166	Hypothetical Protein
Ta_S32501583	3.4	0.0168	Hypothetical Protein
Ta_S17897607	2.9	0.0182	Major facilitator protein
contig_2635	2.4	0.0202	ABC transporter c family member 9-like
Ta_S58894107	3.2	0.0245	Amino acid permease
Ta_S32626973	2.6	0.0292	Peptide transporter (ptr2-like)
contig_6114	3.4	0.0315	Mac perforin domain containing protein
contig_4225	2.1	0.0360	Calcium-transporting ATPase endoplasmic reticulum-type-like
Ta_S12971663	2.0	0.0367	Carrier protein
contig_6109	2.2	0.0394	Probable importin subunit beta-4-like
Ta_S58895262	2.4	0.0412	Hypothetical Protein
Ta_S13172731	3.2	0.0469	Cytochrome c oxidase polypeptide vib
			Down-regulated ESTs (fold change>2, p-value< 0.05)
contig_6543	3.9	0.0247	ABC transporter g family member 16-like
Ta_S32529059	3.8	0.0445	P-glycoprotein 1
Ta_S58884172	3.7	0.0190	Sugar transporter family expressed
contig_5892	3.1	0.0328	TGF-beta type III extracellular region
contig_4736	2.9	0.0458	Transparent testa 12 protein
Ta_S17897290	2.6	0.0037	Nascent polypeptide associated complex alpha chain
Ta_S52546220	2.6	0.0388	Tonoplast intrinsic
Ta_S58902262	2.6	0.0068	Tonoplast intrinsic protein
contig_5852	2.3	0.0059	Importin subunit beta-1-like

Ta_S17987082	2.3	0.0207	Aquaporin tip4-1
Ta_S17890271	2.3	0.0392	H^{+} Ca^{2+} exchanger
Ta_S22381224	2.2	0.0450	Potassium transporter 25-like
contig_4802	2.2	0.0257	Ap-2 complex subunit sigma-like
contig_3386	2.2	0.0127	Heavy metal p-type ATPase
Ta_S52544369	1.8	0.0143	Nitrate transporter
		Expressed ESTs	
Ta_S58863349			ABC transporter
Ta_S13199552			Not found
Ta_S16058169			Ammonium transporter amt2
contig_5490			ABC transporter c family member 9-like
Ta_S16255717			Protein
contig_3974			Peptide transporter
Ta_S26020880			Sugar transporter family expressed
Ta_S13110775			Amino acid transporter a1
contig_5564			Potassium channel skor-like
contig_2606			Phospholipid-transporting ATPase 4-like
Ta_S22371202			Cation efflux family protein
contig_4963			Cyclic nucleotide-gated ion channel 1-like
contig_4634			Clathrin assembly protein ap19 homolog
contig_3070			Importin subunit beta-1-like
contig_4259			Exportin-1-like isoform 2
Ta_S32660745			Sec14 cytosolic
contig_487			Phospholipid-translocating ATPase
contig_5460			Armadillo beta-catenin-like repeat-containing protein
contig_4243			ABC transporter d family member
contig_5051			Calcium-transporting ATPase plasma membrane-type-like
Ta_S17979060			Metal transporter nramp5-like
contig_7128			Peptide transporter expressed
Ta_S58865243			Protein transport protein sec61 subunit alpha
contig_4020			Coatomer alpha
Ta_S22385099			Mannitol dehydrogenase
contig_5935			Ap3-complex subunit beta-a-like

Ta_S58842035	At1g30450-like partial
Ta_S13047749	Metal tolerance protein c1
Ta_S12920463	PREDICTED: uncharacterized protein LOC100822706 [Brachypodium distachyon]
contig_6625	Replication factor c subunit 3-like
Ta_S58889910	Serine threonine protein kinase
contig_3978	Peptide transporter ptr2-like
Ta_S17987948	Novel plant snare expressed
Ta_S32518913	Ap-3 complex subunit mu-1-like
contig_293	Hypothetical protein osj_04420 [Oryza sativa Japonica Group]
contig_4602	Vacuolar proton translocating ATPase 100 kda subunit-like isoform 1
Ta_S17985880	Peptide transporter ptr2-like
Ta_S12891430	Pentatricopeptide repeat-containing protein mitochondrial-like
contig_2461	Calcium-transporting ATPase plasma membrane-type-like
contig_283	Exportin 1a
contig_6592	ABC transporter b family member 20-like
Ta_S52543465	Udp-galactose transporter 3
contig_3879	Phospholipid-translocating ATPase
Ta_S22377863	Cytochrome c oxidase polypeptide vb
contig_3442	Atvps11 protein
contig_1940	Vacuolar ATPase b subunit
Ta_S58867287	Multidrug resistance protein ABC transporter family
contig_3999	ABC transporter c family member 10-like isoform 2
contig_3739	Tim17 domain-containing protein
contig_2758	Vacuolar ATPase subunit h protein
Ta_S58840467	ABC transporter f family member 4-like
contig_809	Na+ h+ antiporter
contig_1558	Golgin candidate 6-like
Ta_S52541753	Hippocampus abundant transcript 1
contig_2773	Pdr-type ABC transporter-like
Ta_S52543393	Udp-galactose transporter 6
contig_1559	Golgin candidate 6-like
contig_5394	Cation-transporting ATPase

contig_5315	Pinus taeda anonymous locus 0_4543_02 genomic sequence
contig_2316	Multidrug resistance p-glycoprotein
Ta_S58896279	Mate efflux
contig_5555	Exportin 1a
contig_3981	Exportin1 protein
contig_3901	Cyclic nucleotide-gated calmodulin-binding ion channel
contig_3156	Mate efflux family protein alf5
Ta_S58897641	Plastidic glucose transporter 4
contig_3428	Solute carrier family facilitated glucose transporter member 8
Ta_S52546551	Ubiquinone biosynthesis protein ubib
Ta_S58856739	Phospholipid-transporting ATPase 1-like
Ta_S13144969	Clathrin coat assembly protein
contig_2684	Ca^{2+} transporting ATPase
Ta_S52547070	Aquaporin sip2-1
Ta_S18943257	Ammonium transporter
Ta_S52542319	ATP -ADP translocator
contig_2754	Xanthine uracil vitamin c partial
Ta_S26024704	Ubiquinol-cytochrome c reductase complex kda protein
Ta_S13022773	Cytochrome c oxidase subunit vb
Ta_S58889324	Na^{+} H^{+} antiporter
Ta_S13001517	Phospholipid-transporting ATPase 1-like
Ta_S37946965	Proton-dependent oligopeptide or low-affinity nitrate partial
contig_1993	Importin subunit beta-1-like
Ta_S17981933	At1est8-like partial
Ta_S16238874	Metal tolerance protein
contig_4846	Plastidic glucose transporter 4
contig_1260	At3g28860-like partial
Ta_S58891441	ATPase subunit 8
Ta_S37854107	Potassium transporter
Ta_S58895683	Nitrate transporter -like
contig_5863	Cytochrome c oxidase polypeptide vc
Ta_S52543388	Transmembrane expressed
Ta_S13126051	Pdr-like ABC transporter

Ta_S13130526	ADP-ribosylation factor
contig_6771	Protein real-time-like isoform 1
Ta_S17895518	Phospholipid-transporting ATPase 3-like
Ta_S52545526	Sec14 cytosolic factor-like
contig_5308	Calcium-transporting ATPase plasma membrane-type
contig_1249	Solute carrier family facilitated glucose transporter member 8
Ta_S58885140	Synaptic vesicle 2-related
contig_3250	V-type proton ATPase 116 kda subunit a isoform 1-like
contig_3753	Glutamate synthase 1
Ta_S17890420	Proline transporter 1
Ta_S17892686	ATP synthase d mitochondrial
Ta_S22496174	Na+ h+ antiporter
Ta_S58904481	ADP-ribosylation factor
contig_555	Equilibrative nucleoside transporter
Ta_S13166508	Vacuolar protein sorting-associated protein 28-1
contig_3830	ABC transporter c family member 10-like isoform 2
Ta_S26022555	Tonoplast intrinsic protein
Ta_S13147100	Ubiquinol-cytochrome c reductase complex kda protein
Ta_S58886024	Probable ion channel castor-like
contig_2332	Multidrug pheromone mdr ABC transporter family
contig_5435	Protein transparent testa 12-like
Ta_S58842340	Multidrug resistance protein ABC transporter family
Ta_S52543467	Uncharacterized amino acid permease -like
Ta_S13010915	Protein
Ta_S52542790	Snare-interacting protein keule
contig_3839	Plasma membrane ATPase
Ta_S58883662	Aquaporin nip1-2
contig_5119	Peptide transporter 1
Ta_S52546890	Mac perforin domain containing protein
contig_5580	ABC transporter b family member 20-like
contig_4276	P-type ATPase
Ta_S58855059	Mitochondrial import inner membrane translocase subunit tim8
Ta_S58867686	Mitochondrial import inner membrane translocase subunit tim22

Ta_S58902937	2-on-2 hemoglobin
contig_1208	ABC transporter d family member
Ta_S32585634	Mdr-like ABC transporter
contig_4668	Coatomer alpha subunit
Ta_S58897308	ABC transporter d family member 1
Ta_S17898791	Dmi1 protein
Ta_S22385774	Ap-4 complex subunit sigma-1
Ta_S26024082	GTP-binding protein sar1a
contig_2727	Cytochrome c oxidase subunit
Ta_S16058108	Sec14-like protein 1
Ta_S13021939	Sulphate transporter
Ta_S52544693	Calcium-transporting ATPase plasma membrane-type-like
Ta_S50389128	Peptide transporter ptr2-like
Ta_S37823111	Pinus taeda anonymous locus 0_8694_01 genomic sequence
Ta_S37777140	Heat intolerant 1 protein
contig_2537	Hypothetical protein
Ta_S58899451	Cytochrome c oxidase subunit 1
contig_2816	Probable importin-7 homolog
Ta_S58869513	Multidrug resistance protein ABC transporter family
Ta_S58887009	Pyrophosphate-energized membrane proton pump 2
Ta_S17988968	Phospholipid-transporting ATPase 9-like
Ta_S52545704	Porin voltage-dependent anion-selective channel protein
Ta_S32528722	Myosin-j heavy chain-like
Ta_S58894270	NADH dehydrogenase
Ta_S17987431	Potassium transporter
Ta_S13128371	Ureide permease
Ta_S58864836	Mitochondrial carnitine acylcarnitine carrier-like protein
Ta_S58867846	Plasma membrane calcium-transporting ATPase
Ta_S22389536	Anion channel protein
contig_2444	Coatomer alpha subunit
Ta_S52542003	Peptide transporter ptr2-like
Ta_S22378357	Protein transport protein sec61 subunit alpha
Ta_S32610474	Pii protein

Ta_S58869511	ABC transporter c family member 5-like
Ta_S58902065	Protein transport protein sec24-like at3g07100-like
Ta_S58866161	K(+) efflux antiporter 6-like
contig_3756	Armadillo beta-catenin-like repeat-containing protein
Ta_S52541510	Metal tolerance protein
Ta_S13114500	Cyclic nucleotide gated channel
Ta_S13145384	Cytochrome c oxidase polypeptide vc
Ta_S16057927	ABC transporter b family member 4-like
Ta_S17988485	Aluminum activated citrate transporter
Ta_S58868804	ATPase subunit 1
contig_618	Calcium-transporting ATPase plasma membrane- expressed
Ta_S16057986	Mate efflux family protein 7-like
Ta_S50384859	Apocytochrome b
Ta_S13256283	Amino acid transporter
contig_1668	Importin subunit beta-1-like
contig_1452	Aluminum-activated citrate transporter
Ta_S52545313	Peroxisomal carrier protein
Ta_S16058470	Zinc transporter
Ta_S58869503	Cytochrome c oxidase subunit 3
contig_2876	D-xylose-proton symporter-like 2
Ta_S37802999	Mate efflux family expressed
contig_4261	ABC transporter family expressed
contig_7157	Shaker-like potassium channel
Ta_S52544037	Metal-transporting p-type ATPase
contig_4049	Phospholipid-transporting ATPase 1-like
Ta_S12922951	ATP synthase beta chain
Ta_S58860395	Cation-chloride cotransporter 1-like
Ta_S17989018	ABC transporter family protein
Ta_S58882237	Phospholipid-transporting ATPase 1-like
Ta_S26026288	Mitochondrial import inner membrane translocase subunit tim17
contig_921	Phospholipid-transporting ATPase 3-like
Ta_S26025080	ATP synthase subunit h protein
contig_6128	Adaptor-related protein complex ap- beta 2 subunit

contig_82	Autoinhibited calcium ATPase
Ta_S13000822	Probable cyclic nucleotide-gated ion channel 17-like
Ta_S58861424	Vacuolar amino acid transporter 1-like
Ta_S13058142	ABC transporter c family member 10-like isoform 2
Ta_S26028510	Phosphate phosphoenolpyruvate translocator
Ta_S58858831	ABC transporter a family member 7-like
Ta_S58843930	Armadillo beta-catenin-like repeat-containing protein
Ta_S52542739	Protein toc75
contig_1686	Pleckstrin homology domain family a
contig_4157	Exportin 1a
Ta_S58859647	Outer envelope protein
Ta_S58858982	Glutamate receptor -like
contig_4199	Vacuolar proton
Ta_S58896454	Armadillo beta-catenin-like repeat-containing protein
Ta_S26027328	Peptide transporter ptr2-like
Ta_S19225168	Cation-chloride cotransporter
Ta_S17981975	Protein
Ta_S58843363	Potassium transporter 11-like
contig_5961	Phosphatidylinositol phophatidylcholine transfer protein
contig_4983	Vacuolar protein sorting-associated protein 11 homolog
Ta_S17986258	Transparent testa 12 protein
Ta_S32507418	Ammonium transporter
Ta_S58891463	Cytochrome c biogenesis fn
Ta_S30065904	Transportin-3-like isoform 1
Ta_S52543897	Plasma membrane h+ ATPase
Ta_S52543572	Sec1 family transport protein sly1
contig_115	Calcium-transporting ATPase plasma membrane- expressed
contig_5221	Major facilitator superfamily expressed
Ta_S26026246	Transmembrane emp24 domain-containing protein
Ta_S52543811	Chloride channel protein clc-f-like
Ta_S13141198	ABC transporter c family member 3-like
Ta_S22370202	Ubiquinol-cytochrome c reductase complex ubiquinone-binding protein qp-c

contig_5109	ABC transporter a family member 4
contig_5629	ABC transporter d family member
Ta_S52541148	Puromycin-sensitive aminopeptidase-like
Ta_S12863029	V-type proton ATPase catalytic subunit a
Ta_S17984922	Protein transport protein sec61 beta subunit
Ta_S37893286	Delta tonoplast intrinsic protein tip2 2
Ta_S58905410	V-type proton ATPase subunit d2
Ta_S52543264	Nucleolar GTP-binding protein 1-like
contig_4051	ABC transporter c family member 10-like isoform 2
Ta_S58867588	Armadillo beta-catenin-like repeat-containing protein
Ta_S24623161	Multicatalytic endopeptidase complex alpha subunit-like
contig_4341	Peptide transporter ptr2-like
Ta_S37865890	Vacuolar protein sorting-associated protein 55-like protein
contig_4961	Phospholipid-transporting ATPase 1-like
Ta_S16237305	Ap-2 complex subunit alpha-1
Ta_S12923277	Triose phosphate phosphate translocator
Ta_S52546317	Acyl-acp thioesterase
Ta_S26021646	Coatomer alpha subunit
Ta_S17889510	Transparent testa 12 protein
Ta_S58851172	Potassium transporter
Ta_S52541132	Low quality protein: uncharacterized amino-acid permease -like
Ta_S16058073	ABC transporter c family member 3-like
Ta_S58904255	ATPase subunit 4
Ta_S58865239	Protein transport protein sec61 subunit alpha-like
contig_6125	Nod26-like membrane integral protein
Ta_S58888287	Phospholipid-transporting ATPase 9-like
Ta_S58852047	ABC transporter b family member
Ta_S52544358	ABC transporter like protein
Ta_S52544007	Zip-like zinc transporter
contig_5727	Mdr-like ABC transporter
contig_3955	Coatomer alpha
Ta_S52542099	Mate efflux family protein alf5-like
Ta_S58904463	Protein transport protein sec61 subunit alpha

contig_4428	Vacuolar proton translocating ATPase 100 kda subunit-like isoform 1
Ta_S17888422	Adp-ribosylation factor 1
contig_3563	Vacuolar ATPase b subunit
Ta_S22381642	Ap-3 complex subunit sigma
Ta_S58839445	Dna replication licensing factor mcm5-a-like
Ta_S52547015	Amino acid permease 1
Ta_S22383057	Peptide transporter ptr2-like
Ta_S12891362	Carrier protein
Ta_S52545515	Nuclear pore complex protein nup98-nup96-like isoform 1
Ta_S58899507	Spinster-like protein
Ta_S16058124	Ammonium transporter
contig_2791	Aminophospholipid ATPase
contig_3544	At5g19640-like partial
contig_1309	Adenine nucleotide translocator
Ta_S22370841	Mn-specific cation diffusion facilitator transporter
Ta_S58843245	Auxin efflux carrier family protein
Ta_S58886750	Sucrose transport protein suc3
Ta_S58897736	Mate efflux family protein 5-like
contig_2996	Pleiotropic drug resistance protein
Ta_S58860384	Cation-chloride cotransporter 1-like
Ta_S52542689	Peptide transporter
contig_899	Heavy metal ATPase
Ta_S58899231	Phospholipid-transporting ATPase 3-like
Ta_S52545925	Uncharacterized amino acid permease -like
Ta_S58907622	Cytochrome c oxidase subunit 2
Ta_S52544086	Protein
Ta_S52545860	Solute carrier family facilitated glucose transporter member 8
Ta_S12870538	Plasma membrane intrinsic protein
Ta_S58902933	Ureide permease 2
Ta_S58862919	ABC transporter c family member 10-like isoform 1
Ta_S58893800	Potassium transporter 7-like
Ta_S52543797	Multidrug pheromone mdr ABC transporter family
contig_4125	Chromosome region maintenance protein 1

Ta_S58868699	Magnesium transporter mrs2-4-like
contig_3331	Vacuolar ATPase subunit h protein
Ta_S58851485	Mrp-like ABC transporter
Ta_S52544923	Transport protein
Ta_S58906320	Plasma membrane h+-ATPase
Ta_S20306454	Peptide transporter ptr2-like
Ta_S58904592	Rnase l inhibitor-like protein
Ta_S16258294	ABC transporter d family member
Ta_S26024056	Delta tonoplast intrinsic protein tip2 2
Ta_S18009268	Adenine phosphoribosyltransferase 1
Ta_S13143579	Nuclear transport factor 2
contig_2218	Vacuolar ATP synthase subunit f
Ta_S58853618	Natural resistance-associated macrophage protein
contig_3857	D-xylose-proton symporter-like 2
Ta_S26028058	Vacuolar ATP synthase 16 kda proteolipid subunit
Ta_S17985842	Auxin efflux carrier
contig_401	ABC transporter c family member 10-like
Ta_S17987421	Oligomycin sensitivity conferring protein
contig_4282	Transportin 1
Ta_S22391325	Zinc transporter ztp29-like
Ta_S52542585	Hypothetical protein
Ta_S58881750	Sulfate transporter
Ta_S52542806	Integral membrane transporter family protein
Ta_S52546225	Nucleobase-ascorbate transporter 6-like
Ta_S58902258	Tonoplast intrinsic protein
contig_4601	Vacuolar proton translocating ATPase 100 kda subunit-like isoform 1
contig_2217	Importin beta
contig_4470	Autoinhibited calcium ATPase
Ta_S52543198	Zinc transporter
Ta_S58901635	Plasma membrane intrinsic protein
Ta_S52543438	Protein transport protein sec23
Ta_S13110544	Vacuolar ATPase b subunit
Ta_S58884802	Pot family expressed

contig_4887	ABC transporter g family member 28-like
Ta_S22384411	Vacuolar ATPase b subunit
Ta_S18012476	Protein transport protein sec61 gamma subunit
Ta_S17989967	Mitochondrial import inner membrane translocase subunit tim22
contig_2375	Glutamate synthase 1
Ta_S52543355	Copper-transporting ATPase ran1-like isoform 1
Ta_S58882139	ABC transporter b family member 20-like
Ta_S58852998	Heat intolerant 1 protein
Ta_S32607163	Sec14p-like phosphatidylinositol transfer family protein
Ta_S52545986	Cation-transporting ATPase
Ta_S58890717	Peptide transporter ptr2-b
Ta_S26027792	Adp-ribosylation factor
contig_4808	ABC transporter c family member 8-like
Ta_S58885043	Glutamate receptor -like
Ta_S52545806	Mitochondrial import receptor subunit tom40
contig_6122	Acyl- oxidase acx3
Ta_S52546995	Beta-adaptin-like protein c
Ta_S58861678	Biopterin transport-related protein bt1
Ta_S58862614	ABC transporter b family member mitochondrial-like
Ta_S17865479	ABC transporter c family member 3-like
contig_833	ABC transporter b family member 19-like
Ta_S52545122	Peroxisomal acyl- oxidase 1a
Ta_S52543740	ABC transporter family protein
Ta_S52544563	Protein brittle- chloroplastic amyloplastic-like
Ta_S52545421	Mscs-like 2 protein
Ta_S32511872	Pattern formation protein emb30-like
contig_630	Tonoplast intrinsic protein
contig_5379	Potasium transporter
Ta_S52544118	Synaptic vesicle 2-related protein
Ta_S58881991	Snare-interacting protein keule
Ta_S58858964	Mitochondrial substrate carrier family protein s-like
Ta_S12923503	ABC transporter b family member 11-like
Ta_S52542361	Protein

Ta_S52544008	Ap-1 complex subunit gamma-1
Ta_S58899651	Hexose transporter
Ta_S13173768	Mate efflux family protein alf5-like
Ta_S58865173	Acyl- oxidase
Ta_S17987294	Ubiquinol-cytochrome c reductase iron-sulfur subunit
Ta_S52542683	Structural maintenance of chromosomes protein 4-like
contig_5792	Retrotransposon ty1-copia subclass
Ta_S18943256	Ammonium transporter
Ta_S16058064	Protein transparent testa 12-like
Ta_S58904947	Major facilitator superfamily expressed
contig_4204	Cytosolic factor-like protein
Ta_S58869561	ATP synthase beta chain
Ta_S58903178	Vacuolar proton-inorganic pyrophosphatase
Ta_S58888344	Calcium-transporting ATPase endoplasmic reticulum-type-like
Ta_S58863243	ABC transporter i family member chloroplastic-like
Ta_S58851719	Nuclear pore complex protein nup98-nup96
Ta_S52545549	Calcium-transporting ATPase plasma membrane-type-like
contig_4534	Nucleolar GTP-binding protein 1-like
Ta_S52545751	Phosphatidylinositol transfer-like protein ii
Ta_S58851171	Anion-transporting ATPase
Ta_S52546492	Target of myb protein 1
Ta_S12923309	Na^+ H^+ antiporter
Ta_S52541792	Copper-transporting ATPase paa1
Ta_S13205053	Vacuolar ATPase b subunit
Ta_S58898758	ABC transporter family protein
Ta_S58856407	Potassium transporter 7-like
Ta_S25791176	Na^+ H^+ antiporter
contig_1019	Acyl-coenzyme a oxidase peroxisomal-like
Ta_S17987027	Vacuolar protein sorting-associated protein 55 homolog
Ta_S52546547	Iaa-alanine resistance protein 1-like
Ta_S13043365	ATP-binding cassette
Ta_S50371846	Pdr-type ABC transporter
Ta_S58906099	ABC transporter c family member 8-like

Ta_S12923237	Plasma membrane intrinsic protein
Ta_S30404763	Vacuolar ATP synthase subunit
Ta_S52543415	Metal transporter nramp3-like
Ta_S26025374	Ubiquinol-cytochrome c reductase complex ubiquinone-binding protein qp-c
Ta_S58863315	Bile acid transporter 5
contig_3077	Cytochrome c biogenesis protein
Ta_S37760734	Mate efflux family protein
contig_3540	Peptide transporter ptr2-like
Ta_S52544430	Synaptic vesicle 2-related
Ta_S17988029	Multidrug resistance protein
Ta_S58840767	GTP-binding protein
contig_2049	Heavy metal p-type ATPase
Ta_S52545109	Beta-adaptin-like protein a
contig_5000	Dmi1 protein
Ta_S52547002	Translocation protein sec62
Ta_S16218736	Adenosine 3 -phospho 5 -phosphosulfate transporter 1-like
Ta_S17989788	ATP synthase delta
contig_5035	Cytochrome c biogenesis c
Ta_S52546797	Probable peptide nitrate transporter at1g59740-like
Ta_S17989748	Calcium-binding mitochondrial carrier protein s -1-like
contig_3765	Sucrose transporter
Ta_S52544666	Upf0051 protein chloroplastic-like
Ta_S58844672	Metal transporter nramp6-like
Ta_S52545945	ATP synthase subunit
contig_3703	Equilibrative nucleoside transporter 4-like
Ta_S17977242	ABC transporter i family member 17
Ta_S58896318	Potassium transporter
Ta_S58697407	High-affinity potassium transporter
Ta_S17880402	Integral membrane protein
Ta_S58883545	Structural maintenance of chromosomes 1
Ta_S58901302	Coiled-coil domain-containing protein 130-like
Ta_S58891137	Sec14p-like phosphatidylinositol transfer family protein

contig_4657	Plastidic glucose transporter 4-like
Ta_S58858093	Protein
contig_4862	Heavy metal ATPase
Ta_S17898000	Sugar transporter
contig_3468	Equilibrative nucleoside transporter
Ta_S58868762	Chloride channel protein
Ta_S52543186	Peptide transporter ptr2-like
Ta_S58904258	ATPase subunit 6
Ta_S52546866	Inositol transporter 1
contig_1462	Pleckstrin homology domain family a
Ta_S58856205	Protein transport protein sec24-like at4g32640-like
Ta_S52546249	Cationic amino acid transporter
Ta_S17977147	Polyol transporter 5-like
contig_2329	Vacuolar ATPase b subunit
Ta_S52546724	Plastidic glucose transporter 4
contig_5176	ATP synthase subunit mitochondrial-like
Ta_S58893716	Sucrose transporter
Ta_S32545039	Ap3-complex subunit beta-a-like
Ta_S52544955	Amino acid carrier
Ta_S16833772	Two-pore calcium channel
Ta_S58851875	Glutamate receptor -like
Ta_S16057847	Vacuolar protein sorting-associated protein 45 homolog
Ta_S58902373	ABC transporter c family member 2-like
Ta_S52545304	Chloride channel
contig_2710	ABC transporter c family member 10-like isoform 2
Ta_S58857789	Transportin 1
contig_2990	Ap-2 complex subunit alpha-1
Ta_S52543655	Nucleobase-ascorbate transporter 6-like
Ta_S52543960	Phosphatidylinositol transfer
contig_1302	V-type proton ATPase 116 kda subunit a isoform 1-like
Ta_S17890892	Ubiquinol-cytochrome c reductase complex 14 kda protein
Ta_S58897837	Protein transport protein sec61 beta subunit
Ta_S26027263	Transmembrane expressed

Ta_S52546483	K(+) efflux antiporter chloroplastic-like
Ta_S58891022	ABC transporter a family member 7-like
contig_6673	Cation h+ exchanger
Ta_S17987255	Delta tonoplast intrinsic protein tip2 2
Ta_S58860645	Mitochondrial substrate carrier family protein
contig_6107	Glutamate synthase 1
Ta_S58897309	ABC transporter d family member 1
contig_3811	Endoplasmic reticulum-type calcium-transporting ATPase 4
Ta_S58885850	Oligopeptide transporter
Ta_S17888779	Chloride channel
Ta_S17888502	Protein
Ta_S52542520	Sodium hydrogen exchanger 6-like
Ta_S58909002	ABC transporter c family member 10-like isoform 1
contig_1481	Solute carrier family facilitated glucose transporter member 8
Ta_S58897107	Chloride channel
Ta_S37805390	Monosaccharide transporter 1
Ta_S52542911	Organic anion transporter
Ta_S52546602	Sugar transport protein 5
contig_398	ABC transporter b family member 19-like
Ta_S58869162	ATPase subunit 6
Ta_S26025803	Adp-ribosylation factor
contig_798	Probable importin-7 homolog
Ta_S12923233	Potassium channel akt1-like
Ta_S12899217	Protein
Ta_S22366111	Temperature-induced lipocalin
contig_4118	Gamma-adaptin 1
contig_2489	Plasma membrane h+-ATPase
contig_1116	Acyl- oxidase acx3
Ta_S16227814	Auxin efflux carrier
Ta_S52542190	Anion-transporting ATPase family protein
Ta_S58904990	Calcium-transporting ATPase plasma membrane-type-like
Ta_S17984911	Mate efflux family protein 5-like
Ta_S16058136	Mitochondrial uncoupling protein 3

Ta_S22390741	Leucine zipper protein zip1
Ta_S58902653	V-type h+-transporting ATPase subunit i
Ta_S58882281	Ap-2 complex subunit alpha-1
contig_7143	Atvps11 protein
Ta_S58732267	Cadmium zinc-transporting ATPase 3-like
contig_4286	Shaker-like potassium channel
Ta_S22388588	Zinc transporter ztp29-like
Ta_S52541089	Cytochrome c oxidase assembly protein cox15-like
contig_4848	Cytochrome c biogenesis b
Ta_S26022608	Mitochondrial import inner membrane translocase subunit tim17 tim22 tim23 family protein
contig_2801	Mdr-like ABC transporter
Ta_S52544739	ABC transporter family protein
Ta_S17894256	Vacuolar ATP synthase subunit g
contig_162	P-type ATPase
contig_1259	Triose phosphate phosphate non-green precursor
contig_5776	Calcium-transporting ATPase endoplasmic reticulum-type-like
contig_430	Golgin candidate 6-like
Ta_S58882086	Hexose transporter
Ta_S52546010	Aminophospholipid ATPase
Ta_S58903294	Ap-1 complex subunit gamma-2-like
contig_766	Protein transparent testa 12-like
Ta_S18011873	ATP synthase epsilon mitochondrial
Ta_S58869914	Glucose-6-phosphate translocator
contig_2699	Potassium efflux antiporter
Ta_S58862613	ABC transporter b family member mitochondrial-like
Ta_S58839689	Cellular apoptosis susceptibility protein
Ta_S58896020	Voltage-dependent anion channel
contig_5229	Tumor suppressor
Ta_S58863290	Probable udp-sugar transporter protein slc35a4-like
Ta_S52546529	ABC transporter retinal flippase subfamily
Ta_S26021526	Probable anion transporter chloroplastic-like
Ta_S58865002	Plastidic ATP adp transporter

Ta_S13170574	Retrotransposon ty1-copia subclass
Ta_S12984597	Calcium-binding mitochondrial carrier protein s -1-like
Ta_S52545757	Sc3 protein
Ta_S58864112	Peptide transporter ptr2-like
contig_4609	Phospholipid-transporting ATPase 9-like
Ta_S26020190	Hydrogen-transporting ATP rotational mechanism
Ta_S52544750	Plastidic phosphate translocator-like protein1
contig_3295	ABC transporter b family member 20-like
contig_60	Plasma membrane h+-ATPase
Ta_S43287760	V-type proton ATPase subunit c-like
Ta_S52543089	Vacuolar-sorting receptor 7-like
contig_2961	Aquaporin pip
Ta_S52543870	Potassium channel beta subunit
contig_1934	Probable cyclic nucleotide-gated ion channel 17-like
Ta_S58896581	Zip-like zinc transporter
Ta_S22373784	Bet1 sft1-related snare
Ta_S52544660	Xanthine uracil permease family expressed
Ta_S17878602	Mac perforin domain-containing protein
Ta_S37750060	Mitochondrial folate transporter carrier-like
contig_4044	Glutamate synthase 1
Ta_S58841527	Protein
contig_2128	Chloride channel
Ta_S52543845	Monosaccharide-sensing protein 2-like
Ta_S26022634	Acyl carrier protein 3
Ta_S58882045	Peptide transport protein
Ta_S26027002	Vacuolar ATP synthase 16 kda proteolipid subunit
Ta_S58839498	Ran-binding protein
Ta_S22383279	Copper-transporting ATPase ran1-like isoform 1
Ta_S58883241	Mitochondrial rieske iron-sulfur protein
Ta_S58892345	Polyol transporter 5-like
Ta_S58902233	Calcium-transporting ATPase endoplasmic reticulum-type-like
Ta_S17989856	Protein transport protein sec61 gamma subunit
Ta_S16202616	Nitrate transporter

Ta_S16203140	Oligopeptide transporter opt family
Ta_S52544226	Zip transporter
Ta_S13168998	Peptide transporter ptr2-like
Ta_S22382874	Hexose transporter
Ta_S52541889	Aquaporin sip1-2
Ta_S58856386	Calcium-binding mitochondrial carrier protein s -1-like
contig_4383	Citrate efflux mate transporter
Ta_S17889512	Mate efflux
Ta_S58848084	ATP synthase cf1 alpha subunit
Ta_S13044461	Proton-dependent oligopeptide transport family protein
Ta_S58850911	Copper-exporting ATPase
Ta_S32629087	Pot family expressed
Ta_S17984695	Major facilitator superfamily expressed
Ta_S26022980	Probable aquaporin pip2-7-like
Ta_S58868686	Mitochondrial import receptor subunit tom40
Ta_S58889000	Protein
Ta_S22380521	Mitochondrial import inner membrane translocase subunit tim13
Ta_S13265990	Probable cadmium zinc-transporting ATPase chloroplastic-like
Ta_S58854293	Metal tolerance protein
Ta_S52542816	Zinc transporter zip1
Ta_S58860533	Coiled-coil domain-containing protein 130-like
Ta_S19225562	Potasium transporter
Ta_S16233185	Magnesium transporter mrs2-1
Ta_S58889179	Protein translocase protein transporter
Ta_S50379788	Adenine guanine permease azg1-like
Ta_S32585319	Potassium transporter
contig_1626	Sec14 cytosolic factor-like
contig_2673	Peroxin pex14
contig_6271	Multidrug resistance p-glycoprotein
contig_335	Probable importin subunit beta-4-like
contig_2433	Phospholipid-translocating ATPase
contig_285	Mitochondrial inner membrane translocating protein
Ta_S52544458	Coiled-coil domain-containing protein 130-like

Ta_S58852687	Permease 1
Ta_S22391437	ABC transporter f family member 1-like
Ta_S58866999	Preprotein translocase subunit secy-like
contig_591	Mdr-like ABC transporter
Ta_S18011530	Protein
Ta_S58900128	Transparent testa 12 protein
Ta_S58887521	ABC transporter c family member 8-like
Ta_S16190121	Copper transporter
Ta_S58864584	Callose synthase 5-like
Ta_S58856694	Copper-transporting ATPase ran1-like
contig_2550	ABC transporter b family member
Ta_S37808596	Mitochondrial substrate carrier family protein
Ta_S58838859	Aquaporin sip1-2
Ta_S52542158	Chloride channel protein clc-d
Ta_S52544186	Copper ion transmembrane transporter
Ta_S12951319	Mitochondrial import receptor subunit tom7-1
Ta_S52542652	Transporter arsb-like
Ta_S22367110	Pattern formation protein emb30
Ta_S26020095	V-type proton ATPase subunit brain isoform
Ta_S58869496	Nucleobase ascorbate transporter
contig_1156	Nucleotide-sugar transporter sugar porter
Ta_S58905116	Cytochrome b6-f complex iron-sulfur subunit
contig_2439	Coatomer alpha subunit-like protein
contig_6520	Structural maintenance of chromosomes protein 4-like
contig_6113	Protein hasty 1-like
contig_4707	Protein
contig_2141	Metal-nicotianamine transporter ysl3
contig_2658	Heavy metal p-type ATPase
Ta_S22383321	Nitrate transporter
Ta_S16058118	Sucrose transporter
Ta_S58847746	Vacuolar protein sorting-associated protein 33-like protein
Ta_S58889854	Aquaporin sip2-1
Ta_S32665593	Solute carrier family 40 member 1-like

Ta_S18011994	ATP synthase epsilon mitochondrial
Ta_S22381887	Protein
Ta_S52545939	Polyol transporter 5-like
Ta_S32539927	ABC transporter f family member 4-like
Ta_S58854433	Ccr4-not transcription complex subunit 1-like
contig_2531	Potassium transporter
Ta_S18007048	Nuclear transport factor 2
Ta_S18012189	Tom7-like protein
Ta_S52542561	Protein
Ta_S37751594	Pinus taeda anonymous locus 2_2352_02 genomic sequence
Ta_S32666729	Inward rectifying shaker k+ channel
contig_705	Nucleolar GTP-binding protein 1-like
Ta_S58851961	Amino acid permease 6
Ta_S58891533	Adp-ribosylation factor
contig_1430	Mitochondrial import inner membrane translocase subunit tim44
contig_5349	High-affinity potassium transporter
Ta_S58863258	Protein hasty 1-like
Ta_S52896028	Protein transport protein sec61 beta subunit
Ta_S58849771	Pdr-like ABC transporter
Ta_S12933474	Plasma membrane intrinsic protein
Ta_S17888215	Adenine guanine permease azg2-like
contig_5278	Structural maintenance of chromosomes protein 4
contig_1101	Sugar transporter erd6-like 5-like
Ta_S17987672	Sec-independent protein translocase
Ta_S52544662	Boron transporter
Ta_S22379520	Probable inositol transporter 2-like
contig_1289	Pot family expressed
Ta_S13114578	Cation-transporting ATPase
Ta_S32509085	ABC transporter family of the mitochondria family
contig_2726	Importin subunit beta-1-like
contig_4667	Protein
contig_1237	Transportin 1
Ta_S16058141	Polyol transporter 5-like

contig_2553	Acyl- oxidase acx3
Ta_S16058178	Polyol transporter 5-like
Ta_S12864316	Plasma membrane h+ ATPase
Ta_S46892927	Sucrose transporter 2
Ta_S58903293	Ap-1 complex subunit gamma-1
Ta_S52543990	Flagellar biosynthesis protein expressed
Ta_S58896990	Sorbitol transporter
Ta_S58863330	Thiamin pyrophosphokinase 1
Ta_S52544273	Ca2+ antiporter cation exchanger
contig_4514	Vacuolar protein sorting-associated protein 11 homolog
contig_3747	ABC transporter b family member 11-like
contig_3261	Probable potassium transporter 11-like
Ta_S58861391	Dna replication licensing factor mcm5-a-like
Ta_S16058468	Aux1-like permease
Ta_S52544633	P-type ATPase
Ta_S52546908	Sugar transport protein 14
contig_3672	Protein hasty 1-like
Ta_S13109564	Amino acid permease family expressed
Ta_S52545921	Preprotein translocase subunit chloroplastic-like
contig_6236	Cation-transporting ATPase
Ta_S13145956	Nod26-like major intrinsic protein
Ta_S52545162	Nitrite transporter
Ta_S58903343	Bile acid na+ symporter family protein
Ta_S16200898	ABC transporter g family member 14-like
Ta_S58883086	Mitochondrial import inner membrane translocase subunit tim9
Ta_S17987465	Nascent polypeptide-associated complex alpha subunit-like protein
contig_3286	Calcium-transporting ATPase plasma membrane-type-like
Ta_S18012117	Protein transport protein sec61 gamma subunit
contig_5378	Solute carrier family facilitated glucose transporter member 8
contig_233	Phospholipid-transporting ATPase 9-like
contig_1003	Pleiotropic drug resistance protein 4-like
Ta_S13172107	Cellular retinaldehyde-binding triple function
Ta_S58859664	Heat repeat family expressed

contig_2379	Ap-2 complex subunit alpha-1
Ta_S52544554	Glutamate receptor -like
Ta_S52895793	ATP synthase cf0 subunit iv
Ta_S52542624	Dna repair protein reca homolog mitochondrial-like
Ta_S32625916	Transmembrane expressed
contig_4255	Copper-transporting ATPase 3-like
contig_5545	Mdr-like ABC transporter
Ta_S58882933	Protein
Ta_S58857435	High-affinity potassium transporter
contig_3093	Acyl carrier protein
Ta_S58898829	ABC transporter c family member 10-like isoform 1
Ta_S52546459	Sec14 cytosolic factor family protein
Ta_S32668167	Protein translocase protein transporter
Ta_S52543585	Grave disease carrier
Ta_S58850908	Proline transporter
Ta_S18008684	Cytochrome c oxidase polypeptide vib
Ta_S12942846	Autoinhibited ca2+-ATPase 1
Ta_S58857313	Adp-ribosylation factor
Ta_S58896787	Delta tonoplast intrinsic protein tip2 2
Ta_S58869086	V-type h+-transporting ATPase subunit i
Ta_S52546402	Peptide transporter ptr2-like
contig_2434	Golgin candidate 6-like
Ta_S58907002	Lysine histidine transporter 1
contig_5893	Heat intolerant 1 protein
Ta_S32634861	Phospholipid-transporting ATPase 3-like
contig_3566	Phospholipid-transporting ATPase 9-like
Ta_S52544014	Glucose-6-phosphate translocator
Ta_S26025085	Vacuolar iron transporter
Ta_S13147310	Ubiquinol-cytochrome c reductase complex 14 kda protein
Ta_S52545145	Phosphate transporter 4
Ta_S24513010	Chloride channel protein clc-a
contig_3982	Multidrug resistance protein ABC transporter family
contig_7080	GTP-binding protein sar1a

Ta_S13025897	Permease 1
contig_3919	ATP synthase cf1 epsilon subunit
Ta_S58868698	Transmembrane emp24 domain-containing protein
Ta_S52541759	Ammonium transporter
contig_3616	Vacuolar proton
Ta_S13056037	Protein
Ta_S18660952	Almt1
Ta_S22381712	Protein
Ta_S58896944	Plastidic ATP adp transporter
contig_6789	Importin beta-like protein
contig_4952	Autoinhibited ca2+-ATPase 1
Ta_S58869566	ATP synthase beta subunit
Ta_S58842080	Probable envelope carrier chloroplastic-like
Ta_S58896554	Syntaxin 72
Ta_S58890400	ABC transporter d family member 1
contig_6277	Vacuolar-type h+-ATPase subunit b3 (vha-b3)
Ta_S37806351	Ap-1 complex subunit gamma-2
Ta_S32507255	Btb poz domain-containing protein at5g41330-like
Ta_S58849599	Protein paused
Ta_S12965097	P-glycoprotein 1
Ta_S52545074	Magnesium transporter
Ta_S37853592	Metal-nicotianamine transporter ysl3
contig_6490	ABC transporter b family member 4-like
Ta_S58904749	Peptide transporter ptr2-like
contig_4503	ABC transporter family pleiotropic drug resistance protein
Ta_S17985860	Mitochondrial import inner membrane translocase subunit tim8
Ta_S13117656	Potassium transporter
contig_6407	Mdr-like ABC transporter
contig_3166	Nitrate
Ta_S22380881	Cation efflux protein zinc
Ta_S52544260	ABC transporter g family member 7
Ta_S58859924	Plastidic glucose transporter 4-like
Ta_S16058325	Anion-transporting ATPase

Ta_S58862472	Sam domain family protein
Ta_S58902260	Tonoplast intrinsic protein
Ta_S39723861	Aquaporin
Ta_S58849529	Nucleotide-sensitive chloride conductance regulator family protein
Ta_S58902024	Inositol-3-phosphate synthase
Ta_S58856713	Ap-4 complex subunit sigma-1
Ta_S58906314	Plasma membrane h+-ATPase
Ta_S52544997	Random slug protein 5-like
Ta_S52898599	Sulfate transporter
Ta_S58867285	ABC transporter c family member 10-like isoform 2
contig_724	Phospholipid-transporting ATPase 9-like
Ta_S52544397	Rna splicing protein
contig_2869	ABC transporter b family member 11-like
Ta_S52546291	Potassium transporter
Ta_S58860907	P-type ATPase
Ta_S58897494	Protein translocase subunit chloroplastic-like
contig_1459	Acyl- oxidase acx3
Ta_S52544718	Nuclear division rft1-like protein
Ta_S17975072	ABC transporter g family member 14-like
Ta_S16266289	Phosphoenolpyruvate phosphate translocator chloroplastic-like
contig_2995	H+-translocating pyrophosphatase
Ta_S52545029	Protein
Ta_S41574387	Vacuolar H^+-translocating inorganic pyrophosphatase
Ta_S52544630	Importin 9
contig_3006	Protein
Ta_S58903049	Sulfate transporter
contig_382	Oligopeptide transporter
contig_1230	Calcium-transporting ATPase endoplasmic reticulum-type-like
Ta_S52542984	Amino acid permease
Ta_S12923238	Plasma membrane intrinsic protein
contig_1770	ABC transporter b family member 19-like
Ta_S37854042	Mate efflux family protein 5-like
Ta_S16195397	GTPase sar1

Ta_S13112575	Vacuolar ATPase subunit h protein
Ta_S52544949	Autoinhibited calcium ATPase
Ta_S12923032	Voltage-dependent anion channel
contig_2043	ABC transporter c family member 3-like
Ta_S17888266	Metal tolerance protein c1
contig_3728	Coatomer alpha
Ta_S58857130	Cation proton exchanger 1a
Ta_S37910108	Potassium efflux antiporter
Ta_S52546622	Potassium channel akt2 3-like
Ta_S18012568	Vacuolar ATP synthase 16 kda proteolipid subunit
Ta_S12964828	Multidrug resistance protein ABC transporter family
Ta_S26027375	GTP-binding protein sar1a
contig_4875	Protein transport protein sec23-like
Ta_S12923239	Pip1 protein
contig_99	Proton-dependent oligopeptide transport family protein
Ta_S58859226	Sugar transporter erd6-like 4-like isoform 1
Ta_S52544989	Plastidic general dicarboxylate transporter
Ta_S37754604	Protein
Ta_S52545455	Importin subunit alpha-1-like
Ta_S12933475	Pip1 protein
Ta_S58859199	Syntaxin 72
contig_3828	Copper-transporting ATPase ran1-like isoform 2
contig_251	Multidrug pheromone mdr ABC transporter family
contig_3365	Copper-transporting ATPase ran1-like isoform 2
Ta_S22377943	Protein
contig_2238	Pip1 protein
Ta_S58869427	ATP synthase cf1 alpha subunit
Ta_S52542701	Peptide transporter ptr2-like
Ta_S32562893	Potassium transporter 25-like
contig_4272	Probable importin-7 homolog
Ta_S52545624	ATP synthase subunit mitochondrial-like
contig_6836	Transparent testa 12 protein
Ta_S16058200	Peptide transporter ptr3-a-like

Ta_S58854345	ABC transporter b family member chloroplastic-like
contig_1728	Heavy metal ATPase
Ta_S17984942	Peptide transport protein
Ta_S52543709	Inner membrane protein albino3
contig_7051	Pip1 protein
Ta_S52546690	Phospholipid-translocating ATPase
Ta_S52545871	Mitochondrial uncoupling protein
Ta_S52543195	Ca^{2+} antiporter cation exchanger
Ta_S26026283	Acyl carrier protein
contig_5797	Exportin-1-like isoform 1
Ta_S13116503	Probable importin-7 homolog
Ta_S52544753	Cellular retinaldehyde-binding triple function
Ta_S37913018	Mate efflux
Ta_S16057997	Importin subunit alpha-1-like
contig_636	Ap-2 complex subunit alpha-1
Ta_S26027653	Cyclic nucleotide-gated calmodulin-binding ion channel
Ta_S26026318	Plasma membrane intrinsic protein
contig_370	Vacuolar cation proton exchanger 5
Ta_S17988965	Ubiquinol-cytochrome c reductase iron-sulfur subunit
Ta_S12970099	ABC transporter i family member chloroplastic-like
Ta_S58862485	Chloride channel
Ta_S37854430	Cytochrome c subunit vib family protein
Ta_S58901634	Plasma membrane intrinsic protein
Ta_S58887777	Ethylene-insensitive protein 2-like
Ta_S17985869	Uncoupling protein
Ta_S37851284	ABC transporter b family member
Ta_S58869512	ABC transporter c family member 5-like
contig_2482	Phospholipid-transporting ATPase 9-like
Ta_S13137501	V-type proton ATPase subunit b2
Ta_S37829864	Polyol transporter 5-like
Ta_S52541369	Like phosphatidylinositol transfer protein
Ta_S58892190	Zinc transporter at3g08650-like
contig_3844	Myosin 1

contig_1968	ABC transporter b family member 20-like
Ta_S52543321	Coatomer subunit epsilon
Ta_S13031943	Autophagy protein 5
Ta_S52543837	Importin subunit beta-1-like
Ta_S17989470	Beta-mannosidase
Ta_S58892663	ATP-binding cassette
contig_3983	Equilibrative nucleoside transporter
Ta_S58888513	Proline transporter
contig_3051	Armadillo beta-catenin-like repeat-containing protein
Ta_S58866770	Condensin complex components subunit
Ta_S52544658	Protein transparent testa 12-like
contig_253	D-xylose-proton symporter-like 2
Ta_S37894959	Glutamate-gated kainate-type ion channel receptor subunit 5
Ta_S52542563	Nuclear pore complex protein nup155
Ta_S41658097	Zinc transporter
contig_1517	Copper-transporting ATPase 3-like
Ta_S13272681	Carrier protein
Ta_S58884943	Importin subunit beta-1-like
Ta_S58899261	Zinc transporter
contig_4465	Equilibrative nucleoside transporter 3-like
Ta_S12940628	ABC transporter b family member 11-like
Ta_S17986985	Nitrate
Ta_S58869510	ABC transporter c family member 5-like
Ta_S58883250	ABC transporter b family member mitochondrial-like
contig_5031	Pleiotropic drug resistance protein
Ta_S22366915	Chloride channel
Ta_S58907103	Calcium-transporting ATPase endoplasmic reticulum-type-like
contig_1511	ABC transporter c family protein
Ta_S22374020	Peptide transporter ptr2-like
Ta_S58843367	Novel plant snare 11
Ta_S58904602	Outward-rectifying potassium channel
Ta_S52543376	Protein transparent testa 12-like
contig_3052	Sec14 cytosolic factor

Ta_S17974709	ABC transporter b family member 4-like
Ta_S26025103	Mitochondrial import inner membrane translocase subunit tim9
Ta_S37769891	Organic cation transporter
Ta_S16058248	Sec14 cytosolic factor-like
Ta_S52544511	Mitochondrial carnitine acylcarnitine carrier-like protein
Ta_S32128181	Vacuolar proton-ATPase
Ta_S58862070	Metal tolerance protein
Ta_S52543394	Potassium transporter 25-like
contig_5578	Probable importin-7 homolog
Ta_S37761576	Peptide transporter ptr2
Ta_S52542399	Magnesium transporter -like family protein
Ta_S58847882	Phospholipid-translocating p-type flippase family expressed
Ta_S52545672	ABC transporter b family member 19-like
contig_2825	Ubiquinol-cytochrome c reductase iron-sulfur subunit
Ta_S58897290	Nucleolar GTP-binding protein 1-like
contig_737	Plasma membrane h+-ATPase
contig_45	Nuclear transport factor 2
Ta_S58869810	Hippocampus abundant transcript-like protein
contig_3188	ATP synthase subunit mitochondrial-like
Ta_S58901087	GTP-binding protein
Ta_S18957412	Auxin efflux carrier
contig_5784	ABC transporter c family member 9-like
contig_5043	Peptide transporter ptr2-like
contig_4214	Multidrug resistance protein
Ta_S58891743	Protein transport protein sec24-like at4g32640-like
contig_6057	Protein
Ta_S58890043	Aluminum activated citrate transporter
Ta_S52541389	Peptide transporter ptr2-b
Ta_S58868892	Adenosine 3 -phospho 5 -phosphosulfate transporter 1-like
contig_232	Outer plastidial membrane protein porin
Ta_S52544227	ABC transporter c family member 4-like
Ta_S52542942	Protein trigalactosyldiacylglycerol chloroplastic-like
Ta_S58886286	Phospholipid-transporting ATPase 1-like

Ta_S58905498	Iron-sulfur cluster assembly protein
Ta_S58864224	Pip1 protein
Ta_S58890041	Hypothetical protein MTR_1g006180 [Medicago truncatula]
contig_5187	Vacuolar ATPase subunit h protein
Ta_S52545715	Glycerol 3-phosphate permease
contig_1491	Protein kinase and pp2c-like domain-containing protein
Ta_S58897399	Probable metal-nicotianamine transporter ysl6-like isoform 1
contig_2916	Golgin candidate 6-like
Ta_S58884782	Protein
Ta_S52542814	Glutamate receptor -like
Ta_S58895728	ABC transporter family protein
Ta_S12976373	ABC transporter b family member 11-like
Ta_S58897923	ABC transporter f family member 1-like
contig_5937	Vacuolar-type h+-ATPase subunit b3 (vha-b3)
Ta_S52546366	Translocase of chloroplast 34
contig_6655	P-type ATPase
Ta_S22378152	Hydrogen-transporting ATP rotational mechanism
contig_685	Exportin 1a
Ta_S24513076	ABC transporter g family member 14-like
Ta_S52895572	ATP synthase cf0 subunit i
Ta_S58866912	Endoplasmic reticulum
Ta_S58903613	Major facilitator superfamily expressed
Ta_S52547083	Pdr-like ABC transporter
contig_1501	Plasma membrane ca2+-ATPase
Ta_S52546283	Magnesium-protoporphyrin ix monomethyl ester
Ta_S41658095	Cadmium zinc-transporting ATPase 3-like
Ta_S52541526	Mitochondrial import inner membrane translocase subunit tim17 tim22 tim23 family protein
contig_683	Multidrug resistance
Ta_S52543631	Plasma membrane h+-ATPase
Ta_S52545320	Vacuolar ATP synthase 21 kda proteolipid subunit
contig_2459	Autoinhibited calcium ATPase
Ta_S58863788	Cral trio domain containing protein

Ta_S58866214	V-type proton ATPase subunit b2
Ta_S52543292	Amino acid permease 6
Ta_S58843622	Kda vesicle transport
Ta_S17889694	Organic anion transporter
Ta_S52542745	Pattern formation protein emb30
Ta_S12923308	Na^+ H^+ antiporter
Ta_S12888527	Plastidic ATP adp transporter
contig_6754	ABC transporter c family member 5-like
Ta_S52541537	Aux1-like permease
contig_5637	Like phosphatidylinositol transfer protein
Ta_S16058102	Sulfate transporter
contig_1908	Vacuolar protein sorting-associated protein 11 homolog
Ta_S37826729	Nitrate transporter -like
contig_1656	Peptide transporter 1
Ta_S52546340	Transparent testa 12 protein
Ta_S58865139	Phospholipid-translocating ATPase
Ta_S52546801	Ripening regulated protein
Ta_S37807331	Amino acid transporter
Ta_S22369143	Sugar carrier protein c-like
Ta_S16058101	Zinc transporter
Ta_S17978646	Coatomer subunit alpha-3-like
contig_2095	ABC glycine betaine l-proline ATPase subunit
contig_3366	Probable importin-7 homolog
Ta_S52545804	Na+ dependent neutral amino acid transporter
contig_2224	Phospholipid-transporting ATPase 9-like
contig_1480	Ap-1 complex subunit gamma-2
Ta_S58863882	Coatomer epsilon subunit
Ta_S24623194	Amino acid selective channel protein
contig_1282	Acyl carrier protein 5
contig_5135	Cytochrome c biogenesis c
Ta_S58897618	Auxin efflux carrier-like protein
contig_2132	Phospholipid-translocating ATPase
Ta_S52543238	Cyclic nucleotide gated channel

Ta_S52543425	Potassium transporter 8-like
Ta_S26027017	UDP-galactose transporter 1-like
contig_6614	Multidrug pheromone mdr ABC transporter family
Ta_S17878740	Nuclear transport factor 2
contig_4326	Proline transporter
Ta_S52543536	Kda vesicle transport
Ta_S22378216	Vacuolar H^+-pyrophosphatase
Ta_S58883600	Solute carrier family 22 member 15-like protein
Ta_S52544433	Mitochondrial carrier protein
Ta_S17987355	ADP-ribosylation factor
Ta_S22370682	Copper-transporting ATPase 3-like
Ta_S52543684	Cation calcium exchanger 4
Ta_S52542608	Sucrose transporter

[a] Fold-change in red indicates lower level of expression in colonized wheat roots (CWR); (+)ND not expressed in the N-IWR libraries; Up-regulated, Down-regulated and Expressed ESTs are shading in red, blue and yellow respectively.
