# Supplementary material for: Dual RNA-seq transcriptional analysis of wheat roots colonized by Azospirillum brasilense reveals up-regulation of nutrient acquisition and cell cycle genes
Source: BMC Genomics. 2014 May 16;15(1):378. doi: 10.1186/1471-2164-15-378 (PMC4042000; doi:10.1186/1471-2164-15-378)
Supplement: Supplementary file 13 — Additional file 13: Table S11: ESTs of Triticum aestivum encoding proteins related to cell cycle regulation. aFold-change in red indicates down-regulation in colonized wheat roots (CWR). (PDF 17 KB) [file 12864_2013_6083_MOESM13_ESM.pdf]

Table S11 ESTs of *Triticum aestivum* encoding proteins related to cell cycle regulation

Sequence ID	Fold Change[a]	*p-value*	Sequence description
Ta_S58854607	59.8	0.0012	Replication factor C
Ta_S52547097	46.7	0.0285	Cyclin-Dependent protein Kinase
Ta_S58857445	9.4	0.0093	Serine threonine-protein kinase rio1
Ta_S59490000	6.9	0.0465	MYB transcription factor
Ta_S58842184	5.7	0.0282	Serine threonine-protein kinase rio1-like
contig_3741	3.7	0.0003	Beta-expansin 2 precursor
Ta_S32510701	3.5	0.0240	Mitogen-activated protein kinase 6-like (MAPK)
contig_1285	3.3	0.0489	Microtubule organization protein
contig_3699	2.8	0.0253	Histidine kinase
contig_708	2.6	0.0417	Secondary cell wall-related glycosyltransferase (TaGT47-12)
contig_5714	2.6	0.0393	Actin depolymerizing factor 4
Ta_S17893420	2.4	0.0084	Beta-expansin
Ta_S20999571	2.3	0.0303	Xyloglucan endotransglucosylase hydrolase
contig_5981	2.3	0.0200	Serine threonine-protein kinase sapk3-like (MAP kinase 12)
contig_5777	2.2	0.0345	Beach domain-containing protein (lvsA-like)
contig_1554	2.1	0.0463	Actin-related protein 2 3 complex subunit 2-like
Ta_S58888109	2.7	0.0154	TPA: nac transcription factor
contig_2188	2.8	0.0288	Dynamin-like protein
contig_206	3.1	0.0406	Wall-associated receptor kinase 3-like
Ta_S13051479	3.4	0.0261	Serine threonine-protein kinase (afc1)
Ta_S22370925	3.7	0.0285	Cyclin-dependent protein
Ta_S59490003	4.9	0.0402	Transcription factor R2R3-MYB
contig_2559	2.1	0.0428	*lvsA-like*

[a] Fold-change in red indicates down-regulation in colonized roots wheat roots (CWR).
